# Enantiomer-Resolved Biological Profiling and Chiral Developability Assessment of Novel Naphthylethyl Thioureas

**DOI:** 10.3390/pharmaceutics18091129

**Published:** 2026-09-08

**Authors:** Gergely Molnár, Balázs Simon, Arash Mirzahosseini, Máté Dobó, Gergely Dombi, Béla Fiser, Ali Mhammad, Vivien Bárdos, Rita Szolláth, Zoltán-István Szabó, András Marton, Kamilla Varga, Tamás Tábi, Imre Boldizsár, Andrea Horváth, Orsolya Dobay, Szilvia Bősze, György Tibor Balogh, Gergő Tóth

**Affiliations:** 1 Department of Pharmaceutical Chemistry, Semmelweis University, Hőgyes E. Street 9, 1092 Budapest, Hungary; molnar.gergely@stud.semmelweis.hu (G.M.); simon.balazs@semmelweis.hu (B.S.); mirzahosseini.arash@semmelweis.hu (A.M.); dobo.mate@stud.semmelweis.hu (M.D.); dombi.gergely@semmelweis.hu (G.D.); ali.mhammad@phd.semmelweis.hu (A.M.); bardos.vivien@semmelweis.hu (V.B.); szollath.rita.mariann@semmelweis.hu (R.S.); balogh.gyorgy.tibor@semmelweis.hu (G.T.B.); 2 Center for Pharmacology and Drug Research & Development, Semmelweis University, Üllői Str. 26, 1085 Budapest, Hungary; kamillavogelvarga@gmail.com (K.V.); tabi.tamas@semmelweis.hu (T.T.); boldizsar.imre@semmelweis.hu (I.B.); 3 Institute of Chemistry, University of Miskolc, Egyetem Str. 1, 3515 Miskolc, Hungary; bela.fiser@uni-miskolc.hu; 4 Department of Biology and Chemistry, Ferenc Rákóczi II Transcarpathian Hungarian University, Kossuth Square 6, 90202 Beregszász, Ukraine; 5 Department of Physical Chemistry, Faculty of Chemistry, University of Lodz, Pomorska 163/165, 90-236 Lodz, Poland; 6 Department of Industrial Pharmacy and Pharmaceutical Management, Faculty of Pharmacy, George Emil Palade University of Medicine, Pharmacy, Science, and Technology of Targu Mures, Gheorghe Marinescu Street 38, 540142 Târgu Mureș, Romania; zoltan.szabo@umfst.ro; 7 Sz-imfidum Ltd., Lunga nr. 504, 525401 Lunga, Romania; 8 Department of Chemical and Environmental Process Engineering, Faculty of Chemical Technology and Biotechnology, Budapest University of Technology and Economics, Műegyetem rkp. 3., 1111 Budapest, Hungary; amarton@edu.bme.hu; 9 Department of Pharmacodynamics, Semmelweis University, Nagyvárad tér 4, 1089 Budapest, Hungary; 10 Department of Pharmacognosy, Semmelweis University, Üllői út 26, 1085 Budapest, Hungary; 11 Department of Plant Anatomy, Institute of Biology, Eötvös Loránd University, Pázmány Péter Sétány 1/C, 1117 Budapest, Hungary; 12 Institute of Medical Microbiology, Semmelweis University, Nagyvárad tér 4, 1089 Budapest, Hungary; horvath.andrea1@semmelweis.hu (A.H.); dobay.orsolya@semmelweis.hu (O.D.); 13 HUN-REN-ELTE Research Group of Peptide Chemistry, Hungarian Research Network (HUN-REN), Eötvös Loránd University, Pázmány Péter Sétány 1/A, 1117 Budapest, Hungary; szilvia.bosze@ttk.elte.hu; 14 Department of Genetics, Cell- and Immunobiology, Faculty of Medicine, Semmelweis University, Nagyvárad tér 4, 1089 Budapest, Hungary

**Keywords:** thiourea, chiral separation, polar organic mode, enantioselective binding, MRSA, antiproliferative activity

## Abstract

**Background/Objectives:** Stereochemistry can substantially influence the biological activity and pharmaceutical properties of small molecules. This study investigated the stereochemistry-dependent biological activity and chiral recognition of novel naphthylethyl thiourea enantiomers using an integrated biological, chromatographic, and computational approach. **Methods:** Ten pairs of naphthylethyl thiourea enantiomers were synthesized from enantiomerically pure precursors and characterized. Antiproliferative and antibacterial activity, chiral HPLC behavior on polysaccharide- and protein-based stationary phases, molecular docking, and multivariate relationships were evaluated. **Results:** Target-dependent enantioselectivity and substituent-dependent activity patterns were observed. All enantiomeric pairs were chromatographically distinguished under at least one condition, and selected compounds showed selector- and mobile-phase-dependent changes in elution order. AGP-based chromatography showed stereoselective recognition for a subset of compounds, while computational analyses provided complementary support for the experimental trends. **Conclusions:** Stereochemistry and substitution jointly influence biological and chromatographic behavior in this thiourea series. Integrated biological, chromatographic, and computational profiling provides a useful framework for early enantiomer-resolved developability assessment.

## 1. Introduction

Chirality is a fundamental property of many biologically active molecules, and the enantiomers of a chiral compound often exhibit markedly different pharmacological, toxicological, metabolic, and protein-binding profiles. In drug discovery and development, such stereochemistry-dependent differences are highly relevant because the biological behavior of a chiral molecule is determined not only by its chemical constitution, but also by its three-dimensional arrangement. Consequently, the identification of the biologically preferred enantiomer has become an important aspect of lead optimization, safety assessment, and developability evaluation. Accordingly, regulatory agencies increasingly require the characterization and control of enantiomeric purity, together with a clear understanding of the individual properties of enantiomers in biologically relevant systems [1]. From a pharmaceutical perspective, enantiomers may vary in target affinity and efficacy, selectivity, off-target interactions, transport-protein binding, and disposition-related properties. One enantiomer may therefore be mainly responsible for the desired therapeutic effect, whereas its mirror image may show reduced activity, altered selectivity, different pharmacokinetic behavior, or increased toxicity. Stereochemistry-dependent variations are crucial in early-stage drug discovery, since enantiomer-resolved data may improve lead selection and support the development of more effective and safer candidates [1,2,3]. Reliable enantiomer-specific characterization requires robust analytical methods. Chiral separation can be achieved using either indirect or direct analytical approaches. Indirect methods involve derivatization of enantiomers with a chiral reagent to form diastereomers, which can then be separated using conventional achiral techniques. Although these methods may provide high resolution and sensitivity, they require additional sample preparation steps and may introduce bias due to incomplete or non-stereoselective derivatization [4]. By contrast, direct chiral methods rely on chiral selectors, such as chiral stationary phases in chromatography or chiral additives in electrophoretic systems and allow enantiomeric separation without chemical modification of the analyte. These approaches are generally faster, more robust, and better suited for routine stereochemical profiling. Among direct methods, HPLC using chiral stationary phases is considered the gold standard for enantiomeric analysis [5]. Chiral stationary phases derived from polysaccharides are particularly valuable due to their broad enantioselectivity, multimodal interaction mechanisms, and compatibility with various elution modes, including polar organic conditions [6,7]. The polar organic mode, employing neat polar organic solvents like alcohols or acetonitrile, is especially attractive due to its ability to provide short analysis times, optimal peak shapes, high efficiency, favorable analyte solubility, and compatibility with LC–MS, while also offering a greener analytical profile [8,9]. In a development-oriented setting, these features provide chiral HPLC not just a separation instrument, but also a practical platform for rapid stereochemical profiling and compound prioritization. A major concern in chiral separations is the limited prediction of enantioselective behavior [10,11]. Chiral recognition depends on subtle and cooperative interactions between the analyte and selector, where even minor structural modifications may markedly influence selectivity, retention, or the elution order of enantiomers. Therefore, systematic studies of structurally related compounds with a shared common molecular scaffold are particularly informative, as they can elucidate structure–enantioselectivity relationships, support more rational method development, and provide insights into how stereochemical differences may influence biologically relevant behavior [12,13].

Thioureas have gained increasing attention as valuable scaffolds in drug discovery and development due to their facile synthesis and broad range of potential therapeutic uses [2,14]. These compounds have shown potential efficacy in several medical areas, including cholinesterase inhibition for Alzheimer’s disease therapy [15], alongside anti-inflammatory [16], antiviral [17], antibacterial [18], fungicidal [19], and antiproliferative effects [20,21]. Beyond their biomedical relevance, thioureas are also employed in agriculture, industry, analytical chemistry, and as versatile building blocks for the synthesis of other bioactive molecules [22,23]. Collectively, these features provide chiral thioureas particularly attractive targets for stereochemically resolved investigation, especially in studies seeking to link enantiomer-specific biological activity with analytical behavior and early pharmaceutical developability. Importantly, the existing literature already indicates that stereochemistry may strongly influence biological activity in thiourea-based compounds. In a study on chiral naphthyl thioureas as non-nucleoside inhibitors of HIV-1 reverse transcriptase, Venkatachalam and co-workers showed that the asymmetric configuration of the non-nucleoside inhibitor binding site strongly preferred the *R* stereoisomers over their *S* counterparts. Molecular modeling predicted a better fit for the *R* enantiomers, and the experimental results confirmed their superior antiviral potency [24]. In the same line of work, the active R isomers inhibited HIV-1 replication in human peripheral blood mononuclear cells at nanomolar concentrations, whereas the corresponding S enantiomers were inactive or markedly less potent. Moreover, the lead compounds were reported to be approximately three log units more potent than nevirapine against resistant HIV-1 strains, indicating that configuration may influence both potency and the resistance profile inside a thiourea scaffold. Subsequent investigations on related chiral thiourea non-nucleoside reverse transcriptase inhibitors further confirmed the dependency on stereochemistry, revealing that both stereochemistry and regiochemistry profoundly affect anti-HIV activity [25,26]. Beyond the antiviral field, a later anticancer study on chiral thiourea derivatives also demonstrated a measurable stereochemistry–activity relationship, and more recent work has continued to explore enantiomerically pure thioureas in medicinal chemistry settings, including anticholinesterase inhibitor design [14,27]. Collectively, these reports support the view that thiourea-based scaffolds are not only broadly bioactive but also highly suitable for enantiomer-resolved evaluation aimed at discerning the biologically preferred configuration and enhancing early-stage developability assessment.

This study used enantiomerically pure 1-(1-naphthyl)ethylamines as precursors to synthesize a series of novel naphthylethyl thiourea enantiomers in enantiomerically pure form. The individual evaluation of the enantiomers was a primary aim of the study, since stereochemistry can strongly influence biological activity, selectivity, and developability. This work aimed to evaluate the enantiomer-resolved antiproliferative and antibacterial effects of these new thioureas, characterize their relevant physicochemical and chromatographic properties, and develop analytical methods for their stereospecific characterization. In this context, chiral HPLC was applied as an enabling platform for stereochemically resolved profiling, using protein-based stationary phases containing human serum albumin (HSA) and α1-acid glycoprotein (AGP) as surrogate models to probe transport-protein-related recognition. In addition, the reversal of enantiomer elution order under different chromatographic conditions was investigated due to its analytical and mechanistic relevance. To further support the interpretation of the stereochemistry-dependent biological effects, molecular docking and multivariate statistical analysis were also employed to explore structure–activity relationships and the contribution of chromatographic and molecular descriptors to the observed activity patterns. Overall, the study was designed to link stereochemistry-dependent bioactivity with chiral analytical behavior and molecular features to support early-stage pharmaceutical evaluation of this thiourea series.

## 2. Materials and Methods

### 2.1. Materials

The reagents for the synthesis were purchased from Sigma-Aldrich (St. Louis, MO, USA) and TCI Europe (Zwijndrecht, Belgium) and used without further purification. Solvents were freshly distilled prior to use and were dried over anhydrous Na_2_SO_4_. Columns for chiral chromatography Lux Amylose-1 [based on amylose tris(3,5-dimethylphenylcarbamate)], Lux Amylose-2 [amylose tris(5-chloro-2-methylphenylcarbamate)], Lux Cellulose-1 [cellulose tris(3,5-dimethylphenylcarbamate)], Lux Cellulose-2 [cellulose tris(3-chloro-4-methylphenylcarbamate)], Lux Cellulose-3 [cellulose tris(4-methylbenzoate)], Lux Cellulose-4 [cellulose tris(4-chloro-3-methylphenylcarbamate)] were the products of Phenomenex (Torrance, CA, USA). All polysaccharide-based columns had dimensions of 150 × 4.6 mm and a particle size of 5 µm. The chromatographic study of AGP interaction was conducted on a ChromTech Chiral-AGP column (150 mm × 4.0 mm; ChromTech Ltd., Congleton, UK), while the binding study for HSA was performed on a Daicel Chiralpak HSA column (50 mm × 4.0 mm; Daicel Corp., Osaka, Japan). Gradient-grade eluents for HPLC analyses, including methanol (MeOH), acetonitrile (ACN), and 2-propanol, were purchased from Merck (Merck, Darmstadt, Germany). HPLC-grade water was produced using a Millipore Milli-Q Direct-Q 3 UV water purification system (Merck, Darmstadt, Germany).

### 2.2. Synthesis and Structure Elucidation

**Figure 1:**
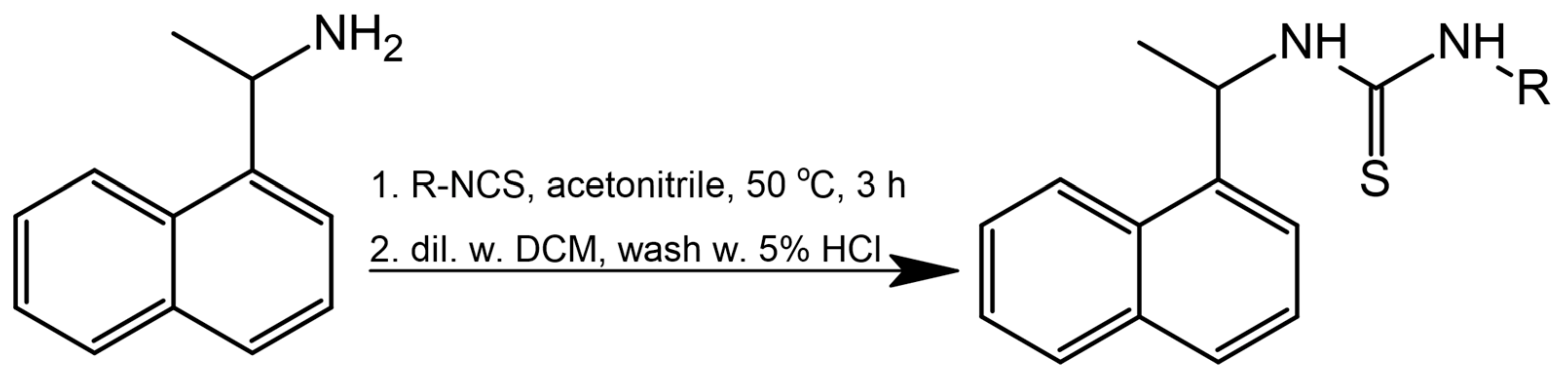
General scheme of the thiourea synthesis.

A general procedure for the synthesis of N,N’-thiourea derivatives was implemented as follows: 1.0 mmol of the appropriate isothiocyanate was measured into a screw-capped bottle and dissolved in 1.5 mL of ACN. 1.1 mmol (188.3 mg or 176.5 µL) of the appropriate enantiomerically pure 1-(1-naphthyl)ethylamine was dissolved in 0.5 mL of ACN and added to the isothiocyanate solution, which was then heated at 50 °C for 3 h. The solution was then diluted with 50 mL of dichloromethane, washed three times with 5% HCl solution, and once with concentrated brine. After washing, the organic phase was dried over anhydrous Na_2_SO_4_, and the solvent was removed using a rotary evaporator, yielding the thioureas as differently colored oils in good yields. The general scheme of the synthesis can be found in Figure 1, while the structure of the newly synthesized compounds is given in Table 1.

^1^H and ^13^C NMR spectra were recorded on a Varian Mercury Plus (400.0 MHz for ^1^H and 100.6 MHz for ^13^C, Varian, Inc., Palo Alto, CA, USA) in DMSO-d_6_ or CDCl_3_ solutions for structure elucidation. Chemical shift (δ) is given in ppm referenced to the residual solvent signal. Mass spectra were recorded on a Waters Alliance e2695 separation module connected to Waters Xevo G2 XS-QToF LCMS system and evaluated using MassLynx 4.1 software (Waters Corp., Milford, MA, USA). The JASCO FT/IR-4600 A instrument with ATR Pro ONE accessory (JASCO Ltd., Tokyo, Japan) was used to measure ATR-FTIR spectra from 400 to 4000 cm^−1^ at a resolution of 4 cm^−1^. Background spectra were recorded and subtracted from the samples. Optical rotation was measured in MeOH at 25 °C using a JASCO P-200 polarimeter (JASCO Ltd., Tokyo, Japan). The detailed spectroscopic data for all thioureas are provided in Supporting Information S1.

### 2.3. HPLC Analysis

LC-UV analyses were conducted on an Agilent 1100 HPLC instrument (Agilent Technologies, Waldbronn, Germany), comprising an inline degasser (G1322A), a quaternary pump (G1311A), an automatic injector (G1329A) equipped with a sample thermostat (G1330A), a column thermostat (G1316A) and a diode array detector (G1315A), controlled by Agilent ChemStation B04.03-SP2 software (Agilent Technologies, Waldbronn, Germany). All experiments were carried out at a constant flow rate of 0.5 mL min^−1^ and a column temperature of 25 °C. Neat methanol, acetonitrile, and 2-propanol were used as mobile phases on the polysaccharide-based chiral stationary phases. For the protein-based columns, different mixtures of 2-propanol and 10 mM phosphate buffer (pH 7.0) were used as mobile phases. Detection was carried out at a wavelength of 210 nm. The resolution (*R*_s_) was calculated according to the following equation:

*R*_s_ = 2(t_2_ − t_1_)/(w_1_ + w_2_), (1)

where t_1_ and t_2_ represent the retention times, and w_1_ and w_2_ correspond to the extrapolated baseline peak widths of the first- and second-eluting enantiomers, respectively.

Stock solutions of each enantiomer were prepared in MeOH at 1 mg mL^−1^. For analysis on the polysaccharide-based columns, further dilutions were prepared in methanol, with the S-enantiomer present at a threefold excess. For the protein-based columns, R-enantiomer-enriched samples were used, allowing unambiguous assignment of the enantiomer peaks. Apparent HSA and AGP binding were estimated from chromatographic retention using the following formula:

(2) $$\%\;binding=100\cdot k^{\prime }/(k^{\prime }+1),$$

(3) $$\mathrm{where}\;k^{\prime }=(t_{R}-t_{0})/t_{0};$$

However, these values should be regarded as HPLC-based surrogate estimates of protein interaction rather than absolute plasma protein binding values.

### 2.4. Cytotoxicity Measurements

C6, HL-60, and 3T3 cell lines (ECACC) were maintained in DMEM/F-12 or DMEM (VWR International, Radnor, PA, USA) supplemented with 10% fetal bovine serum (BioSera Nuaille, France), 1% stable glutamine, 80 μg/mL gentamicin, and 10 μg/mL ciprofloxacin (VWR International). Test compounds were dissolved in DMSO and diluted into culture medium to a final DMSO concentration of 0.5%; vehicle controls received 0.5% DMSO alone. Cell viability was determined using the Resazurin Cell Viability Kit (Cell Signaling Technology, Danvers, MA, USA), which relies on intracellular reductase-mediated reduction of non-fluorescent resazurin to fluorescent resorufin. After 24 h exposure to compounds, the medium was replaced with fresh medium containing 10% resazurin solution (0.15 mg/mL), and fluorescence was measured after 4 h on a Varioskan LUX multimode plate reader (Thermo Fisher Scientific, Waltham, MA, USA) at 530/590 nm (Ex/Em). Viability was expressed as a percentage of vehicle-treated controls. For initial screening, compounds were tested at 0.1, 1, and 10 μM; for any compound producing ≥50% reduction in viability, additional concentrations were evaluated to generate concentration–response curves. Concentration–response curves were fitted by four-parameter nonlinear regression using GraphPad Prism v10 (GraphPad Software, La Jolla, CA, USA), and IC_50_ values were calculated from the fitted curves. The present measurements were performed in parallel with those reported by Pollák et al. [28], using the same assay protocol and screening strategy. For direct comparability, compound 10a reported by Pollák et al. [28], which was evaluated using the same assay protocol, was included as a reference antiproliferative compound.

### 2.5. Antimicrobial Activity

Antibacterial activity of the thioureas was assessed against two well-characterized strains of *Staphylococcus aureus*: one methicillin-sensitive *S. aureus* (MSSA) (ATCC 29213) and one methicillin-resistant *S. aureus* (MRSA) (ATCC 33591) strain, using the broth microdilution method in Mueller–Hinton broth (MH; Biolab, Budapest, Hungary). Fresh overnight cultures were adjusted to a 0.5 McFarland standard in 0.9% NaCl, then diluted 1:100 in MH. Aliquots of 50 μL were dispensed into each well of 96-well round-bottom microplates (SARSTEDT AG & Co. KG, Nümbrecht, Germany). Growth controls (no compound) and sterile controls (no bacteria, no compound) were included in eight replicates per plate. Thioureas were dissolved in DMSO at 20 mM and diluted 1:100 in MH immediately before testing. Ten two-fold serial dilutions were prepared in MH, and 50 μL of each dilution was added to the bacterial suspensions, yielding a final inoculum of 5 × 10^5^ CFU/mL and a test range of 100–0.2 μM. Plates were incubated at 35 °C in ambient air for 18–20 h. The minimum inhibitory concentration (MIC) was defined as the lowest compound concentration preventing visible growth. Optical density at 595 nm was measured on a Thermo Scientific Multiskan FC plate reader. All measurements were performed in duplicate; mean bacterial densities relative to untreated controls, with standard deviations, were plotted. Vancomycin was tested in parallel as a reference anti-staphylococcal compound, yielding MIC values of 1.3 μg/mL (0.90 μM) against *S. aureus* ATCC 29213 and 1.5 μg/mL (1.04 μM) against MRSA ATCC 33591. Oxacillin was used to confirm the methicillin-susceptible or methicillin-resistant phenotype of the reference strains.

### 2.6. Statistical Analysis

All statistical analyses were conducted in R (version 4.5.2) (R Foundation for Statistical Computing, Vienna, Austria) using base functionality and selected packages from the R ecosystem in RStudio 2026.01.2 (Posit, PBC, Boston, MA, USA). Data manipulation and aggregation were performed using data.table, and visualization was produced using ggplot2 and related plotting utilities [29].

To explore relationships between biological activity, chromatographic behavior, and computed molecular descriptors, Spearman rank correlation coefficients were calculated between variable groups and depicted using igraph 2.2.1 [30].

To investigate multivariate relationships between predictors and the biological outcome, Partial Least Squares (PLS, using pls) regression was applied using the combined HPLC and Percepta descriptor matrix as predictors and the biological index as the response variable. Predictor variables were centered and scaled prior to modeling. PLS components were extracted sequentially to maximize the covariance between predictors and the response. Model interpretation focused on the first two latent components, visualized as a loading map, together with Variable Importance in Projection (VIP) scores to assess the relative contribution of predictors to the model [31].

### 2.7. Molecular Docking

Molecular docking was carried out with two main objectives: first, to explore the enantioselective interactions of the thiourea derivatives with AGP and thereby obtain insight into their stereoselective binding properties; and second, to explore interactions with selected antimicrobial target models and assess the enantioselective binding of the compounds to these targets by molecular modelling. Accordingly, five target structures were selected, namely isoleucyl-tRNA synthetase, penicillin-binding protein, DNA gyrase, and two human AGP isoforms. Their structures were obtained from the RCSB Protein Data Bank with the PDB IDs 1JZS, 1MWT, 2XCQ, 3APW, and 3KQ0, respectively. Ten enantiomeric pairs of thiourea derivatives were included in the docking studies as ligands. These calculations were used as exploratory structural models and were not intended to establish the biological targets responsible for the observed antibacterial activity.

The protein structures were prepared by employing the Protein Preparation Wizard of the Schrödinger Suite [32]. In four cases (isoleucyl-tRNA synthetase, penicillin-binding protein, and two AGP structures), the co-crystallized ligand was applied from the original PDB structures to define the target area for docking. In the case of the DNA gyrase, the original structure (PDB ID: 2XCQ) does not include a ligand and thus, the target area was specified by using specific residues (GLY1082, ASP1083 and SER1084) which are known to interact with ligands. After defining the target areas, the Receptor Grid Generation module of the Schrödinger Suite was employed to create the docking grid. The thiourea derivative pairs were prepared for docking by using the LigPrep module. Docking calculations were carried out by using flexible ligand sampling and the standard precision (SP) mode of Glide [33,34,35]. For comparison, the docking scores were collected and analyzed along with the corresponding complexes. For visualization, Maestro version 14.5.131 was employed within the 2025-3 release of the Schrödinger Suite.

## 3. Results and Discussion

### 3.1. Enantioseparation on Polysaccharide-Type Chiral Stationary Phase

**Figure 2:**
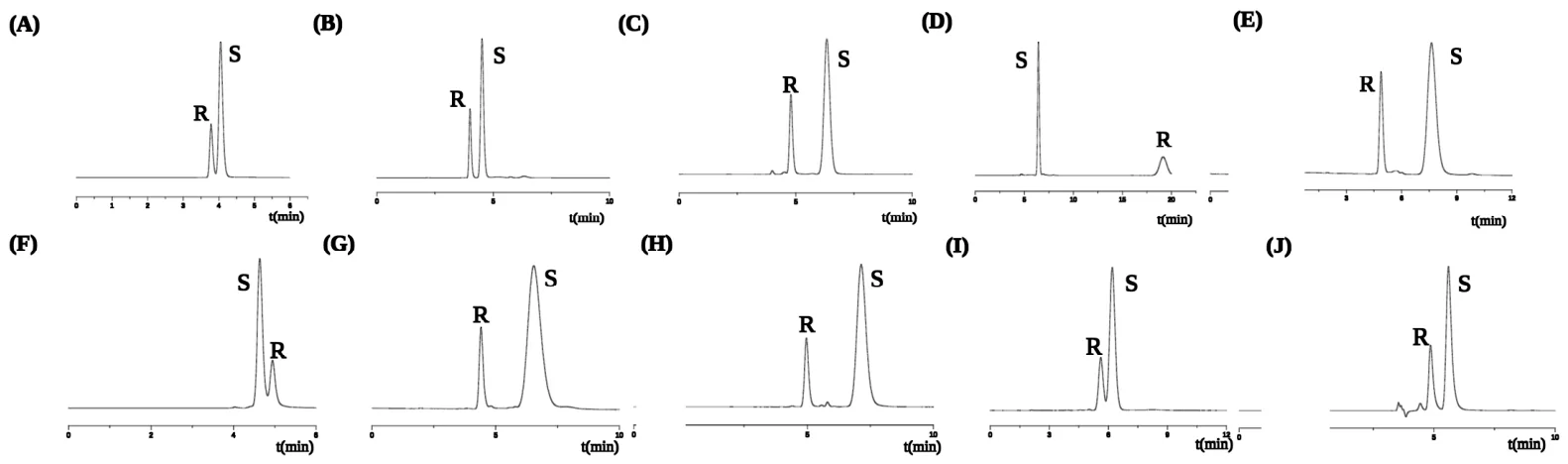
Representative chromatograms corresponding to the highest-resolution separation obtained for each enantiomeric pair. (**A**): Compound **1** with Amylose-1 CSP and methanol eluent, (**B**): Compound **2** with Amylose-1 CSP and methanol eluent, (**C**): Compound **3** with Amylose-1 CSP and methanol eluent, (**D**): Compound **4** with Cellulose-3 and methanol eluent, (**E**): Compound **5** with Amylose-1 CSP and methanol eluent, (**F**): Compound **6** with Cellulose-2 and acetonitrile eluent, (**G**): Compound **7** with Amylose-1 CSP and methanol eluent, (**H**): Compound **8** with Amylose-1 CSP and methanol eluent, (**I**): Compound **9** with Amylose-1 CSP and methanol eluent, (**J**): Compound **10** with Cellulose-1 CSP and 2-propanol eluent. Flow rate: 0.5 mL/min, temperature: 25 °C.

Chiral HPLC screening was performed to determine whether the newly synthesized thiourea enantiomer pairs could be resolved under practical conditions, to identify chromatographic trends relevant to stereochemically resolved biological evaluation, and to assess the suitability of the method for enantiomeric purity control. Screening was carried out on six polysaccharide-based chiral stationary phases in polar organic mode using MeOH, ACN, and 2-propanol as eluents. All ten enantiomeric pairs were successfully separated, with each compound resolved under at least one chromatographic condition. Enantiorecognition was observed in 87 of 180 experiments, and baseline separation was achieved in 38 cases, confirming broad analytical applicability of the screening strategy. Overall, these results demonstrate that polar organic chiral HPLC is a practical platform for enantiomer-specific profiling and enantiomeric purity assessment of this thiourea series. The outcomes of this screening are detailed in Supplementary Tables S1 and S2. Figure 2 shows representative chromatograms of the highest-resolution separations obtained for each enantiomeric pair.

**Figure 3:**
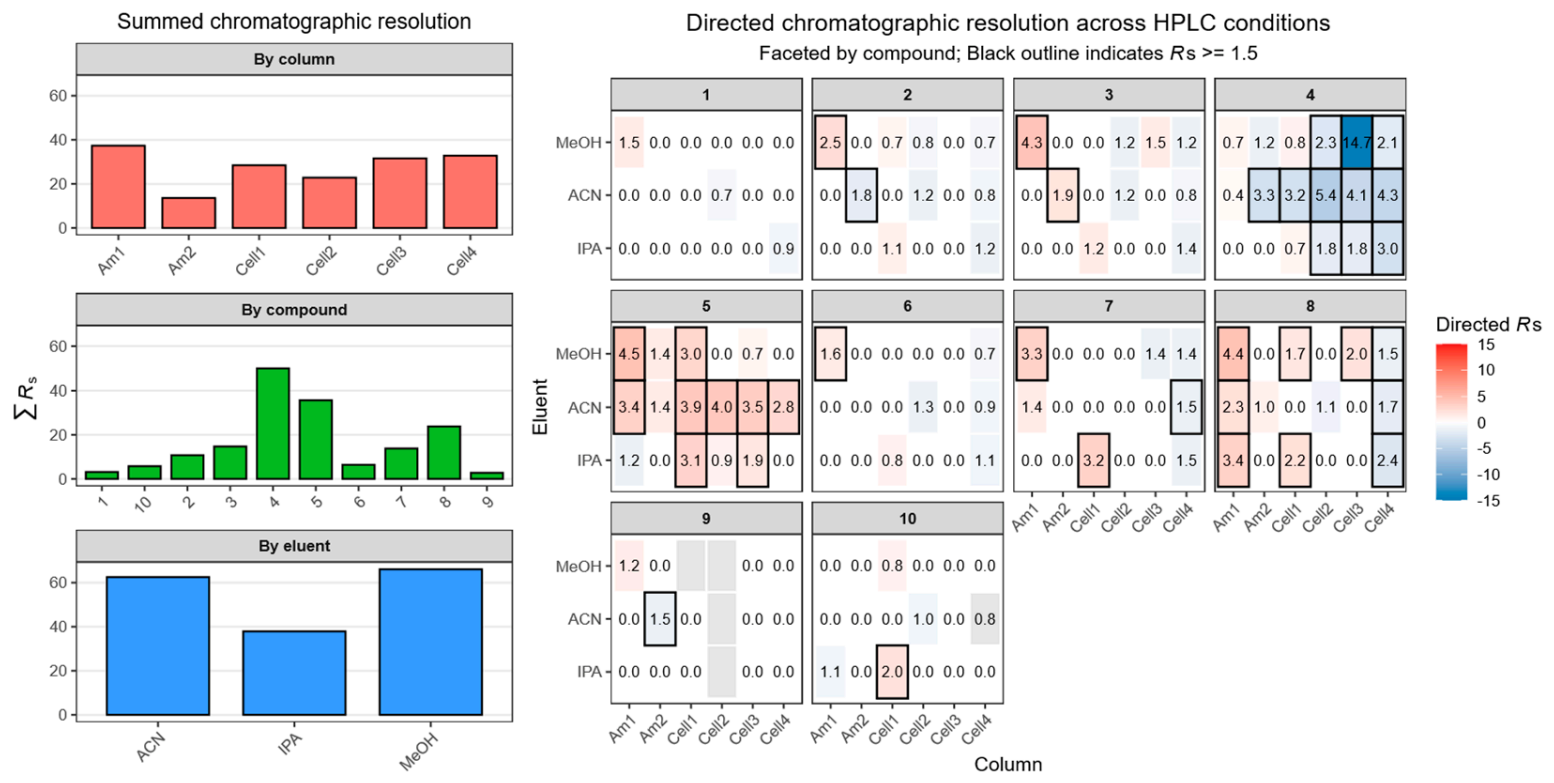
(**Left**): Cumulative chromatographic resolution (ΣRs) grouped by stationary phase, compound, and eluent. (**Right**): Directed chromatographic resolution across the investigated compound–eluent–CSP combinations.

Analysis times ranged from 3.57 to 19.16 min, with a mean of 5.33 min over the entire screening dataset, thereby confirming the practical advantage of polar organic mode for rapid enantiomeric screening. Of the eluents investigated, 2-propanol provided the shortest and most consistent analysis durations, whereas methanol and acetonitrile typically afforded superior cumulative chromatographic resolution. Detailed analysis-time statistics are provided in Supporting Information S2 and Supplementary Figure S1. The screening dataset was further evaluated using cumulative resolution values, calculated as the sum of the individual $R_{s}$ values ($\Sigma R_{s}$) across all screening measures. Among the investigated eluents, methanol provided the highest overall resolving power ($\Sigma R_{s}=67.3$), closely followed by acetonitrile ($\Sigma R_{s}=63.1$), whereas 2-propanol gave the lowest cumulative resolution ($\Sigma R_{s}=37.9$). The same trend was observed for the stationary phases: Lux Amylose-1 showed the highest overall performance ($\Sigma R_{s}=37.3$), whilst Lux Amylose-2 had the lowest efficacy ($\Sigma R_{s}=13.6$); the cellulose-based CSPs revealed intermediate performance (Figure 3).

At the compound level, enantioseparation performance was greatest for compound 4 ($\Sigma R_{s}=49.9$), followed by compounds 5 ($\Sigma R_{s}=35.6$) and 8 ($\Sigma R_{s}=23.8$) (Figure 3). Analysis of the structures suggests that phenyl substitution is favorable for enantiorecognition in this thiourea series, but direct halogen substitution on the phenyl ring further enhances separation performance. Consistent with this trend, compound 1, with an isopropyl side chain, showed the lowest $\Sigma R_{s}$ value, whereas compound 9, despite containing a phenyl substituent, likewise demonstrated poor performance in the absence of halogen substitution. An additional substituent-position effect was observed for the trifluoromethylphenyl analogues, as the para-substituted compound 7 showed an almost fourfold greater $\Sigma R_{s}$ value than its ortho-substituted analogue 10. Overall, these findings indicate that aromatic substitution patterns significantly influence chromatographic enantiorecognition within this thiourea series.

Furthermore, directed resolution was calculated to provide a signed measure of chromatographic separation for the enantiomeric elution order. The conventional chromatographic resolution ($R_{s}$) is defined as a non-negative value that reflects the extent of peak separation but does not provide information on elution order. Directed resolution was determined by assigning the conventional resolution a sign based on the elution order of the peaks: positive for R–S and negative for S–R. Thus, directed $R_{s}$ allows correlations and multivariate visualizations that distinguish not just between strong and weak separations but also reversals in elution order. Although changes in stationary phase are well known to alter enantiomer elution order, and numerous examples of such behavior have been reported in the literature, mobile-phase-dependent reversal is generally considered more noteworthy [36,37]. In the present dataset, mobile-phase-dependent enantiomer elution order reversal was observed only in two cases: for enantiomer pair **5** on the Lux Amylose-1 CSP, the order was R–S in methanol and acetonitrile but switched to S–R in 2-propanol, whereas for enantiomer pair **4** on the Lux Cellulose-1 CSP, the order was S–R in acetonitrile and R–S in methanol and 2-propanol. Detailed examples of these elution-order reversals are provided in the Supporting Information S3 and Supplementary Figures S2–S6. The analyte substituent pattern also influenced the observed enantiomer elution order. Under acetonitrile conditions on the investigated cellulose-based stationary phases, the iodophenyl derivative 4 predominantly showed S–R elution, whereas the dichlorophenyl derivative 5 predominantly showed the opposite R–S order. Thus, modification of the aromatic substituent can alter the preferred direction of chiral recognition under comparable chromatographic conditions. Nevertheless, the mobile-phase-dependent reversals described above show that elution order is not determined by analyte substitution alone, but by the combined effects of substituent structure, chiral selector, and mobile phase; these factors should therefore be considered together during stationary-phase and eluent selection.

### 3.2. Transport Protein Binding: HPLC and Molecular Docking Study

**Figure 4:**
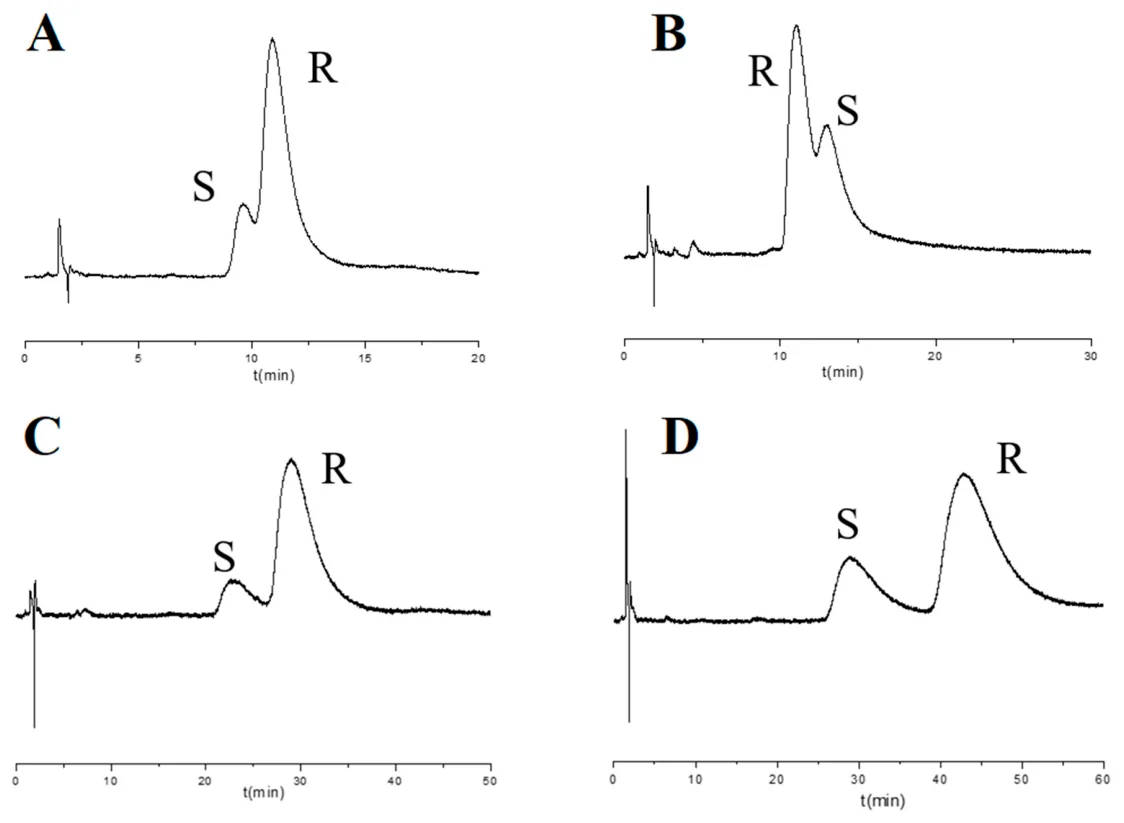
Representative chromatograms of compounds 2, 3, 8, and 10, for which enantiorecognition was observed on the AGP column. (**A**,**B**) were recorded using 10 mM phosphate buffer/2-propanol (90:10, *v*/*v*; pH 7.0), whereas (**C**,**D**) were recorded using 10 mM phosphate buffer/2-propanol (85:15, *v*/*v*; pH 7.0) as the mobile phase.

The stereoselective interaction with plasma transport proteins holds considerable pharmacokinetic relevance as it can influence the free drug fraction, distribution, and pharmacokinetic behavior [38]. This study investigated the effects of two protein-based chiral stationary phases: HSA and AGP. These columns served as surrogate chromatographic models for transport-protein binding, and HPLC-derived apparent HSA and AGP binding values were determined from retention data [39,40]. Only compounds 1, 2, 3, 6, 8, 10 were included in this part of the study due to solubility limitations. Enantiorecognition was observed on the AGP-based column in four instances, demonstrating stereoselective interaction with this protein model (Figure 4). In three of these cases (compounds 2, 8, 10), the *R*-enantiomer showed greater apparent affinity, while compound 3 displayed the opposite trend, with the *S*-enantiomer being more strongly retained. In contrast, no enantiorecognition was detected on the HSA-based column for any of the investigated thiourea pairings. These results indicate that AGP-related recognition exhibits greater sensitivity to stereochemical differences within this thiourea subset compared to the HSA model.

The apparent interaction indices derived from HPLC retention were calculated using Equation 2 and are summarized in Table 2. Apparent HSA-derived values ranged from 89.33% to 97.53%, while AGP-derived values ranged from 91.58% to 98.05%. Because these values were obtained on different immobilized protein stationary phases with distinct chromatographic characteristics, the absolute percentages should not be interpreted as directly comparable measures of plasma protein binding. The most informative stereochemical observation was the enantiorecognition detected on the AGP column, whereas no enantiorecognition was observed on HSA under the investigated conditions. Among the analyzed compounds, compound 1 showed the lowest retention-derived apparent interaction values on both protein-based columns, whereas compounds 8 and 10, which are characterized by aromatic substituents and increased lipophilicity, exhibited the highest values. These results should therefore be regarded as chromatographic surrogate measures of interaction with the immobilized protein models rather than absolute plasma protein binding parameters.

**Figure 5:**
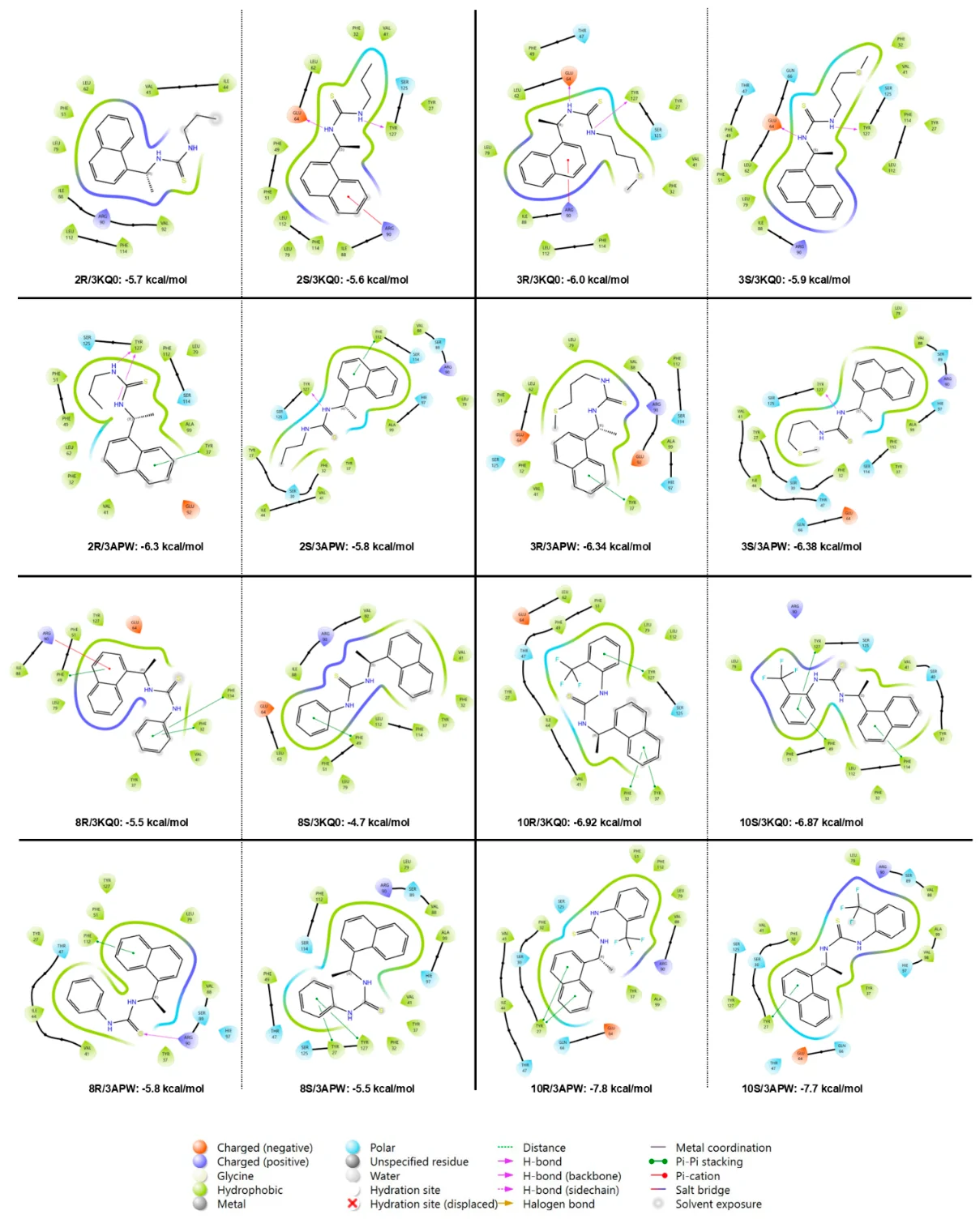
Ligand interaction maps of compounds **2, 3, 8**, and **10** bound to the two human α1-acid glycoprotein isoforms. The corresponding PDB IDs are 3APW and 3KQ0.

Molecular docking studies were performed using two human AGP isoforms as target structures to further support the interpretation of the experimentally observed AGP-related stereoselective recognition. These calculations were used to assess if the experimentally observed retention trends could be rationalized at the molecular level and if the preferred enantiomers on the AGP column also showed more favorable predicted binding. The Glide docking scores of the four enantiomeric pairs are summarized in Table 3, and ligand interaction maps are shown in Figure 5.

The docking results suggest that the observed stereoselective AGP recognition mostly stems from minor differences in binding geometry and hydrophobic pocket complementarity rather than from fundamentally different interaction types. In most cases, the R-enantiomers adopted slightly more favorable conformations, aligning with their experimentally enhanced AGP-related retention on the AGP-based column. This agreement was observed for compounds **2**, **8**, and **10**, wherein the R-enantiomers showed more favorable docking scores than the corresponding S-forms on both AGP models (3APW and 3KQ0). The clearest discrimination was found for compound **8**, particularly on the 3KQ0 structure, whereas for compound **10** the calculated enantiomeric difference was small but still correctly reproduced the experimentally observed ranking. The interaction maps indicate that these stereoselective differences are mainly associated with altered spatial orientation of the thiourea core and aromatic moieties inside the AGP binding pocket, resulting in minor yet consistent changes in hydrophobic complementarity and polar contact geometry. Compound **3** was an exception, for which the calculated docking scores of the two enantiomers were nearly identical and slightly favored the R-enantiomer, whereas experimentally the S-enantiomer showed stronger AGP-related retention. This suggests that the experimentally observed stereoselective recognition of compound **3** is only weakly reflected by the docking model, possibly because the more flexible side chain enables both enantiomers to adopt similarly favorable binding arrangements within the AGP pocket. Overall, the docking results reproduced the experimental AGP-related stereoselective trend in most, but not all, cases, indicating that chiral recognition in this thiourea subset is driven by fine differences in pocket fit rather than by large differences in calculated docking score.

### 3.3. Results of Biological Testing

#### 3.3.1. Cytotoxicity Data

The antiproliferative activity of the synthesized compounds was evaluated on two cancer cell lines: HL-60 promyelocytic leukemia cells (representing disseminated cancer with no surgical treatment, where systemic therapy is pivotal) and C6 glioma cells (a CNS solid-tumor model with limited therapeutic options). NIH 3T3 fibroblasts were included as non-malignant controls to assess cancer selectivity. The IC50 values are summarized in Table 4. For comparison, the reference compound 10a [28] showed IC_50_ values of 57.97 nM and 93.41 nM against C6 and HL-60 cells, respectively, while its IC_50_ value in non-malignant 3T3 fibroblasts was 332.5 nM.

Antiproliferative profiling revealed a clear cytotoxicity pattern on non-malignant NIH 3T3 fibroblasts: in several series the R-enantiomers exhibited substantially higher general cytotoxicity than their S counterparts (e.g., (R)-1: 37.23 nM vs. (S)-1: 279.6 nM; (R)-10: 50.8 nM vs. (S)-10: 210.3 nM; (R)-2: 149.5 nM vs. (S)-2: 350.8 nM; (R)-4: 346.3 nM vs. (S)-4: 507.4 nM). A notable exception is (S)-6, which is more cytotoxic than (R)-6 (211.7 vs. 718.7 nM), underscoring that the direction of enantioselective cytotoxicity can be compound-specific. The pronounced effect on non-cancer cells indicates an elevated risk of off-target toxicity, particularly for (R)-1 and (R)-10, which are strikingly potent on 3T3 cells.

Although tumor lines showed generally lower sensitivity to the antiproliferative effect of the examined compounds, enantioselectivity in their effect is also pronounced. In C6 glioma cells, S-enantiomers are generally more potent ((S)-5: 45.81 nM ≫ (R)-5: 611.2 nM; (S)-7: 199.2 nM > (R)-7: 272.3 nM; (S)-4: 224.3 nM), whereas in HL-60 leukemia cells R-enantiomers are stronger ((R)-4: 84.85 nM; (R)-5: 157.1 nM; (R)-8: 271.1 nM > (S)-8: 359.1 nM). On the other hand, some compounds showed appreciable tumor selectivity. The selectivity index (SI = IC_50_(3T3)/IC_50_[tumor]) indicates selectivity toward leukemia cells: (R)-8 shows SI ≈ 9.1, (R)-4 SI ≈ 4.1. For glioma cells, (S)-4 affords a moderate tumor selectivity: SI ≈ 2.26, while no IC50 could be determined for (S)-5 on 3T3 cells within the tested range, indicating selectivity toward C6 cells under the investigated in vitro conditions. In summary, cytotoxicity is enantioselective, typically R-enantiomers are more potent on 3T3 fibroblasts, while tumor-selective activity tends to favor S-enantiomers in C6 and R-enantiomers in HL-60 cells. Within the investigated in vitro models, these patterns identify (S)-5 as a candidate for further evaluation in C6 cells and (R)-4/(R)-8 as candidates for further evaluation in HL-60 cells, while the pronounced 3T3 potency of (R)-1 and (R)-10 warrants caution.

#### 3.3.2. Antimicrobial Effects

The antibacterial activity of the synthesized thiourea enantiomers was evaluated against methicillin-sensitive (MSSA) and methicillin-resistant *Staphylococcus aureus* (MRSA) strains to assess both overall potency and possible stereochemistry-dependent differences in activity. The results, expressed as minimum inhibitory concentration (MIC) values, are summarized in Table 5.

Minimum inhibitory concentration (MIC) profiling against methicillin-sensitive (*S. aureus* MSSA) and methicillin-resistant (*S. aureus* MRSA) strains revealed three recurring patterns. First, the enantiomeric pair of compounds 7 showed clear S-enantiomer dominance, with (S)-7 emerging as the most active anti-MRSA analogue (MIC = 3.125 μM) while showing the same activity as (R)-7 against MSSA (6.25 μM). The 8-fold difference observed on MRSA indicates pronounced, strain-dependent enantioselectivity, plausibly reflecting stereospecific target engagement or uptake. Second, compounds 4 and 5 showed a consistent R-enantiomer preference, with (R)-4 and (R)-5 displaying balanced dual-strain activity (12.5/12.5 μM), whereas (S)-4 (25/25 μM) and (S)-5 (12.5/25 μM) were weaker or less balanced. Third, several compounds showed weak MSSA-only activity confined to the S-enantiomers ((S)-8: 50 μM; (S)-10: 50 μM; (S)-2: 100 μM; (S)-9: 100 μM), while the corresponding R-enantiomers were inactive; these profiles appear to be of lower development priority unless accompanied by exceptional safety or pharmacokinetic advantages. Ranking by MRSA potency places (S)-7 clearly first, followed by (R)-4 ≈ (R)-5, then (S)-5 ≈ (S)-4; by MSSA potency, (S)-7 = (R)-7 lead, followed by (R)-4 = (R)-5 = (S)-5, then (S)-4, and finally (S)-8/(S)-10 and (S)-2/(S)-9. Collectively, these data indicate that stereochemistry substantially influences antibacterial activity within this thiourea series.

Beyond stereochemical effects, the biological data also indicate a substituent-dependent structure–activity relationship. Compounds bearing predominantly aliphatic substituents (1–3 and 6) generally showed little or no antiproliferative activity against C6 and HL-60 cells and limited antibacterial activity, whereas several aromatic, particularly halogenated, derivatives (4, 5, and 7) displayed higher activity. A clear positional effect was observed for the trifluoromethylphenyl analogues, with the para-substituted compound 7 showing substantially greater antiproliferative and antibacterial activity than the ortho-substituted compound 10. The more active aromatic and halogenated derivatives also generally showed higher predicted lipophilicity than the aliphatic analogues; however, this relationship was not monotonic, and logP was not an important variable in the PLS analysis. Thus, lipophilicity may contribute to the observed SAR but does not appear to be its primary determinant.

**Figure 6:**
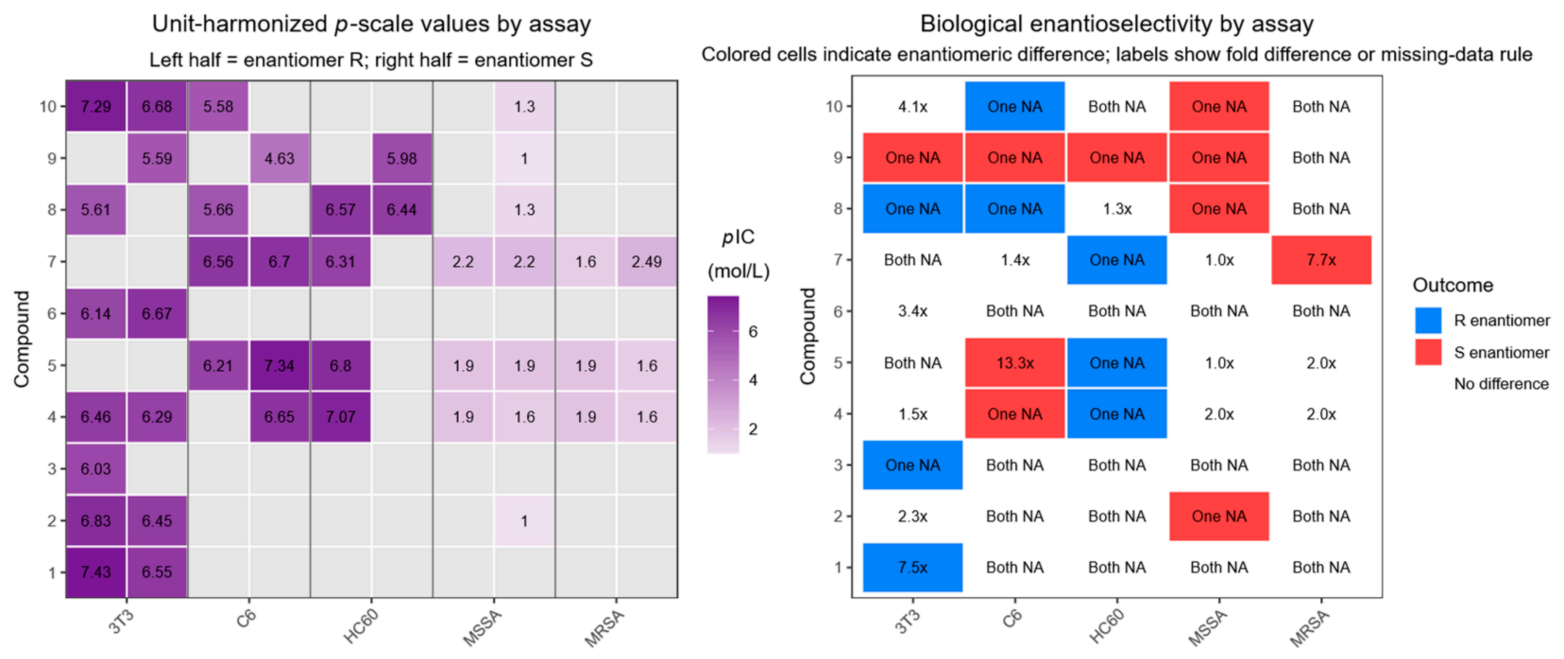
(**Left**): Heatmap of the transformed endpoint concentration after conversion to molar units. For compactness, the figure labels this quantity pIC; it represents −log_10_ of the molar IC_50_ or MIC value, as applicable, and is used for visualization only. Larger values indicate lower endpoint concentrations. (**Right**): Binary “has difference” indicator showing assays where the endpoint concentrations of the two enantiomers differ by at least a fivefold ratio. Colors indicate which enantiomer has higher potency within the same assay.

To provide a unit-consistent visualization across the different assays, the measured endpoint concentrations (IC_50_ or MIC, as applicable) were first converted to molar units and then transformed as −log_10_ of the endpoint concentration. In Figure 6, this transformed quantity is labeled pIC for compactness. Because MIC and IC_50_ are distinct biological endpoints, this transformation was used only to harmonize the numerical scale for visualization and does not imply biological equivalence or direct mechanistic comparability between the assay types. Higher transformed values correspond to lower endpoint concentrations. To complement the continuous heatmap (Figure 6, left), a categorical “difference in potency” indicator was also computed (Figure 6, right). Within each assay, enantiomeric potency was considered meaningfully different if the ratio between their endpoint concentrations was at least fivefold, or if exactly one of the enantiomers had a measurable effect while its counterpart had none.

Molecular docking calculations were carried out to explore the interactions between thiourea derivatives and a diverse range of biological targets including isoleucyl-tRNA synthetase (1JZS), penicillin-binding protein (1MWT), and DNA gyrase (2XCQ).

**Figure 7:**
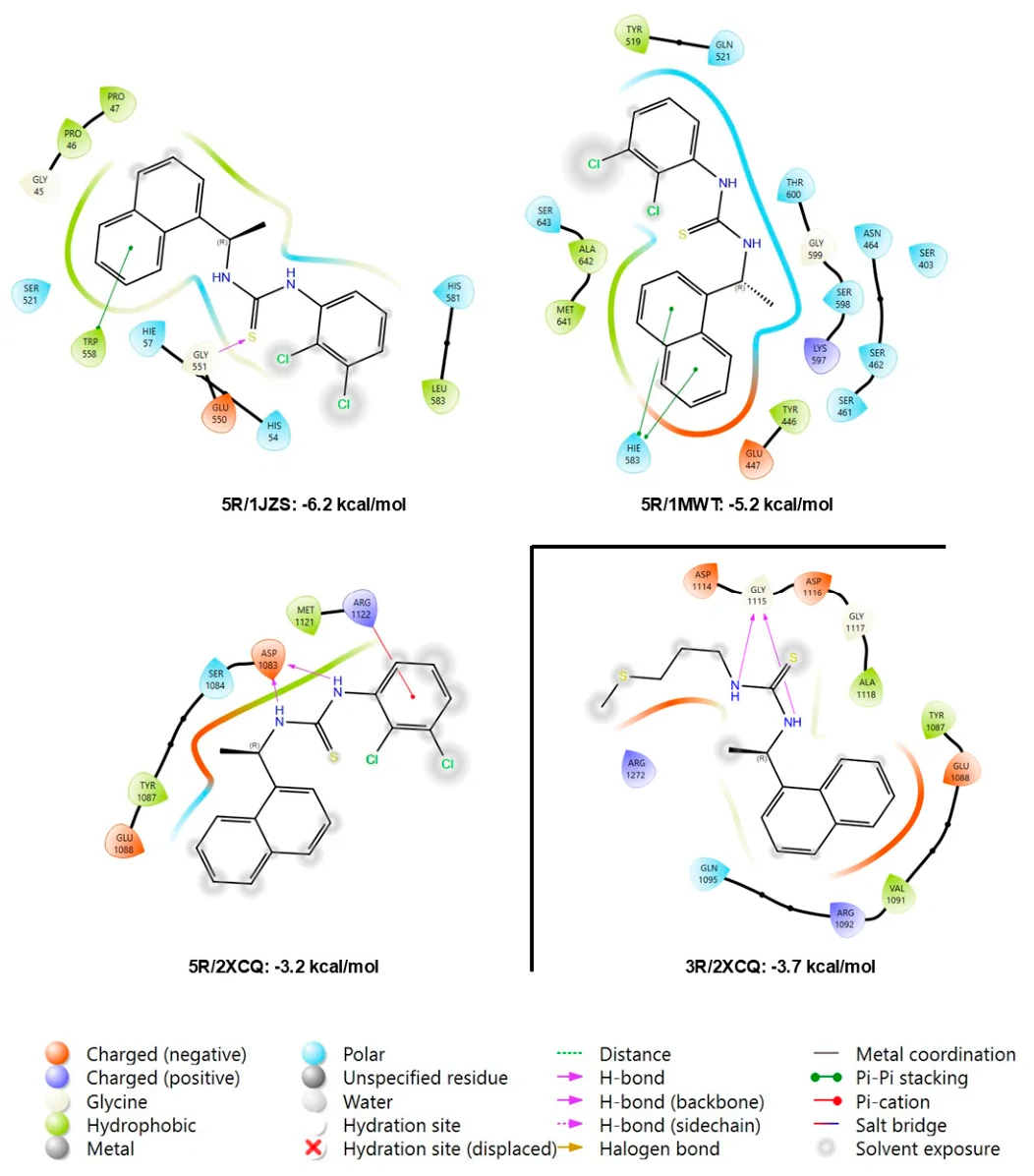
Ligand interaction maps of the docked thiourea derivatives showing the most favorable interactions with DNA gyrase, penicillin-binding protein, and isoleucyl-tRNA synthetase. The corresponding PDB IDs are 2XCQ, 1MWT, and 1JZS, respectively. For 1MWT and 1JZS, the top docking scores were obtained for the 5-series derivatives, whereas for 2XCQ the most favorable score was observed for R-configured ligands. Because (R)-3 was selected as the representative ligand for DNA gyrase, the interaction patterns of both (R)-3 and (R)-5 are shown for comparison in this target.

Docking against penicillin-binding protein (1MWT), isoleucyl-tRNA synthetase (1JZS), and DNA gyrase (2XCQ) revealed target-specific selectivity within the investigated thiourea series. The most favorable docking scores for penicillin-binding protein and isoleucyl-tRNA synthetase were obtained with compound **5**, where both enantiomers gave identical top-ranked values of −5.2 and −6.2 kcal/mol, respectively (Figure 7, Table 6). This suggests that these two binding pockets are particularly suitable for the 5-series scaffold. The favorable binding may arise from the orientation of the thiourea core, which supports hydrogen-bonding interactions via the NH and C = S functionalities, while the aromatic rings and halogen substituents may contribute additional hydrophobic and π-related contacts. In contrast, for DNA gyrase (2XCQ), the most favorable docking score (−3.7 kcal/mol) was observed for the R-enantiomers of compounds 1 and 3. Among these, compound (R)-3 was selected for further interaction analysis (Figure 7), as its binding mode suggests a more suitable arrangement within the active site than that of compound 5, enabling a more favorable combination of hydrogen-bonding and electrostatic interactions. Overall, these results indicate that binding to penicillin-binding protein and isoleucyl-tRNA synthetase is driven primarily by scaffold complementarity, whereas stereochemistry appears to have a more pronounced impact on the DNA gyrase model.

### 3.4. Multivariate Analysis

#### 3.4.1. Construction of Summary Variables for Further Analysis

The raw chromatographic and biological measurements were condensed into a set of compound-level summary descriptors to enable meaningful cross-domain comparison. Multiple measurements for each compound across different chromatographic settings and biological assays would introduce redundancy into the correlation search. Therefore, for each compound, aggregated variables were constructed that capture the overall chromatographic separation behavior and the general tendency for enantiomeric biological differences.

The total number of distinct experimental conditions in the chromatographic dataset was defined by the number of unique eluent–column combinations. Using this reference set, compound-level descriptors were calculated to summarize separation performance across all conditions. These included the maximum and mean chromatographic resolution (max_Rs, mean_Rs), the number of conditions yielding baseline separation (n_sep_1.5), and the fraction of conditions where each enantiomer eluted first (frac_EEO1, frac_EEO2), normalized by the total number of tested chromatographic conditions. Together, these descriptors characterize the strength and directional preference of enantiomeric separation across the HPLC screening space.

Similarly, biological activity data were summarized across assays to capture the global pattern of enantiomeric differences. For each compound, the number of assays showing a meaningful enantiomeric potency difference (n_difference) was calculated, together with the fraction of assays in which each enantiomer exhibited higher potency (frac_e1_win, frac_e2_win). These variables therefore quantify the prevalence and direction of stereoselective biological effects across the assay panel.

In addition to the experimental dataset, ACD/Percepta-calculated physicochemical, ADME-related, and toxicity-related descriptors were also included in the subsequent analyses; the full set of descriptors is provided in Supplementary Table S3. Transforming the initial dataset into compound-level summary variables provided a compact and interpretable matrix of chromatographic, biological, and molecular properties suitable for subsequent correlation and multivariate analyses.

#### 3.4.2. Correlation Analysis

**Figure 8:**
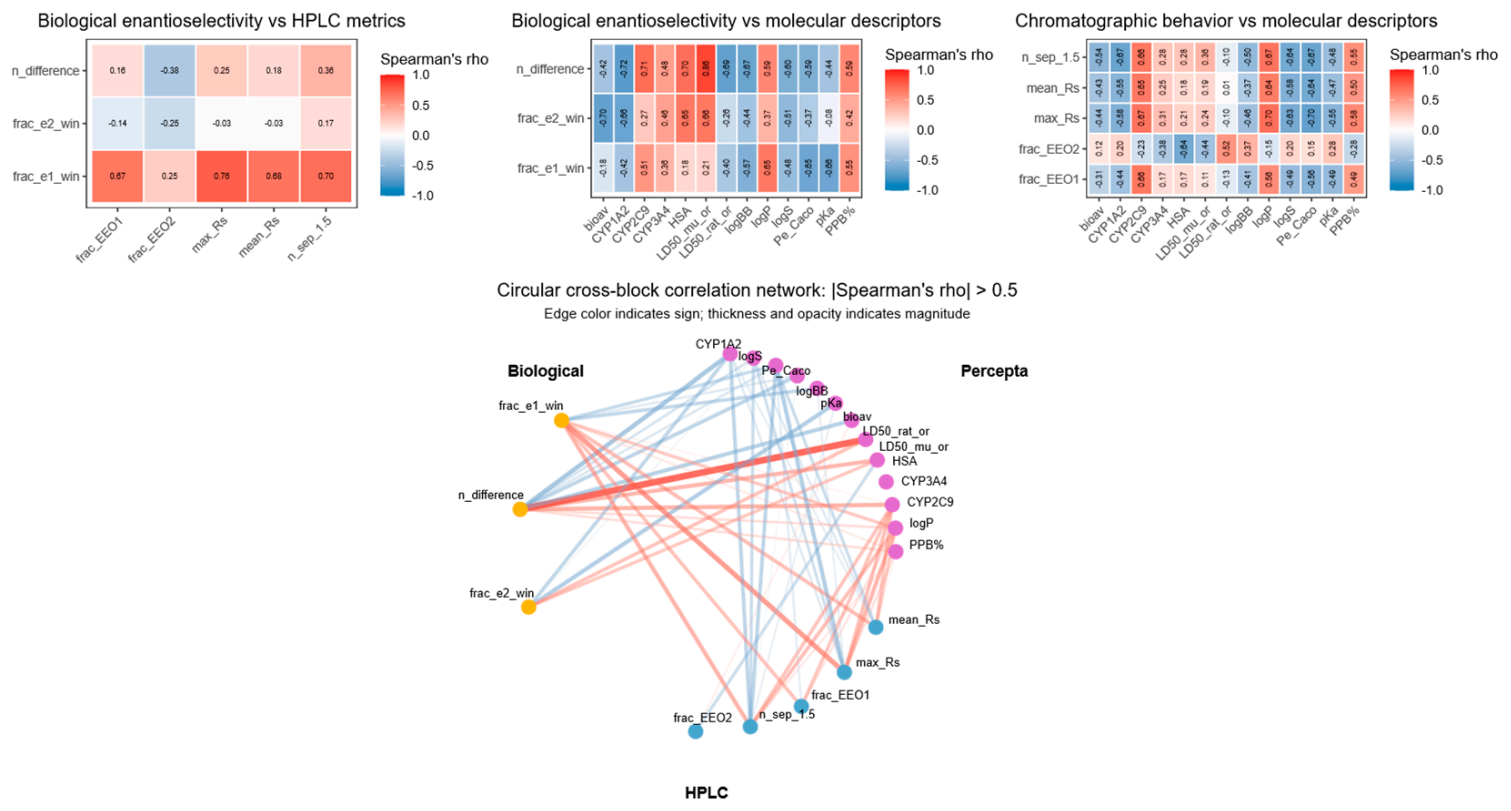
Top panels: Heatmaps of Spearman rank correlation coefficients (rho) calculated between variables from different domains: biological indices, HPLC parameters, Percepta molecular descriptors. The color scale indicates the strength and direction of the relationship. Bottom panel: Three-layer correlation network summarizing the same relationships. Nodes represent variables from the bio, HPLC, and Percepta sets, arranged in separate regions, while edges connect variables with thickness proportional to the magnitude of the Spearman correlation coefficient.

A systematic Spearman rank correlation analysis was performed to explore potential relationships among biological activity, chromatographic behavior, and computed molecular descriptors, across three variable groups: biological indices (bio), experimental chromatographic parameters (HPLC), and calculated physicochemical descriptors obtained from ACD/Percepta. It should be noted that the Percepta analysis is unable to distinguish between the individual enantiomers. Spearman’s rho was selected because of its ability to identify monotonic relationships without assuming linearity or normal distributions. Pairwise correlations were calculated across variables from different groups, and the results are summarized in three heatmaps showing the cross-domain associations (Figure 8).

In addition to the matrix representation, the correlations were also represented as a three-layer network, where each layer corresponds to one variable class (bio, HPLC, or ACD/Percepta). Nodes represent individual variables, while edges connect variables across layers based on their Spearman correlation coefficient. The thickness and opacity of edges reflect the magnitude of the correlation, allowing clear differentiation between robust and weaker relationships.

The cross-domain correlation patterns suggest that the biological and chromatographic variables were not uniformly related. Of the biological indices, frac_e1_win had the clearest association with the HPLC-derived metrics, with high correlations with chromatographic resolution and elution-order parameters, whereas n_difference and frac_e2_win showed weaker or less consistent relationships. In contrast, the ACD/Percepta descriptors exhibited a broader correlation with the biological indices aside from frac_e1_win; although most correlations were moderate and dependent on the specific descriptor, stronger associations were observed for descriptors related to predicted toxicity (LD50), CYP-mediated metabolism, lipophilicity (logP), and blood–brain barrier penetration (logBB). The HPLC–ACD/Percepta correlation map further indicated that predicted bioavailability, CYP-related descriptors, and lipophilicity were the descriptors most consistently associated with chromatographic behavior, showing correlations with various HPLC metrics. Overall, these exploratory results suggest that chromatographic elution-order tendencies may provide valuable insights into biological enantioselectivity, while broader patterns of biological activity and chromatographic performance seem to be influenced by physicochemical and pharmacokinetic descriptor space. Due to the limited number of compounds, these associations should be regarded as hypothesis-generating rather than confirming.

#### 3.4.3. Partial Least Squares Fit

**Figure 9:**
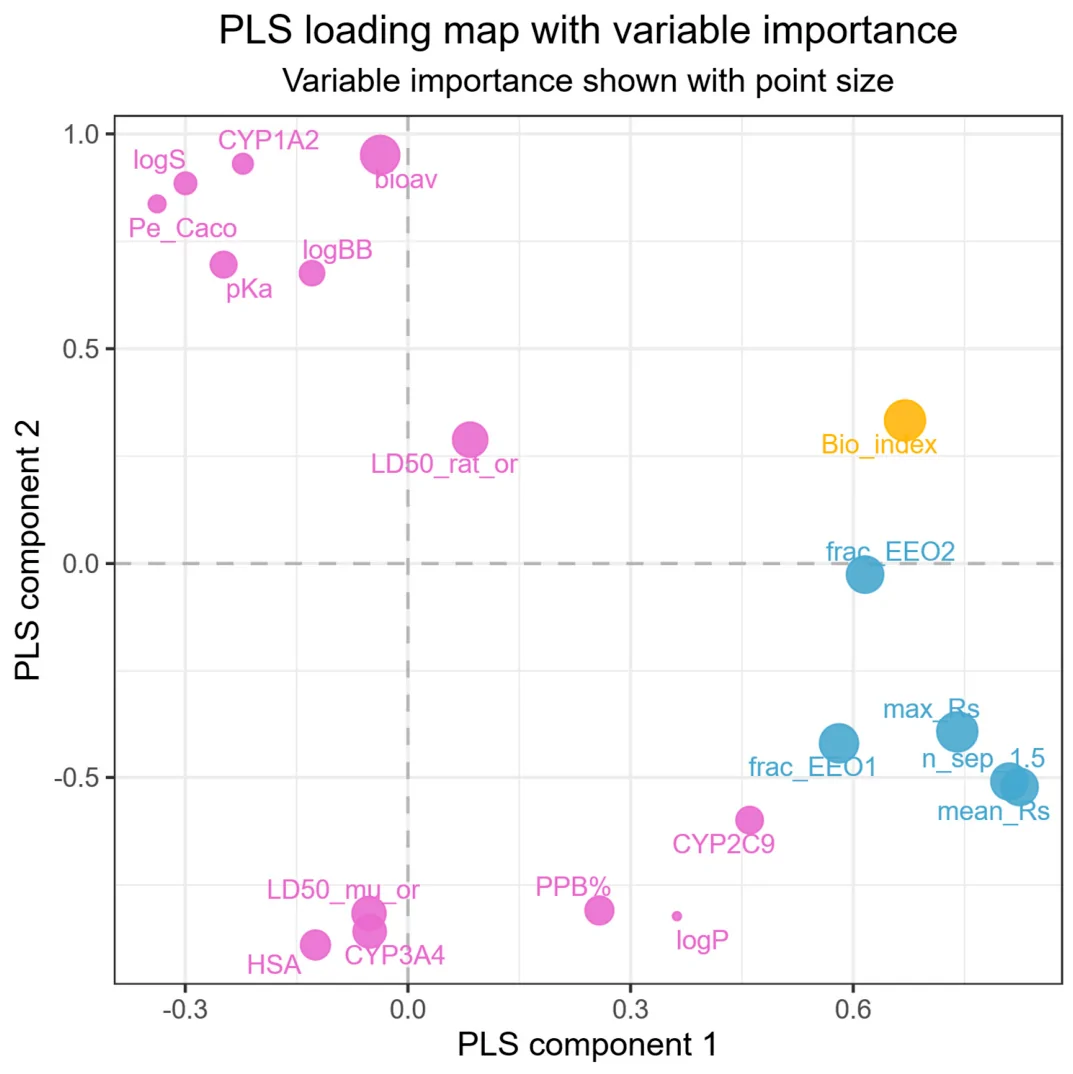
Partial least squares (PLS) loading map linking chromatographic and computational descriptors to the biological index. The plot shows the loadings of predictor variables on the first two PLS components derived from the combined HPLC and Percepta descriptor matrix. Point size reflects the Variable Importance in Projection (VIP) score, highlighting predictors that contribute most strongly to the PLS model.

A Partial Least Squares (PLS) regression model was constructed to examine the relationship between chromatographic parameters, molecular descriptors and the biological outcome, with the biological index serving as the response variable and the combined HPLC and Percepta descriptors as predictors. PLS was selected due to its suitability for situations where the number of predictors significantly exceeds the number of observations and where predictors may exhibit substantial multicollinearity, as it constructs latent components that maximize the covariance between the predictor matrix and the response variable. The response variable, Bio_index, was defined as the normalized difference between the number of assays favoring enantiomer 1 and the number of assays favoring enantiomer 2: Bio_index = (n_E1 − n_E2)/(n_E1 + n_E2). Positive values indicate a predominance of enantiomer 1, negative values indicate a predominance of enantiomer 2, and values close to zero indicate no consistent enantioselective preference. Each PLS component represents a direction in predictor space that captures variance in the predictor variables while maximizing covariance with the response. In the present analysis, no variable selection or penalization methods, such as elastic net or sparse PLS, were applied; therefore, all predictors contributed to the construction of the latent components. The results are summarized using a loading map (Figure 9) of the first two PLS components. In this biplot-like representation (as used for principal component analysis, PCA), the coordinates of each variable correspond to its loadings on the first and second components, indicating how strongly that variable contributes to the respective latent directions. Variables located close together in this space tend to vary similarly across the dataset, while variables positioned in opposite directions indicate opposing contributions to the latent structure. The orientation of variables relative to the direction associated with the response (Bio_index in yellow) reflects whether they contribute positively or negatively to the biological outcome along the extracted components. Alongside the loadings, Variable Importance in Projection (VIP) scores were calculated to quantify the total contribution of each predictor to the PLS model and represented by point size in Figure 9. Variables with larger VIP values have a stronger influence on the latent structure captured by the PLS model. Given the small number of compounds (n = 10) relative to the number of predictors, this PLS analysis was treated as an exploratory descriptive model rather than as a validated predictive model; no external predictive-performance claims are made.

The PLS loading map supported the patterns seen in the correlation analysis. The HPLC-derived variables were positioned closest to Bio_index, indicating that chromatographic behavior carried the strongest information about the direction of biological enantioselectivity. Among these variables, frac_EEO2 was located nearest to Bio_index, suggesting that the frequency of the second elution-order class was particularly informative. Most HPLC variables loaded positively along the first PLS component, whereas the ACD/Percepta descriptors were distributed mainly along the second component, forming a largely orthogonal descriptor-driven axis. Among the descriptors, CYP2C9 was closest to the HPLC variables and Bio_index, while LD50_mu_or, CYP3A4, and HSA clustered together on the negative side of the second component. The opposite descriptor cluster, located on the positive side of the second component, included bioav, logBB, and CYP1A2. Interestingly, logP was not important in PLS and LD50_rat_or showed negligible contribution, in contrast to the stronger loading observed for LD50_mu_or. Overall, the PLS results were consistent with the Spearman correlation heatmaps and suggest that HPLC elution-order metrics, especially EEO-related variables, are associated with biological enantioselectivity within the present dataset, while chromatographic resolution and selected ACD/Percepta descriptors related to CYP liability, mouse oral toxicity, lipophilicity, and blood–brain barrier penetration also contributed to the latent structure.

## 4. Conclusions

The results demonstrate that stereochemistry and substituent pattern jointly influence biological activity in this thiourea series. Target-dependent enantioselectivity was evident in both antiproliferative and antibacterial assays: (S)-5 showed the strongest activity in C6 glioma cells, (R)-4 and (R)-8 were the most relevant HL-60-directed derivatives, and (S)-7 was the most active anti-MRSA analogue. The structure–activity analysis further indicated that aromatic, particularly halogenated, substitution generally favored activity, while the marked difference between the para- and ortho-trifluoromethylphenyl analogues highlighted the importance of substituent position.

From an analytical perspective, all enantiomeric pairs could be resolved under at least one polar-organic HPLC condition. Methanol and Lux Amylose-1 provided the strongest overall separation performance, while enantiomer elution order depended on the combined effects of analyte substitution, selector structure, and mobile phase. AGP-based chromatography was more sensitive than HSA to stereochemical differences for the investigated subset, and molecular docking reproduced most of the observed AGP-related trends. Overall, the integrated biological, chromatographic, and computational approach provides a practical framework for enantiomer-resolved early developability assessment of this thiourea series.

## Figures and Tables

**Table 1:** The structure and numbering of the compounds investigated.

General Structure	R Group	Numbering
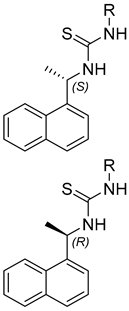	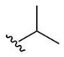	(S)-1
		(R)-1
	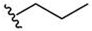	(S)-2
		(R)-2
	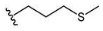	(R)-3
		(S)-3
	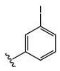	(S)-4
		(R)-4
	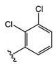	(S)-5
		(R)-5
	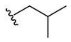	(S)-6
		(R)-6
	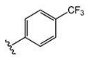	(S)-7
		(R)-7
	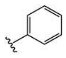	(S)-8
		(R)-8
	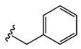	(S)-9
		(R)-9
	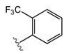	(S)-10
		(R)-10

**Table 2:** Retention factors and HPLC-estimated apparent HSA and AGP interaction values determined on protein-based columns using 10 mM phosphate buffer/2-propanol (90:10, *v*/*v*; pH 7.0) as the mobile phase. AGP-1 and AGP-2 denote the first- and second-eluting peaks, respectively; where no enantiorecognition was observed, a single AGP value is reported.

Compound	k′_HSA_	App. HSA(%)	k′_AGP-1_	k′_AGP-2_	App. AGP-1 (%)	App. AGP-2 (%)
**1**	8.37	89.33	10.87	10.87	91.58	
**2**	9.98	90.89	11.28	13.50	91.86	93.1
**3**	15.54	93.95	14.02	17.96	93.34	94.73
**6**	14.72	93.63	19.91	19.91	95.22	
**8**	33.01	97.05	28.92	32.67	96.65	97.03
**10**	39.59	97.53	36.78	50.28	97.35	98.05

**Table 3:** Glide docking scores (in kcal/mol) between the enantiomers of the studied thiourea derivatives (2, 3, 8, and 10) and human α(1)-acid glycoprotein (AGP) structures (3APW and 3KQ0) employed to model the AGP protein-based CSP.

Ligand	PDB ID: 3APW	PDB ID: 3KQ0
(R)-2	−6.3	−5.7
(S)-2	−5.8	−5.6
(R)-3	−6.3	−6.0
(S)-3	−6.3	−5.9
(R)-8	−5.8	−5.5
(S)-8	−5.5	−4.7
(R)-10	−6.9	−7.8
(S)-10	−6.8	−7.7

**Table 4:** Antiproliferative activity of the investigated thiourea enantiomers and the reference compound on C6 glioma and HL-60 leukemia cells, with NIH 3T3 fibroblasts as non-malignant controls, expressed as IC_50_ values (nM). (–, no IC_50_ value could be determined within the investigated concentration range.)

Compound	3T3 IC_50_ (nM)	C6 IC_50_ (nM)	HL-60 IC_50_ (nM)
(S)-1	279.6	–	–
(R)-1	37.23	–	–
(S)-2	350.8	–	–
(R)-2	149.5	–	–
(S)-3	–	–	–
(R)-3	925.5	–	–
(S)-4	507.4	224.3	–
(R)-4	346.3	–	84.85
(S)-5	–	45.81	–
(R)-5	–	611.2	157.1
(S)-6	211.7	–	–
(R)-6	718.7	–	–
(S)-7	–	199.2	–
(R)-7	–	272.3	493.7
(S)-8	–	–	359.1
(R)-8	2474	2186	271.1
(S)-9	2556	–	1052
(R)-9	–	–	–
(S)-10	210.3	–	–
(R)-10	50.8	2620	–
10a (reference) [28]	332.5	57.97	93.41

**Table 5:** Antibacterial activity of the investigated thiourea enantiomers and vancomycin reference compound against methicillin-sensitive and methicillin-resistant *Staphylococcus aureus*, expressed as MIC values (μM). –, no measurable antibacterial activity within the investigated concentration range.

Compound	*S. aureus* MSSA MIC (μM)	*S. aureus* MRSA MIC (μM)
(S)-1	–	–
(R)-1	–	–
(S)-2	100	–
(R)-2	–	–
(S)-3	–	–
(R)-3	–	–
(S)-4	25	25
(R)-4	12.5	12.5
(S)-5	12.5	25
(R)-5	12.5	12.5
(S)-6	–	–
(R)-6	–	–
(S)-7	6.25	3.125
(R)-7	6.25	25
(S)-8	50	–
(R)-8	–	–
(S)-9	100	–
(R)-9	–	–
(S)-10	50	–
(R)-10	–	–
Vancomycin (control)	0.90	1.04

**Table 6:** Docking scores (kcal/mol) of the investigated thiourea enantiomers against DNA gyrase, penicillin-binding protein, and isoleucyl-tRNA synthetase (PDB IDs: 2XCQ, 1MWT, and 1JZS, respectively). More negative docking scores indicate more favorable predicted binding interactions.

Enantiomer	DNA Gyrase	Penicillin-Binding Protein	Isoleucyl-tRNA Synthetase
	2XCQ	1MWT	1JZS
(R)-1	−3.7	−4.1	−5.3
(S)-1	−3.2	−4.0	−5.3
(R)-2	−3.1	−3.5	−5.0
(S)-2	−3.2	−3.4	−5.0
(R)-3	−3.7	−3.8	−5.1
(S)-3	−2.8	−3.6	−5.1
(R)-4	−1.6	−4.5	−5.8
(S)-4	−2.3	−4.5	−5.8
(R)-5	−3.2	−5.2	−6.2
(S)-5	−2.5	−5.2	−6.2
(R)-6	−3.2	−4.0	−5.2
(S)-6	−3.2	−3.9	−5.2
(R)-7	−3.2	−4.5	−5.8
(S)-7	−2.6	−4.4	−5.7
(R)-8	−2.9	−3.3	−4.5
(S)-8	−3.0	−3.2	−4.2
(R)-9	−3.4	−4.3	−5.5
(S)-9	−3.2	−4.2	−5.4
(R)-10	−2.9	−5.1	−6.0
(S)-10	−3.0	−5.1	−6.0

## Data Availability

The original contributions presented in this study are included in the article and Supplementary Materials. Additional raw data are available from the corresponding author upon reasonable request.

## Supplementary Materials

The following supporting information can be downloaded at: https://www.mdpi.com/article/10.3390/pharmaceutics18091129/s1. Supporting Information S1. Detailed spectroscopic characterization of newly synthesized thioureas, Supplementary Table S1. Enantioseparation results obtained on amylose-type stationary phases using various eluents; Supplementary Table S2. Enantioseparation results obtained on cellulose-based stationary phases using various eluents; Supporting Information S2. Analysis-Time Distribution across Eluents and Stationary Phases; Supplementary Figure S1. Analysis-time overview across eluents and stationary phases in polar organic mode; Supporting Information S3. Enantiomer Elution-Order Reversal; Supplementary Figure S2. Heatmap of elution-order bias across eluents and chiral stationary phases; Supplementary Figure S3. Reversal of EEO caused by a different selector backbone in the case of compound 10 in a neat 2-propanol eluent, between an Amylose-1 CSP (left) and a Cellulose-1 CSP (right); Supplementary Figure S4. Reversal of EEO caused by a different substituent in the case of compound 8 in a neat 2-propanol eluent, between a Cellulose-1 CSP (left), and a Cellulose-4 CSP (right); Supplementary Figure S5. Chromatograms of compound 4 in methanol (left), acetonitrile (middle), and 2-propanol (right) on Lux Cellulose-1; Supplementary Figure S6. Chromatograms of compound 5 in methanol (left), acetonitrile (middle), and 2-propanol (right) on Lux Amylose-1; Supplementary Table S3. Predicted physicochemical, ADME and toxicity-related parameters of the investigated thiourea derivatives.
