# Supplementary material for: Enantiomer-Resolved Biological Profiling and Chiral Developability Assessment of Novel Naphthylethyl Thioureas
Source: Pharmaceutics. 2026 Sep 8;18(9):1129. doi: 10.3390/pharmaceutics18091129 (PMC13610919; doi:10.3390/pharmaceutics18091129)

SB133.A.1H.CDCl3
SB133A
n izopropil n' naftil tiourea
CDCl3
2024.01.09.

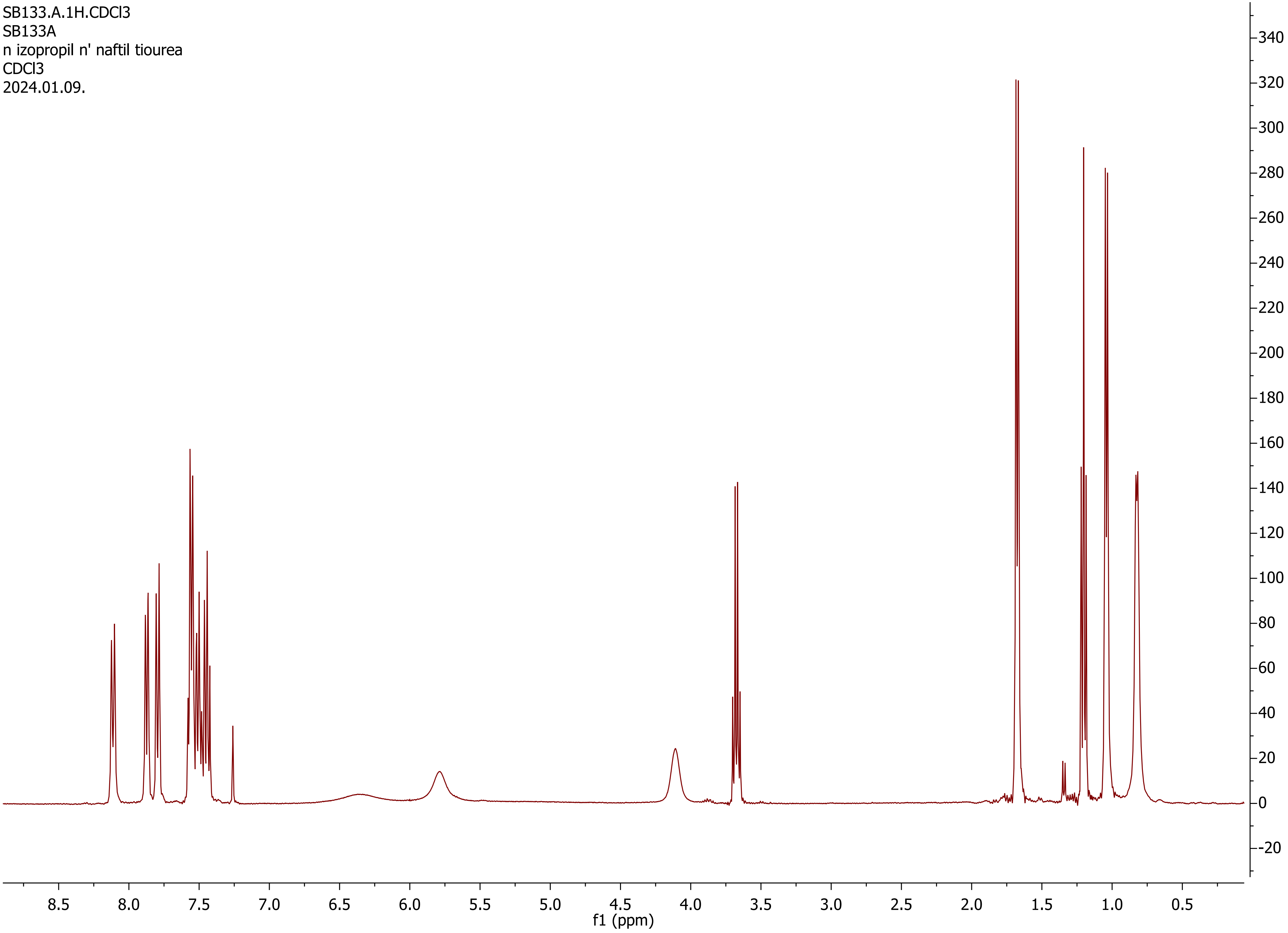

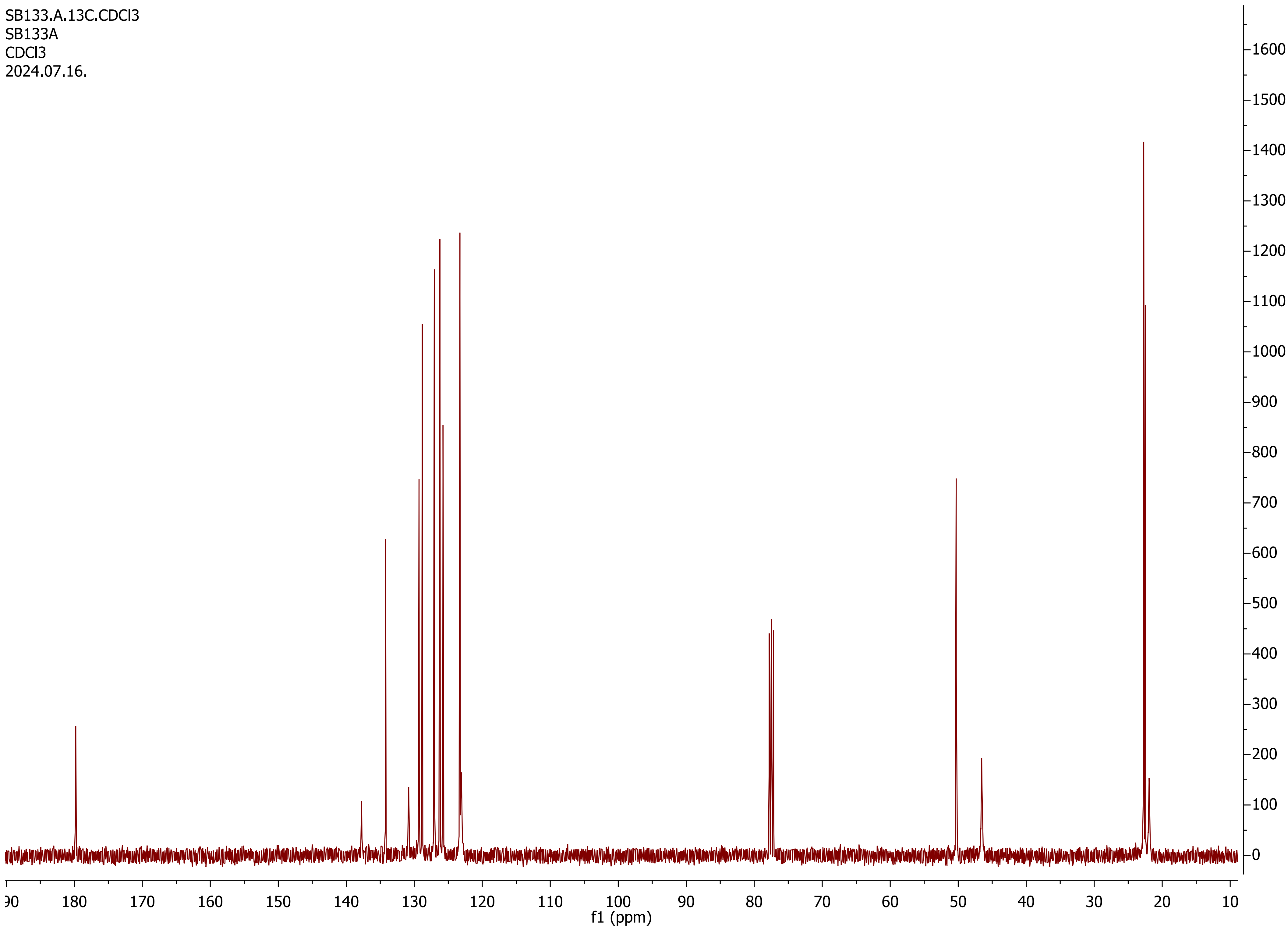
SB133.A.13C.CDCl3
SB133A
CDCl3
2024.07.16.
f1 (ppm)

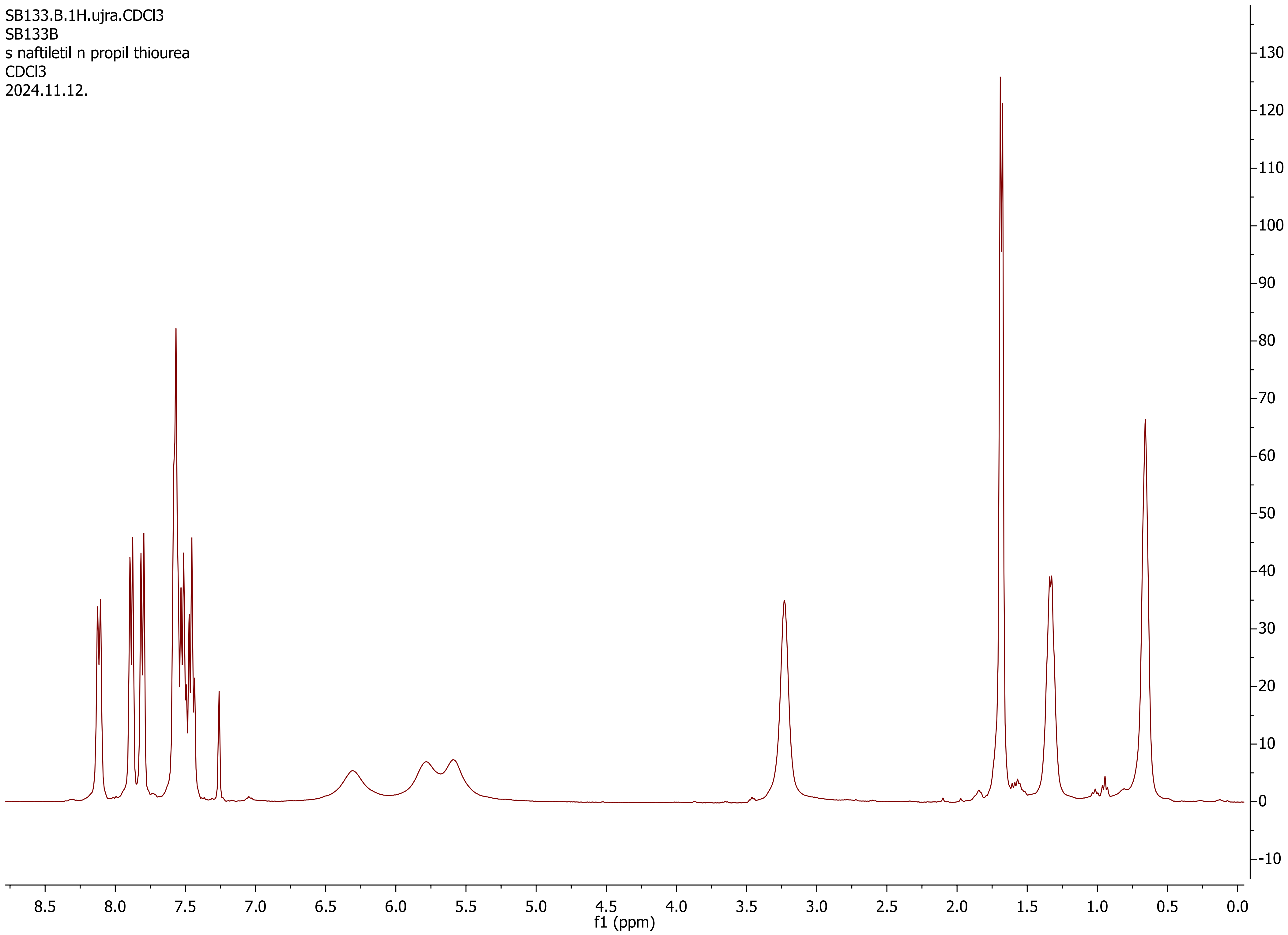
SB133.B.1H.ujra.CDCl3
SB133B
s naftiletil n propil thiourea
CDCl3
2024.11.12.
f1 (ppm)

SB133.B.13C.ujra.CDCl3
SB133B
s naftiletil n propil thiourea
CDCl3
2024.11.12.

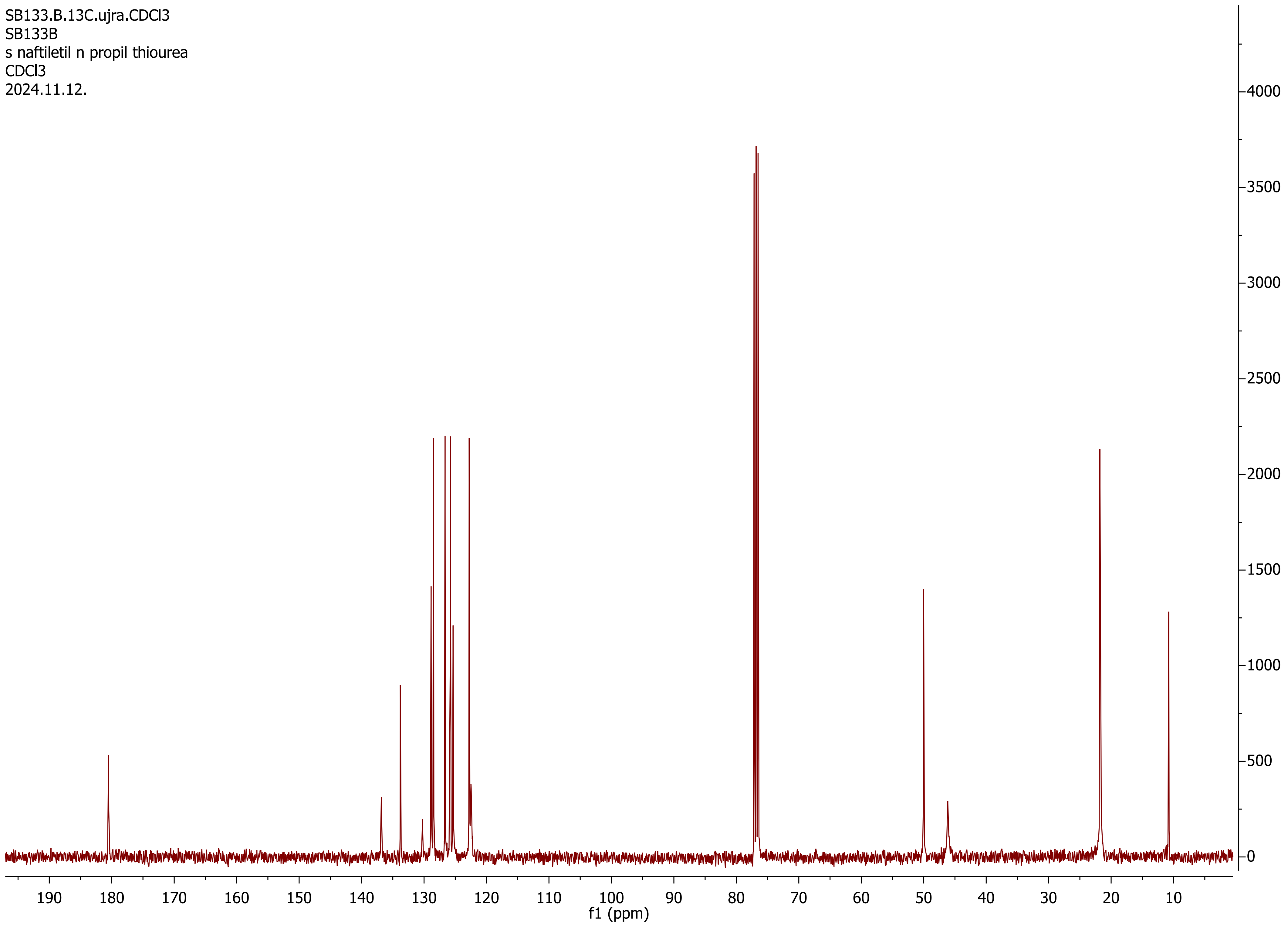

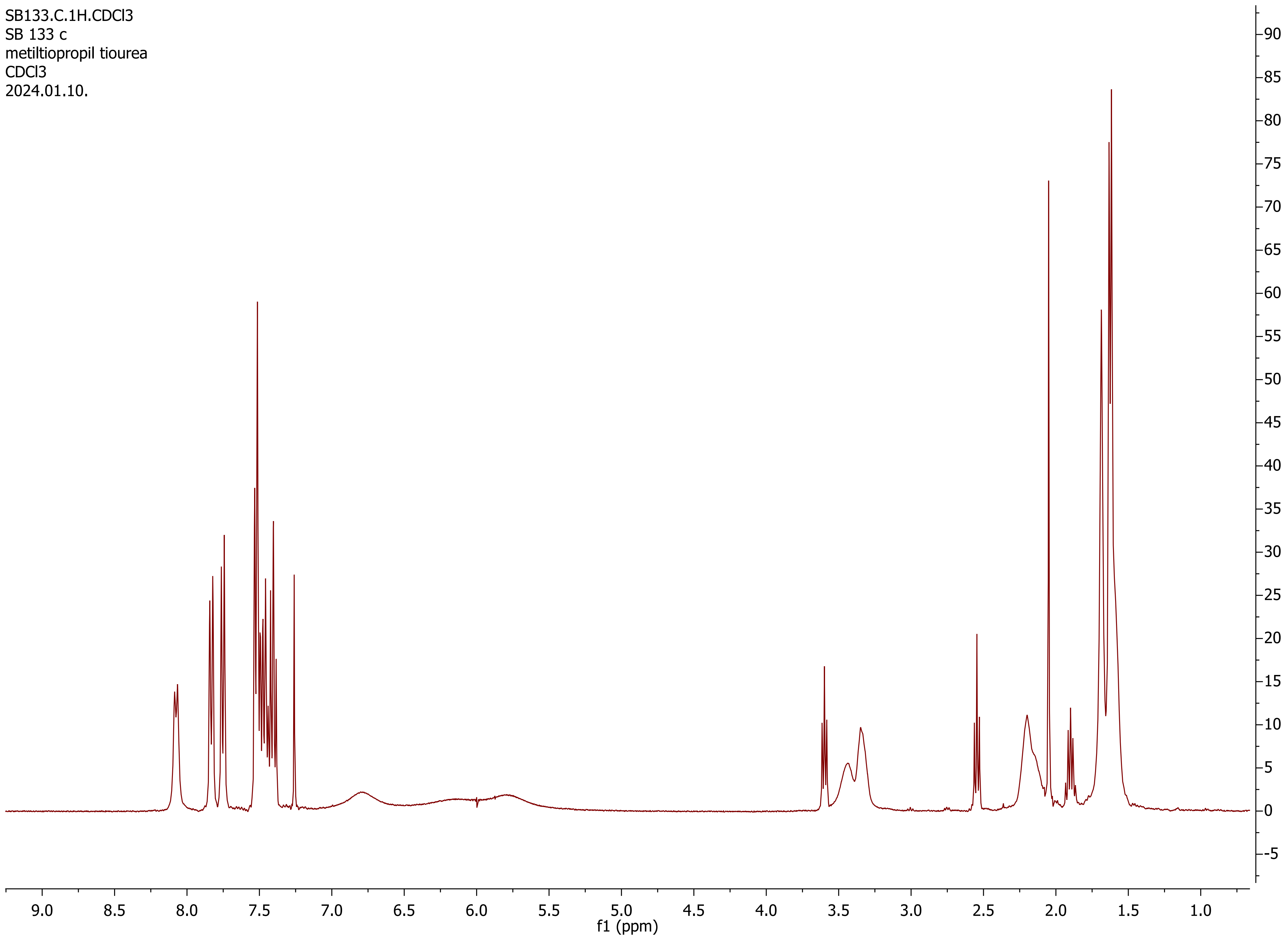
SB133.C.1H.CDCl3
SB 133 c
metiltiopropil tiourea
CDCl3
2024.01.10.
f1 (ppm)

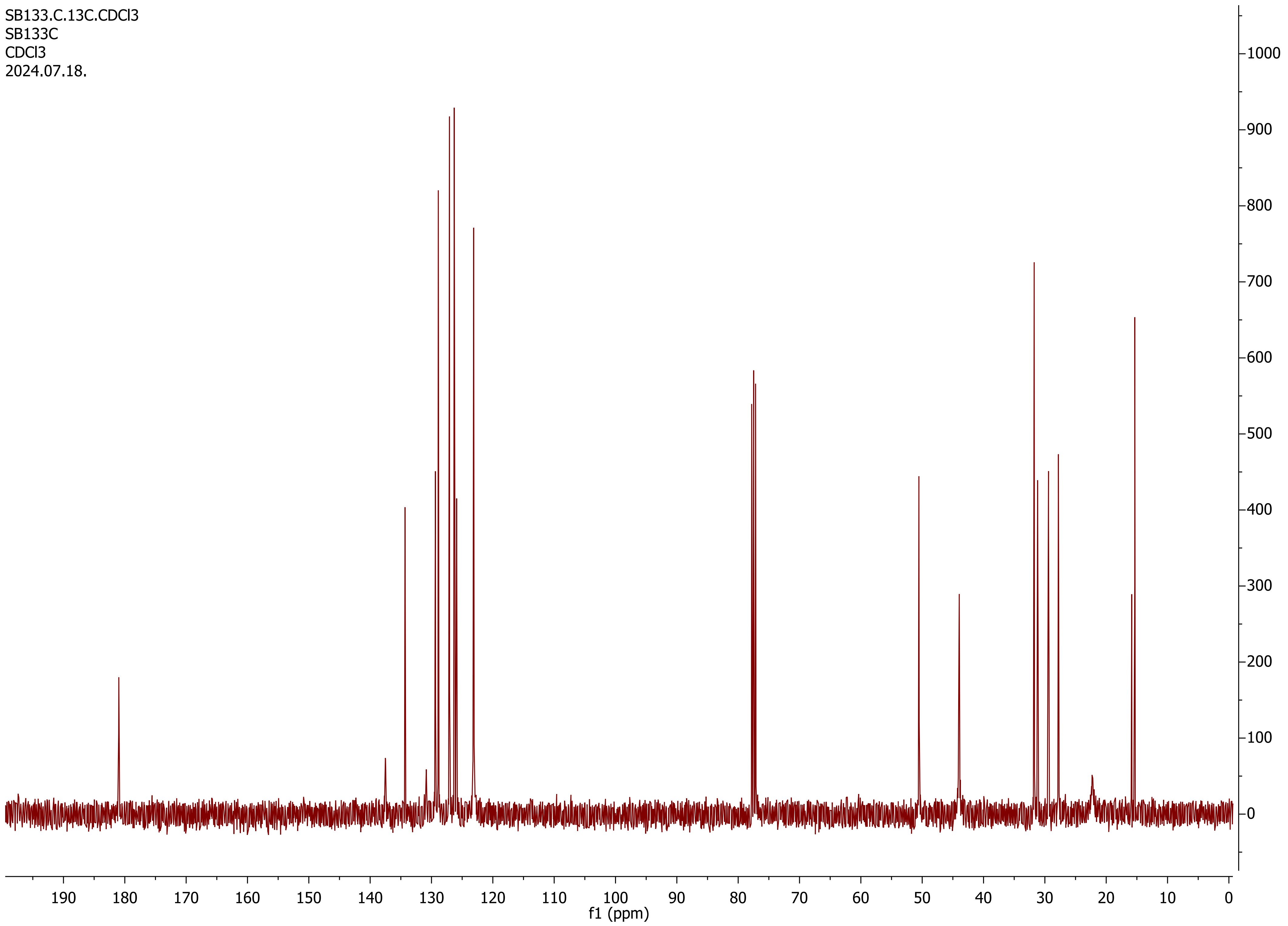
SB133.C.13C.CDCl3
SB133C
CDCl3
2024.07.18.
f1 (ppm)

SB133.F.1H.CDCl3
SB 133 f
vmelyik trifluorometil tiourea
CDCl3
2024.01.10.

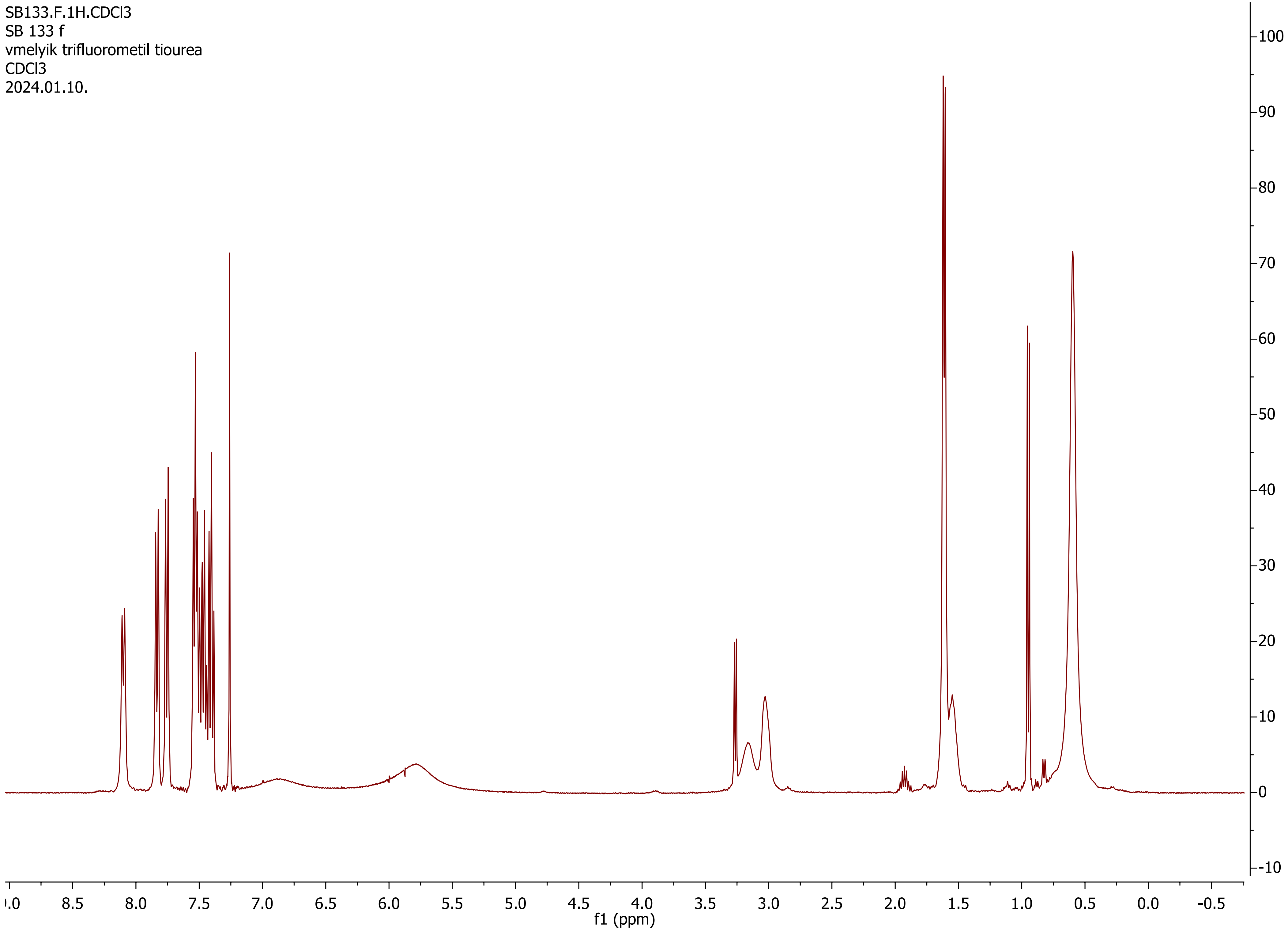

SB133.F.13C.CDCl3
SB133F
CDCl3
2024.07.18.

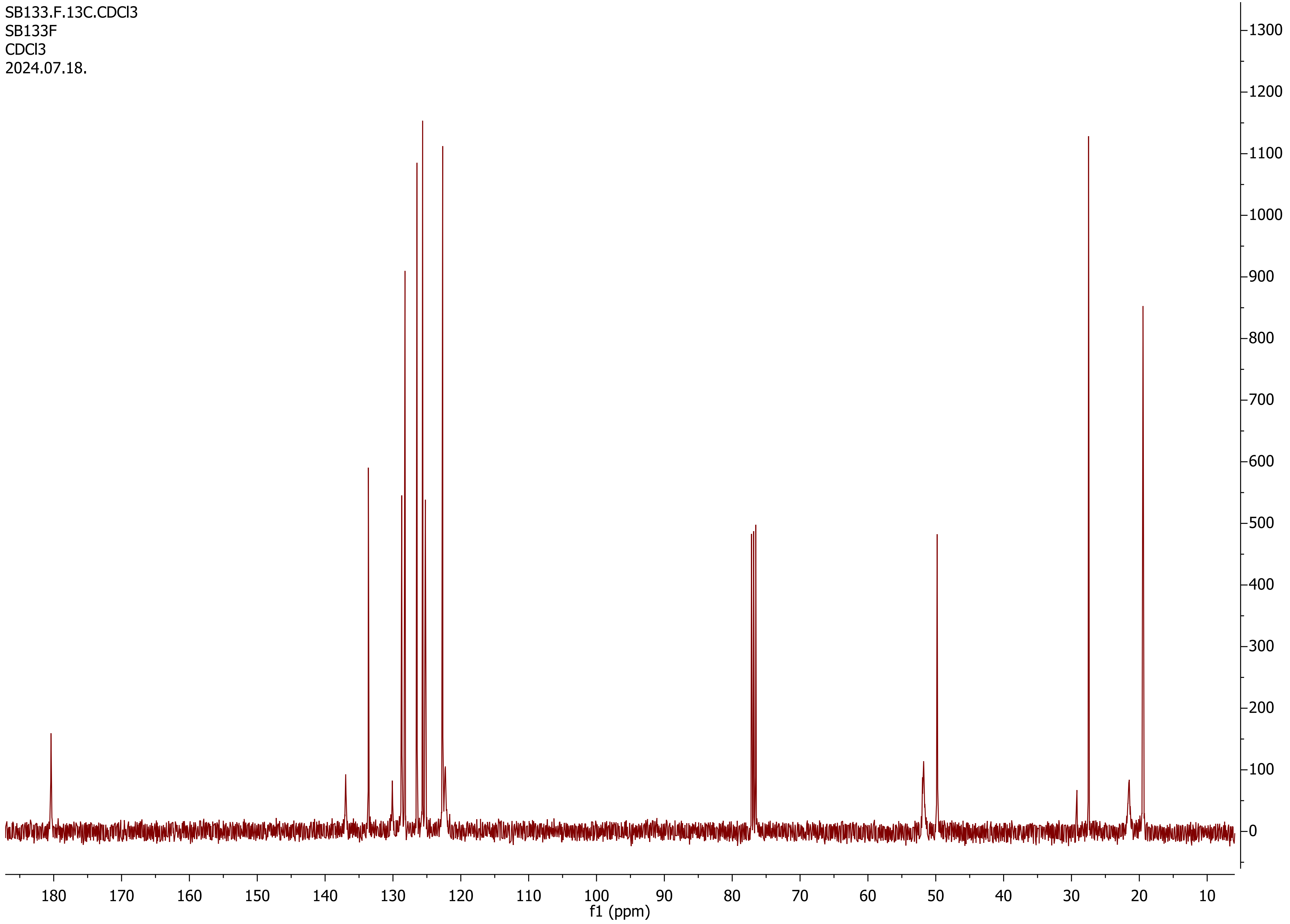

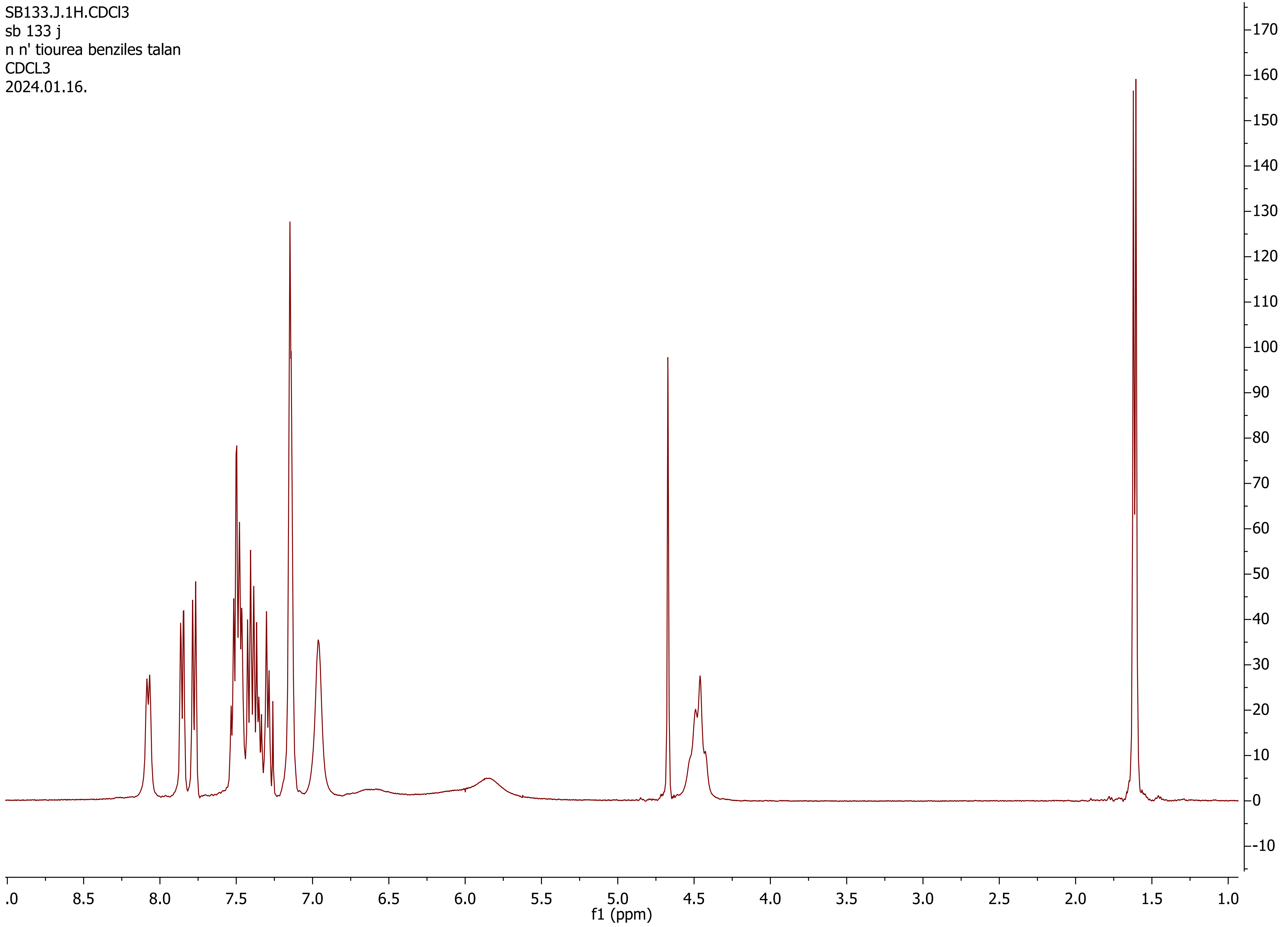
SB133.J.1H.CDCl3
sb 133 j
n n' tiourea benziles talan
CDCL3
2024.01.16.
f1 (ppm)

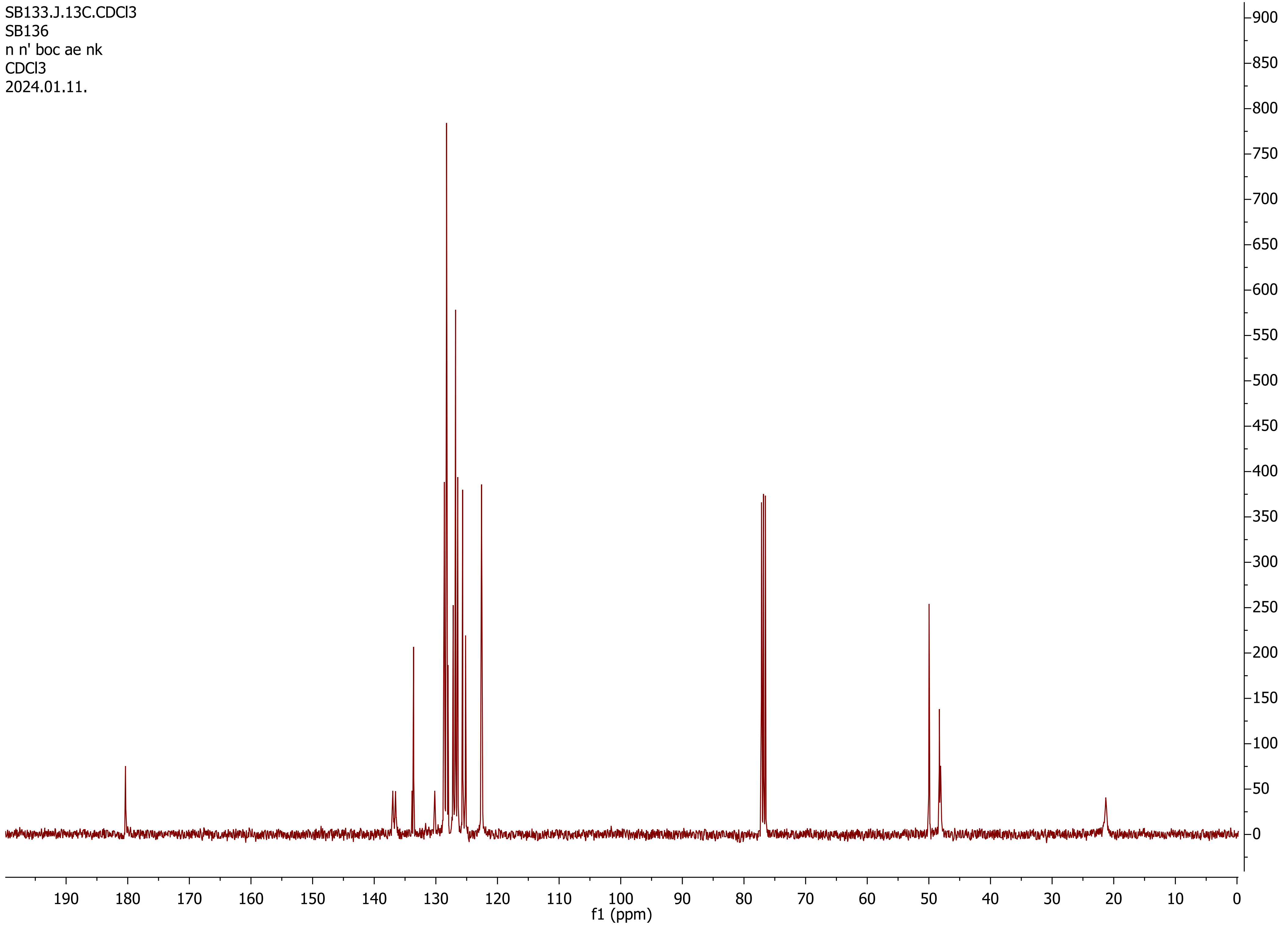

SB133.J.13C.CDCl3
SB136
n n' boc ae nk
CDCl3
2024.01.11.
f1 (ppm)

SB133.L.13C.CDCl3
SB133 l
nn tiourea
CDCl3
2024.01.16.

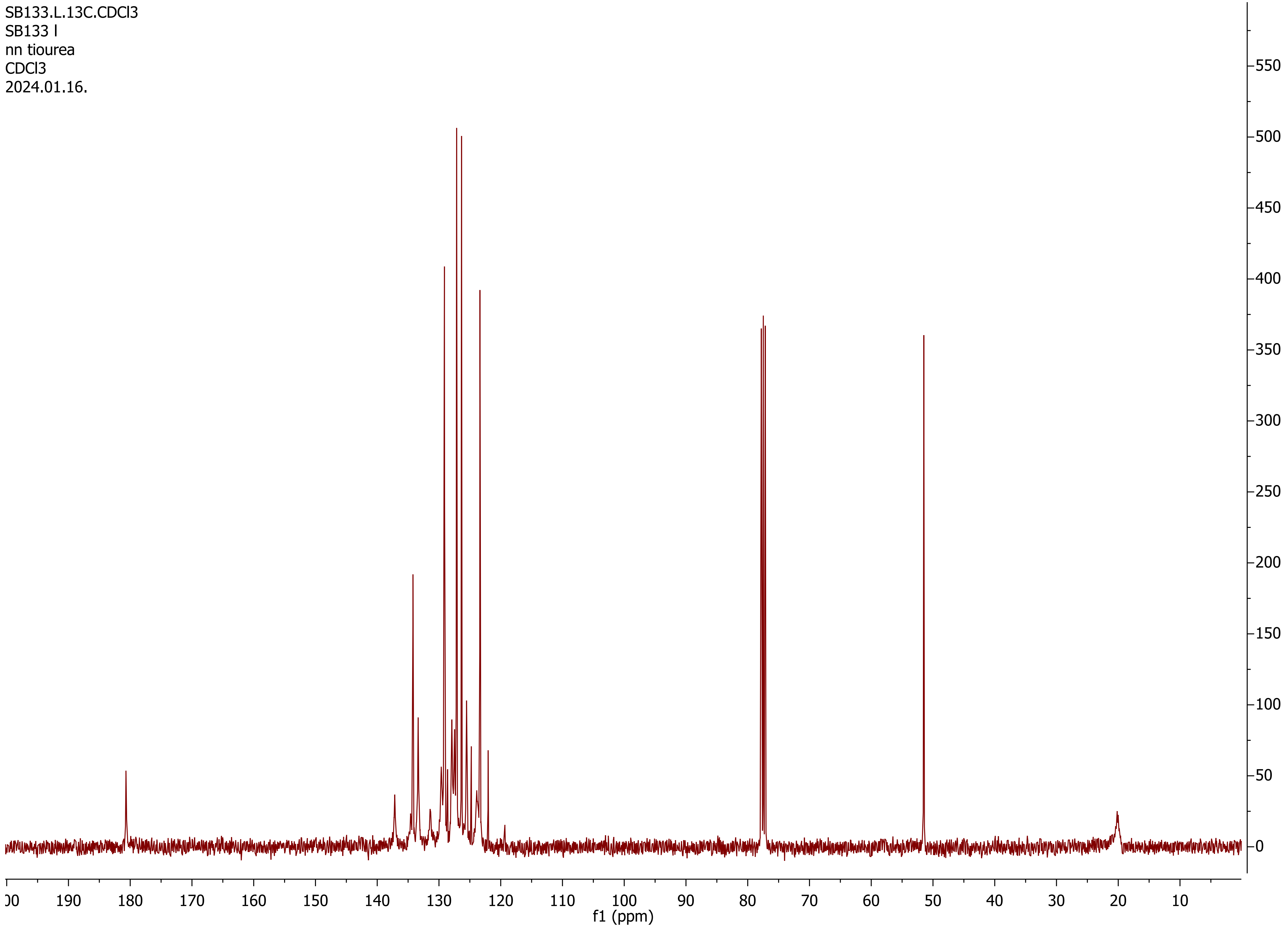

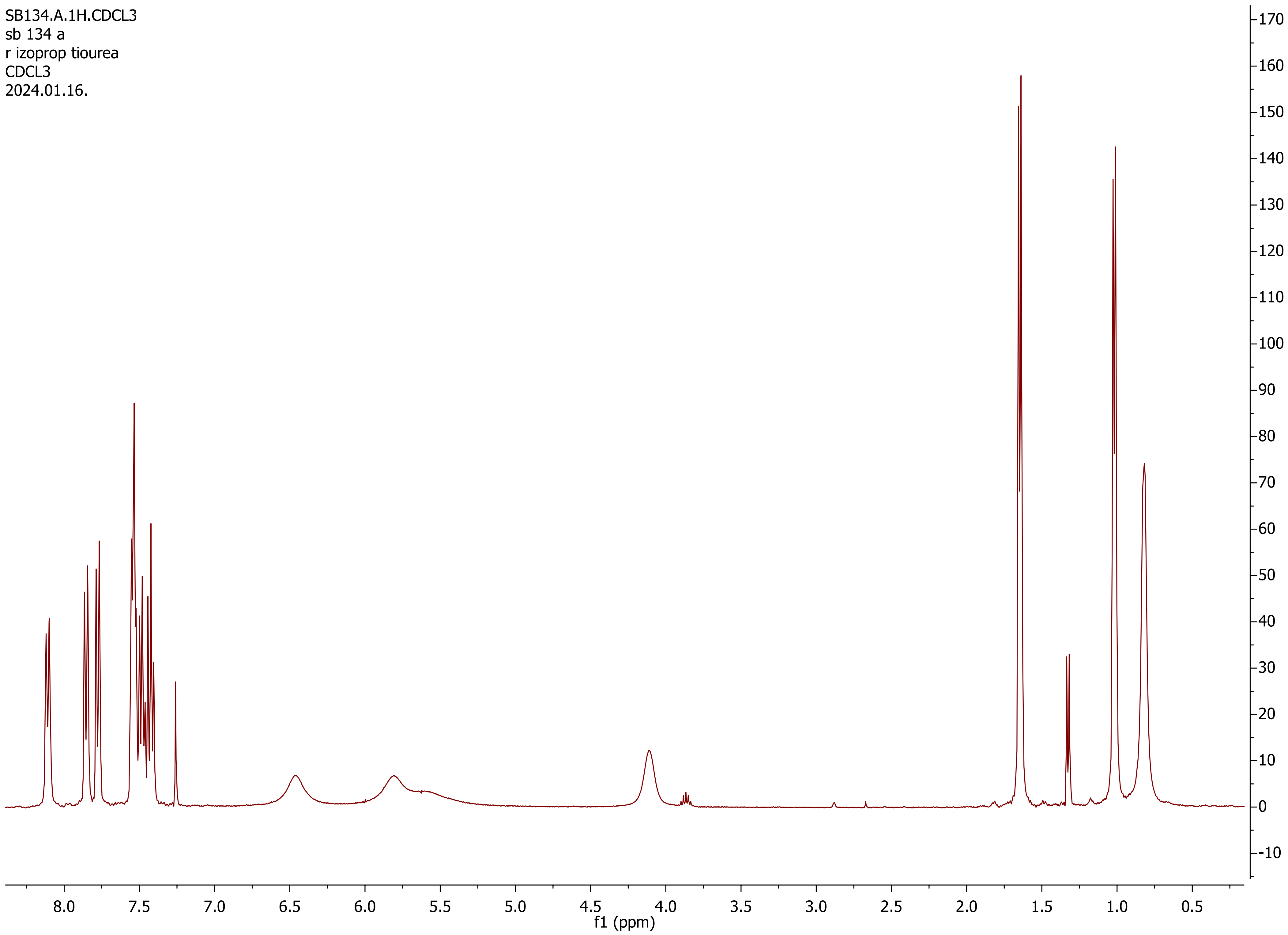
SB134.A.1H.CDCL3
sb 134 a
r izoprop tiourea
CDCL3
2024.01.16.
f1 (ppm)

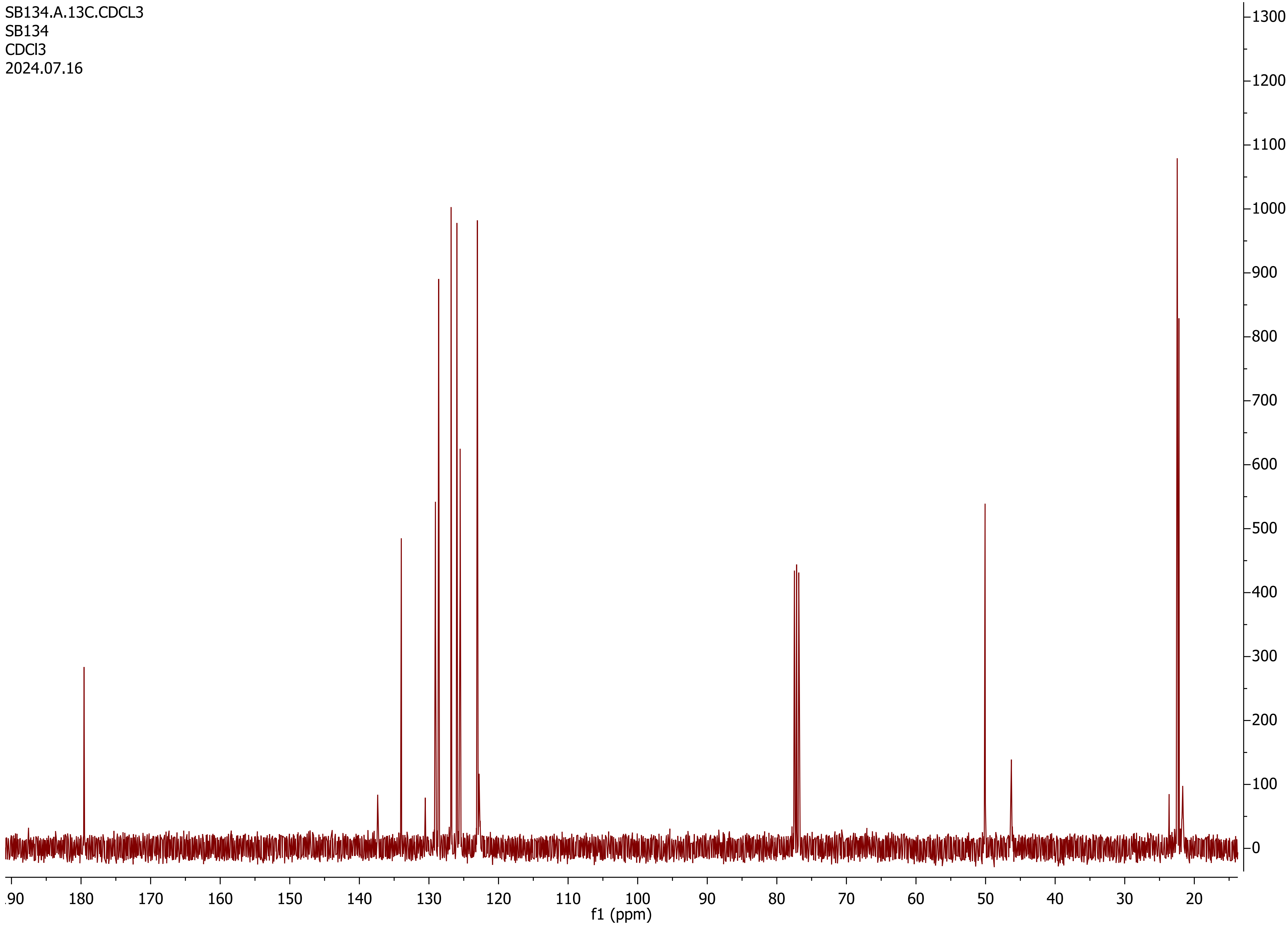
SB134.A.13C.CDCL3
SB134
CDCl3
2024.07.16
f1 (ppm)

SB134.B.1H.CDCL3
sb 134 b
r nprop tiourea
CDCL3
2024.01.17.

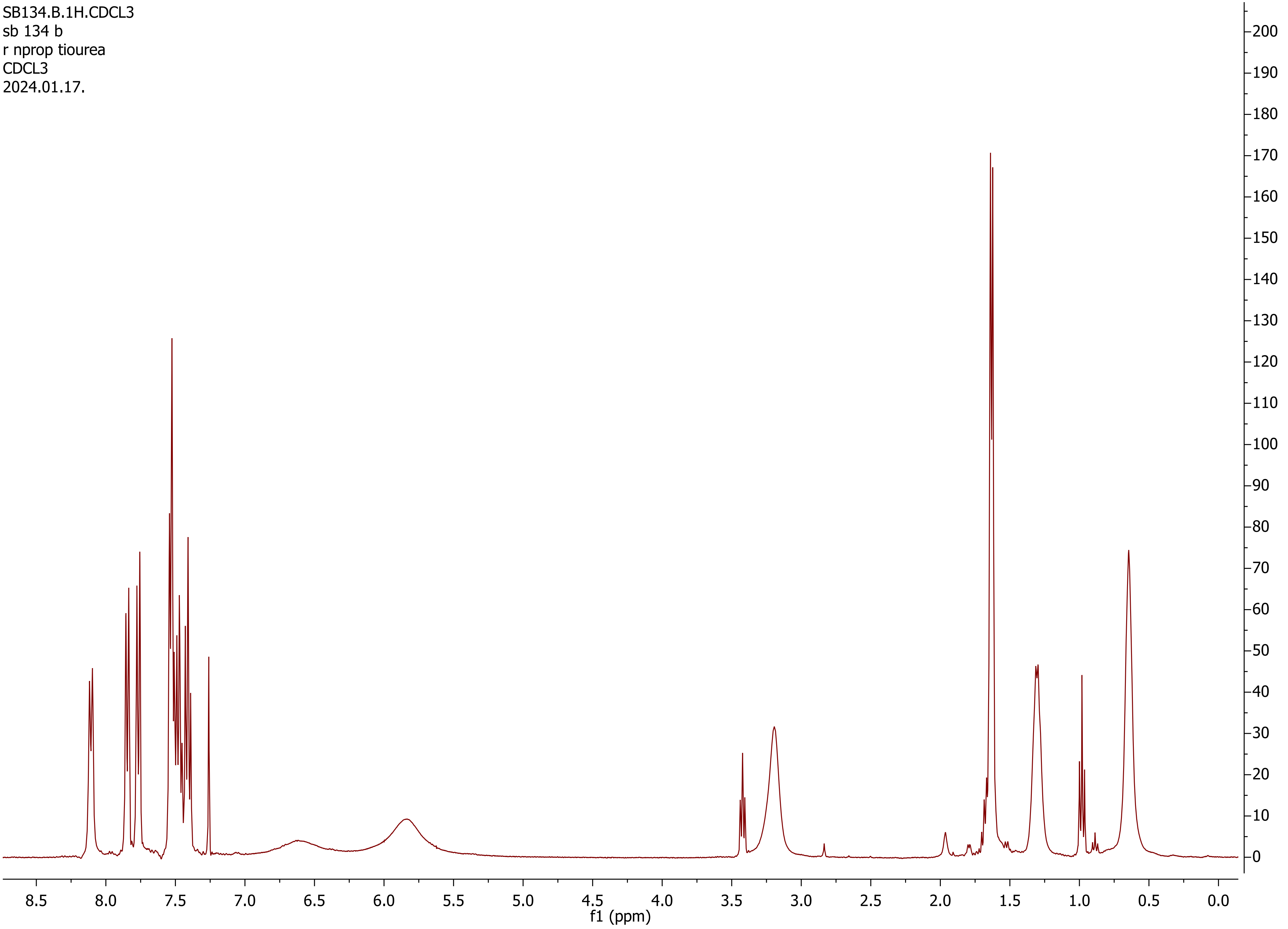

SB134.B.13C.ujra.CDCL3
SB134B
r naftiletil n propil thiourea
CDCl3
2024.11.12.

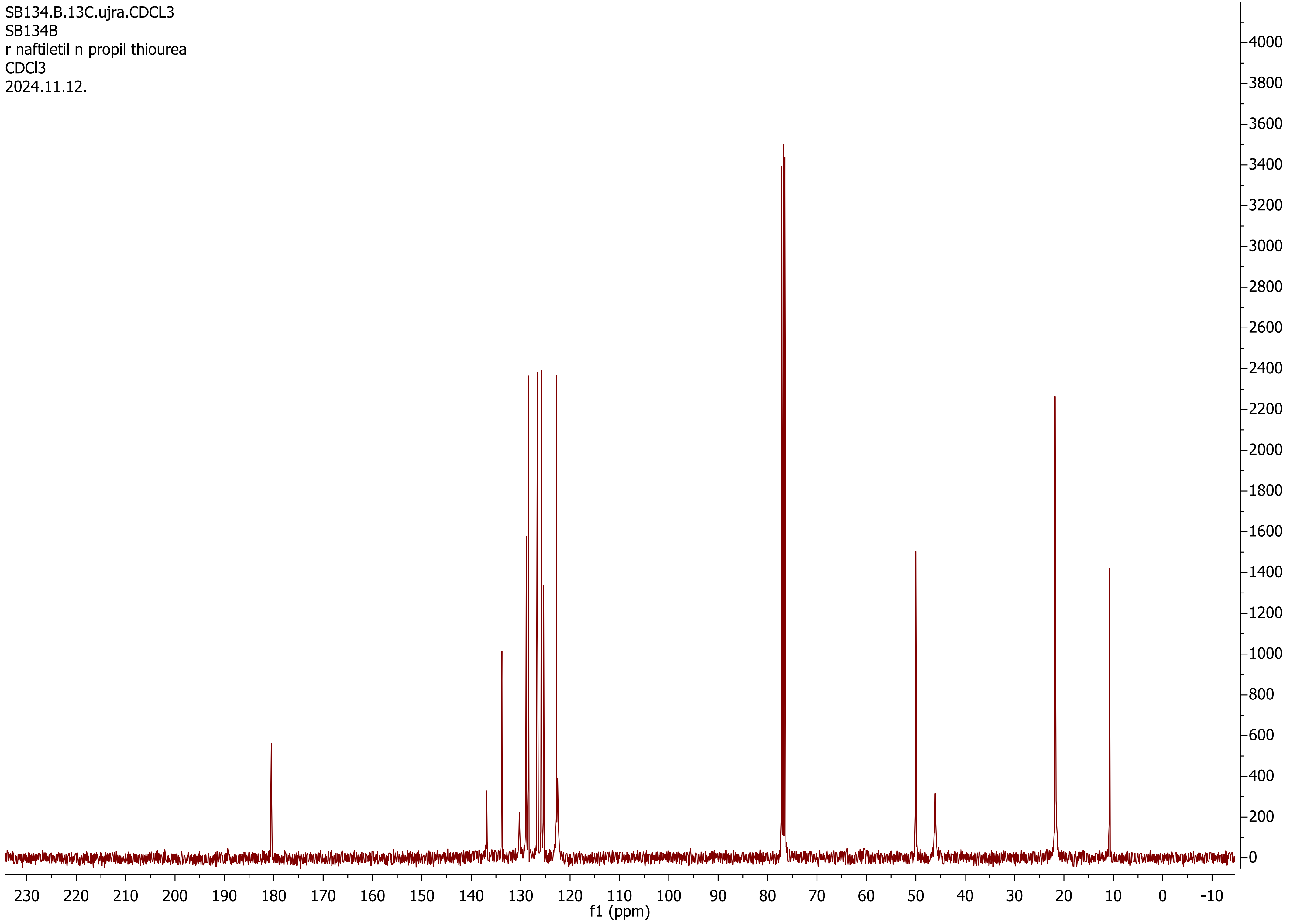

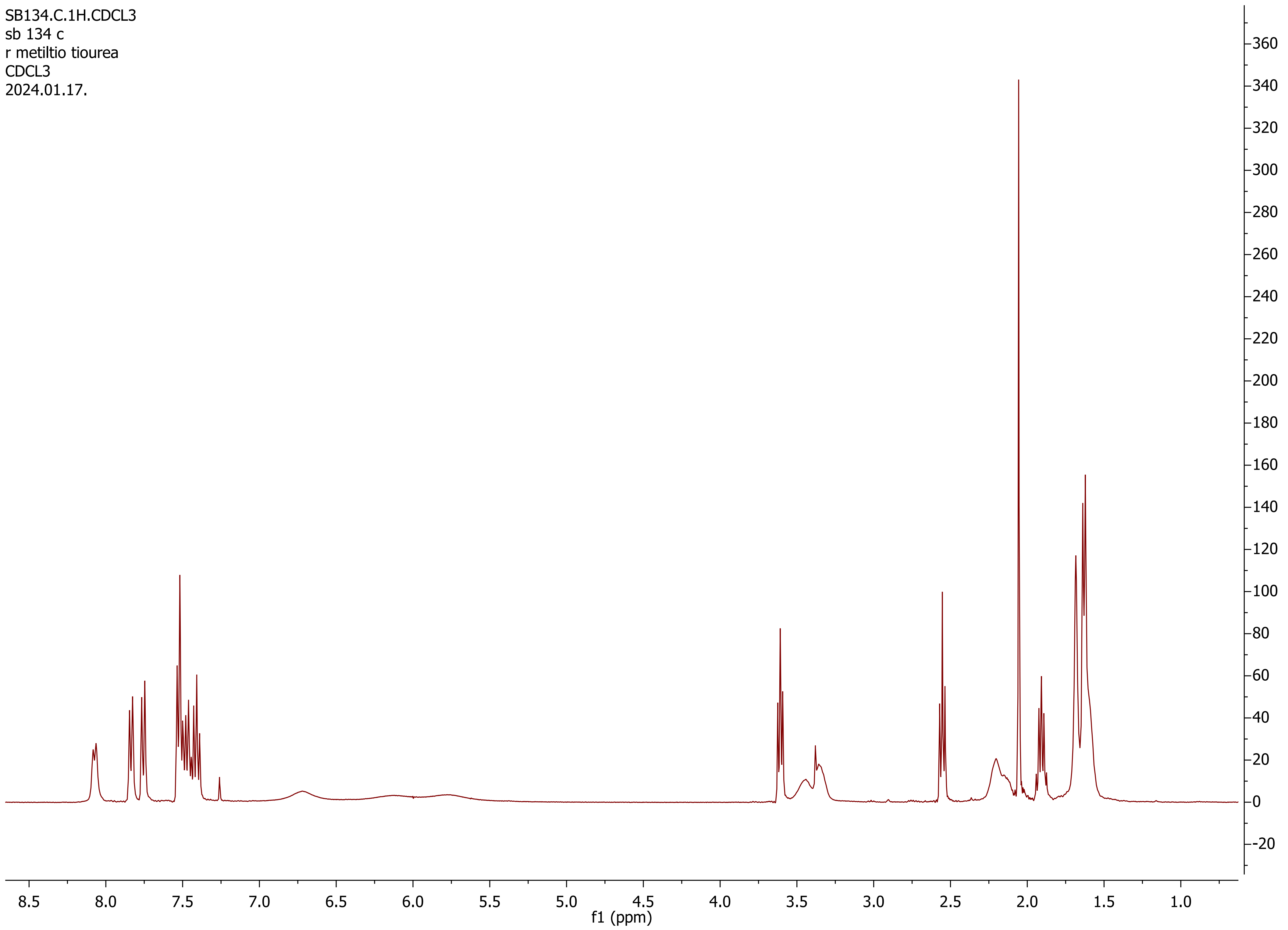
SB134.C.1H.CDCL3
sb 134 c
r metiltio tiourea
CDCL3
2024.01.17.
f1 (ppm)

SB134.C.13C.CDCL3
SB134C
CDCl3
2024.07.16.

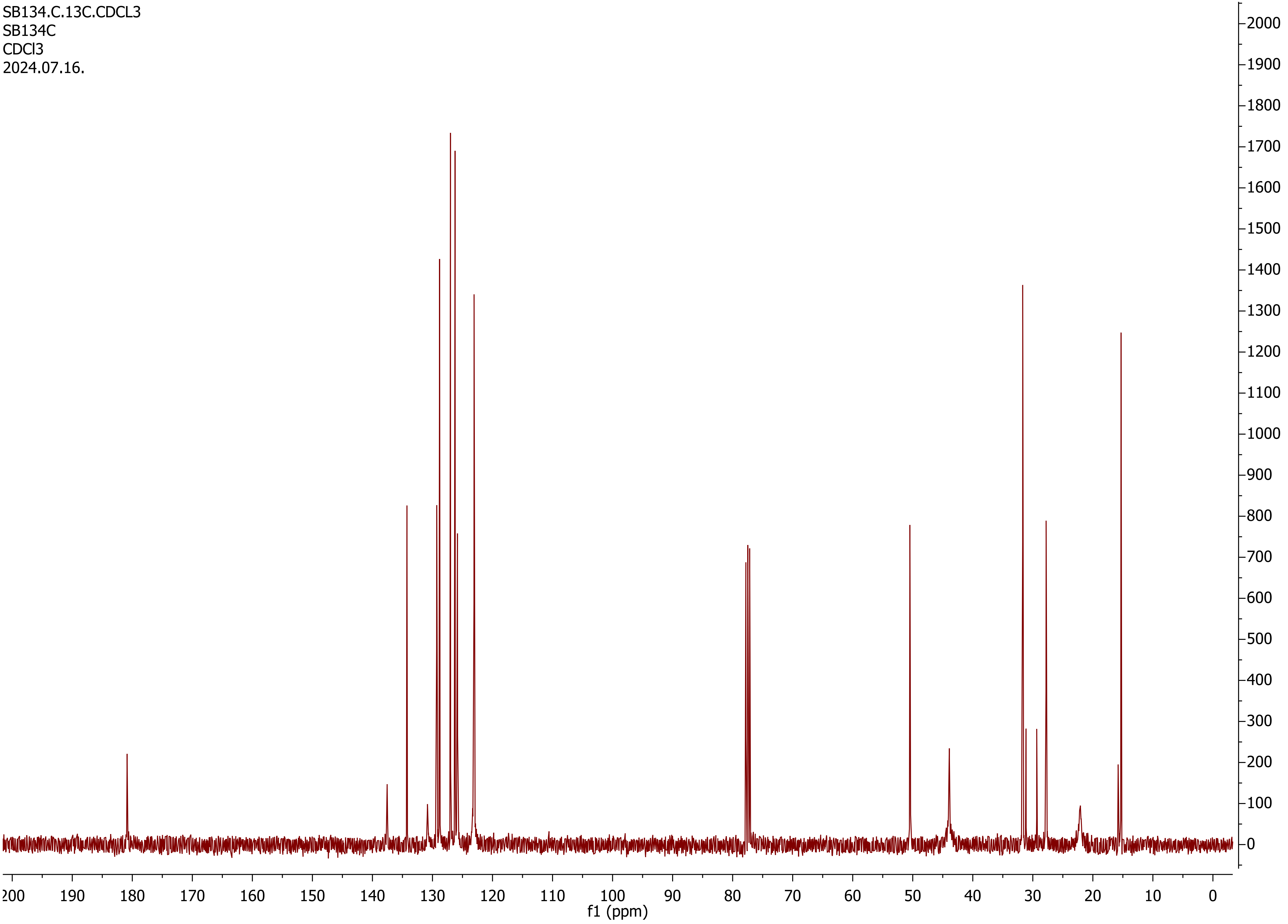

SB134.F.1H.CDCL3
sb 134 f
r izobutil tiourea
CDCL3
2024.01.17.

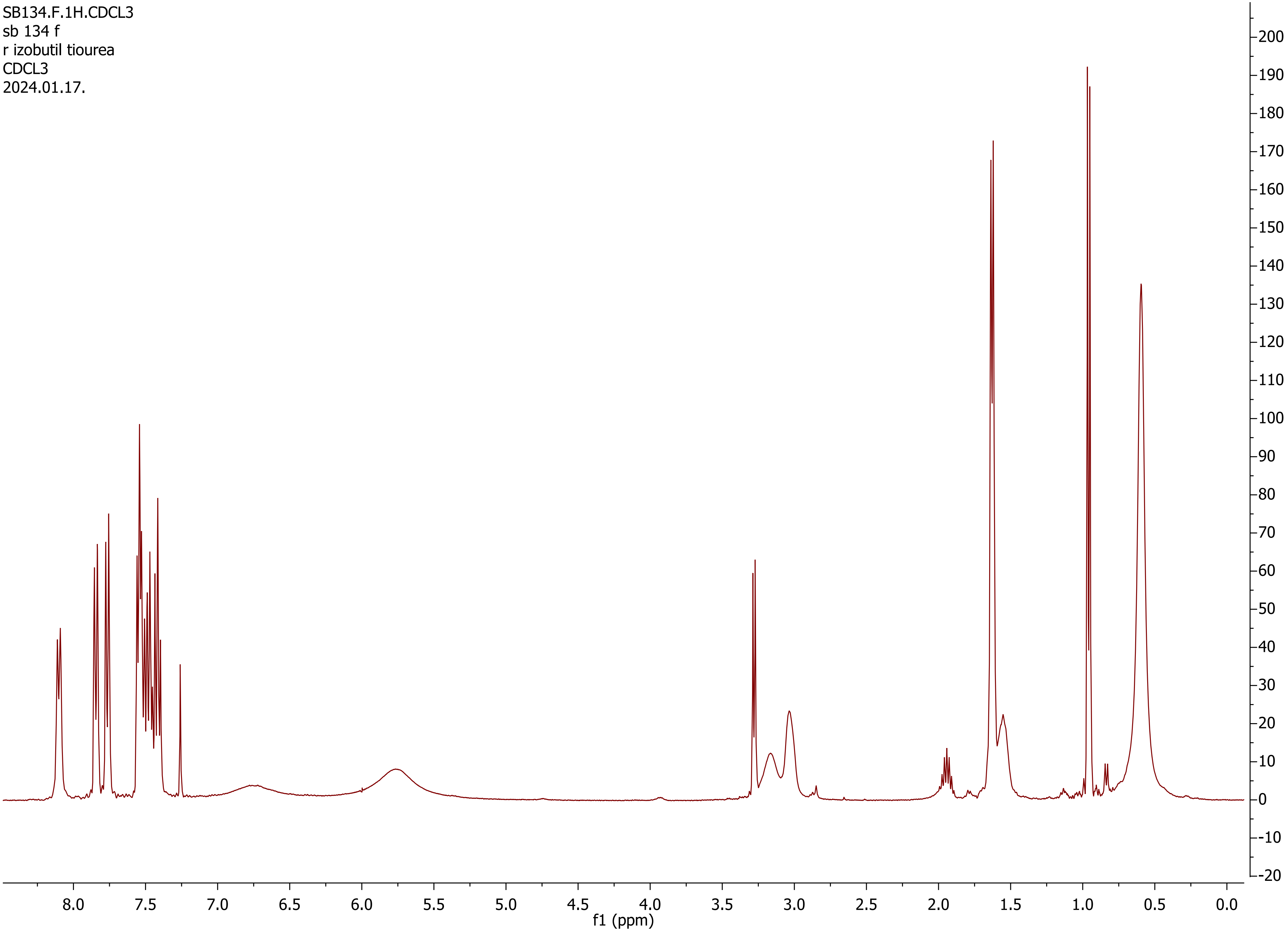

SB134.F.13C.CDCL3
SB134F
CDCl3
2024.07.16.

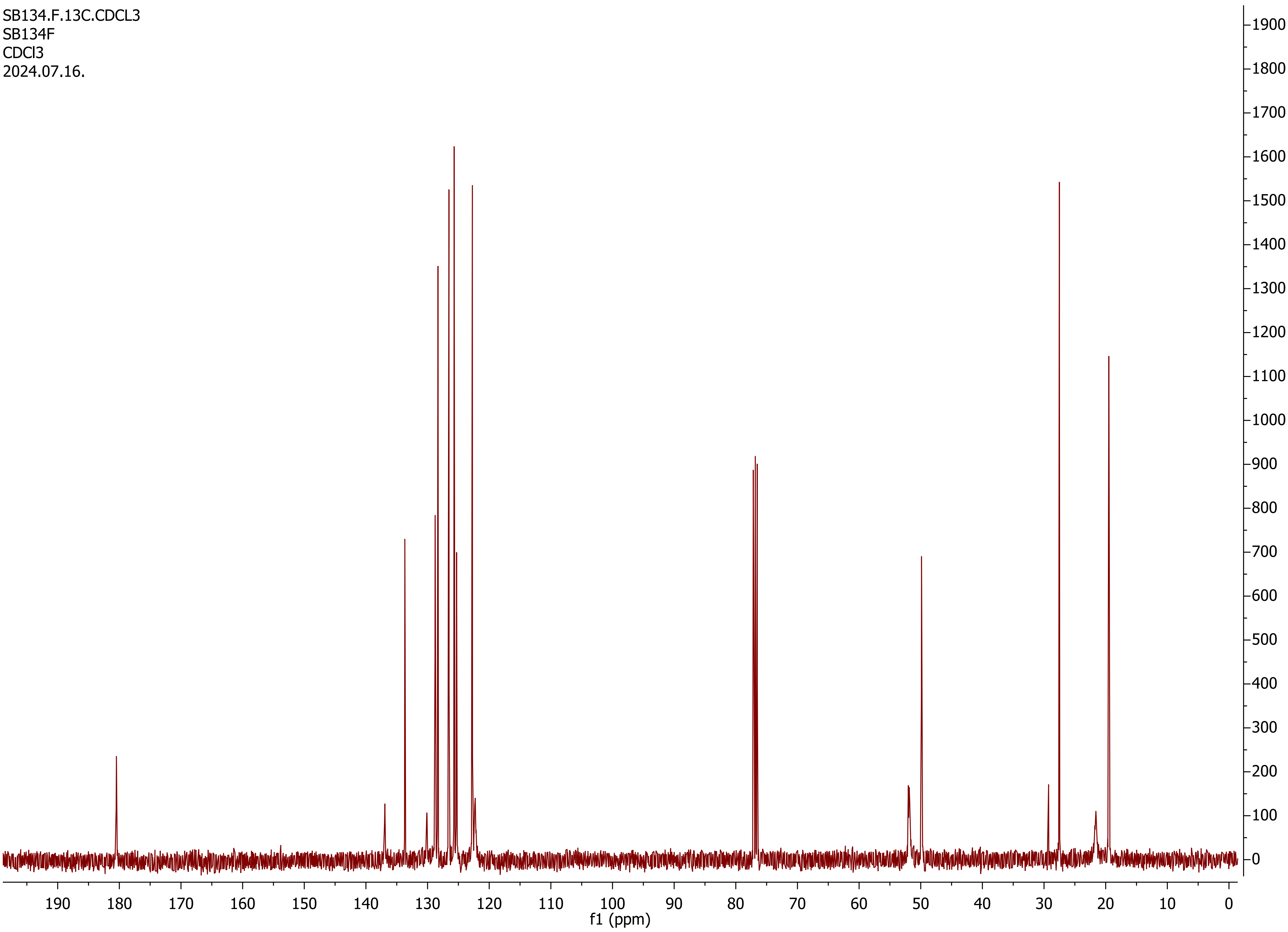

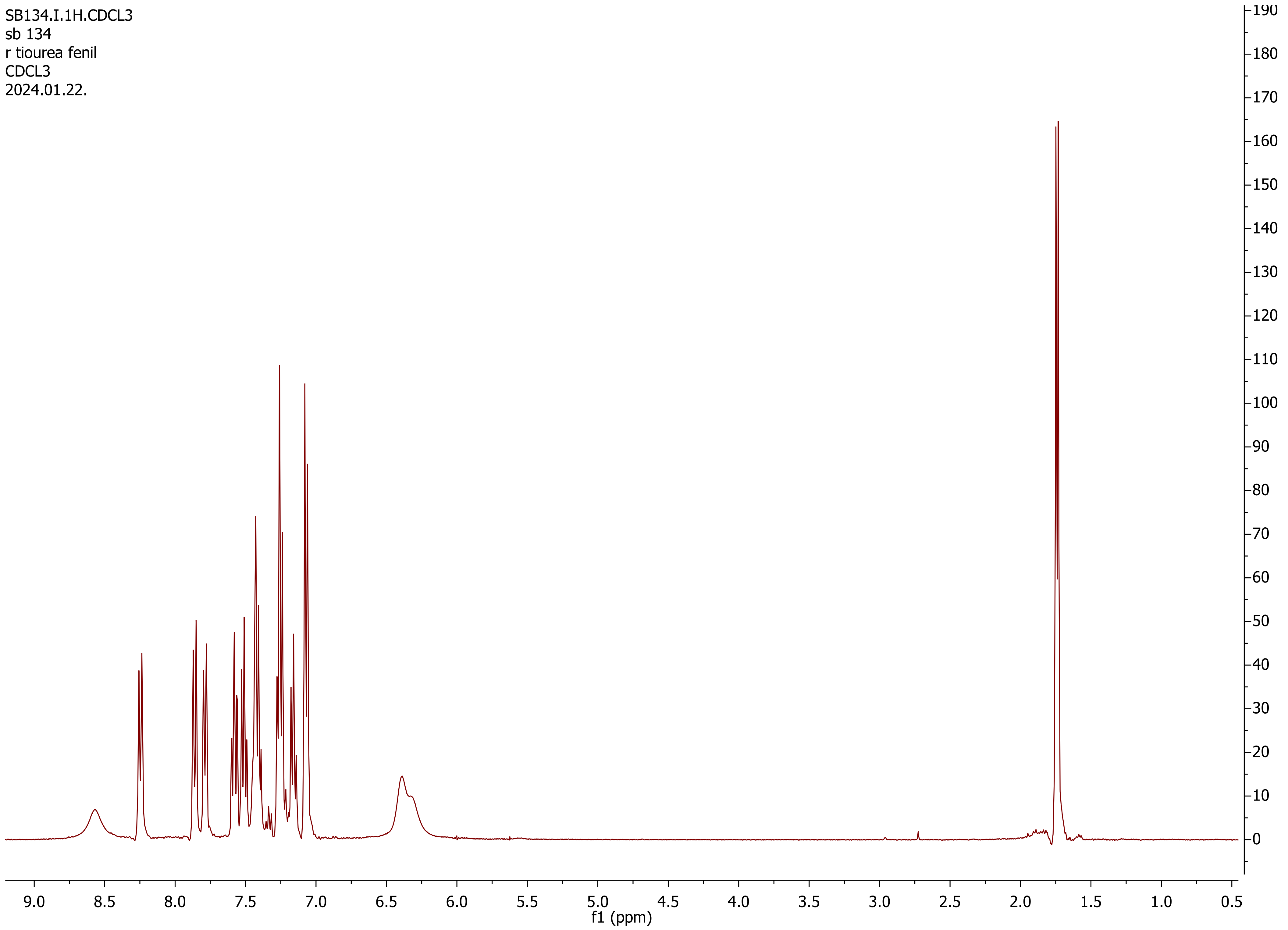
SB134.I.1H.CDCL3
sb 134
r tiourea fenil
CDCL3
2024.01.22.
f1 (ppm)

SB134.I.13C.CDCL3
sb 134 i
r fenil tiourea
CDCL3
2024.01.22.

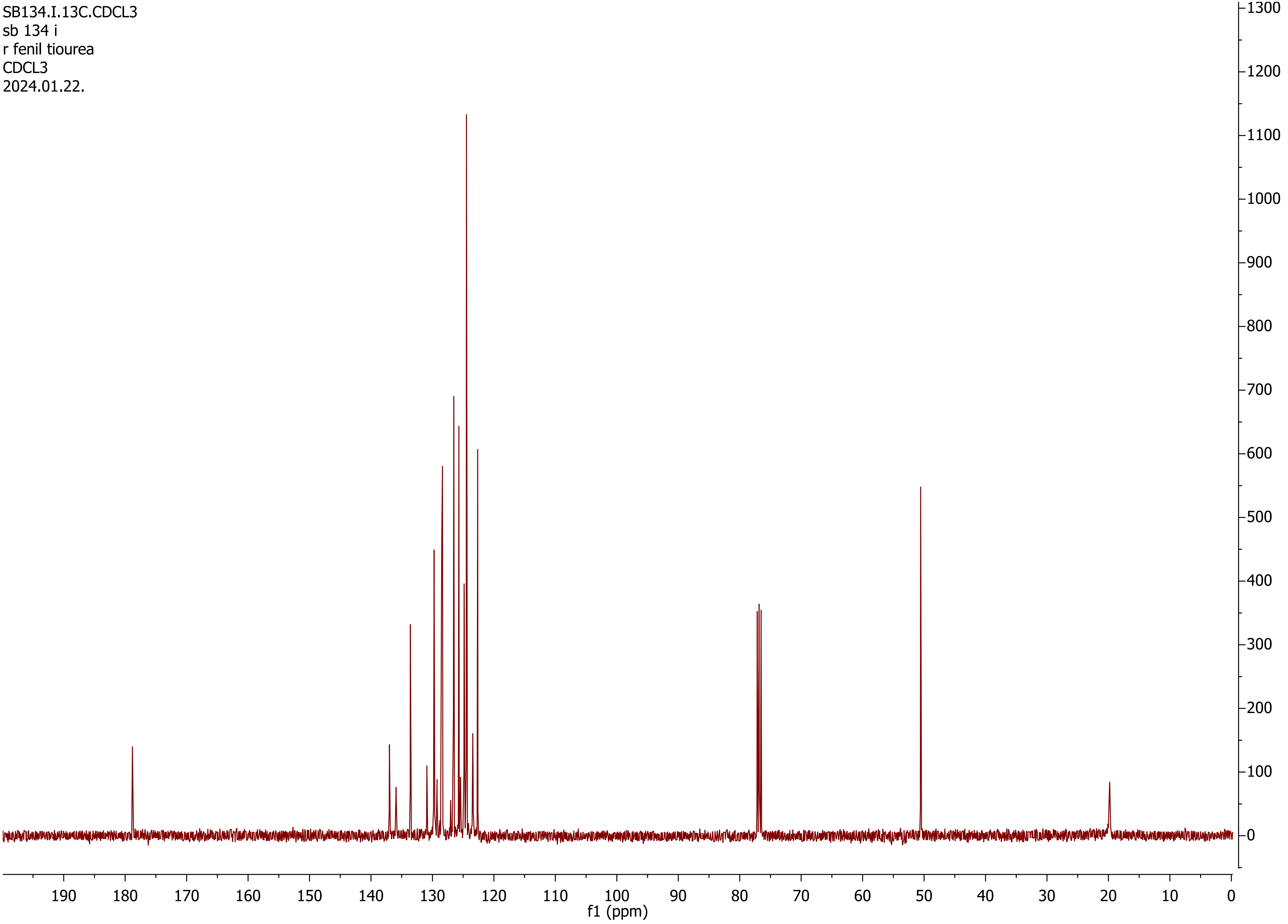

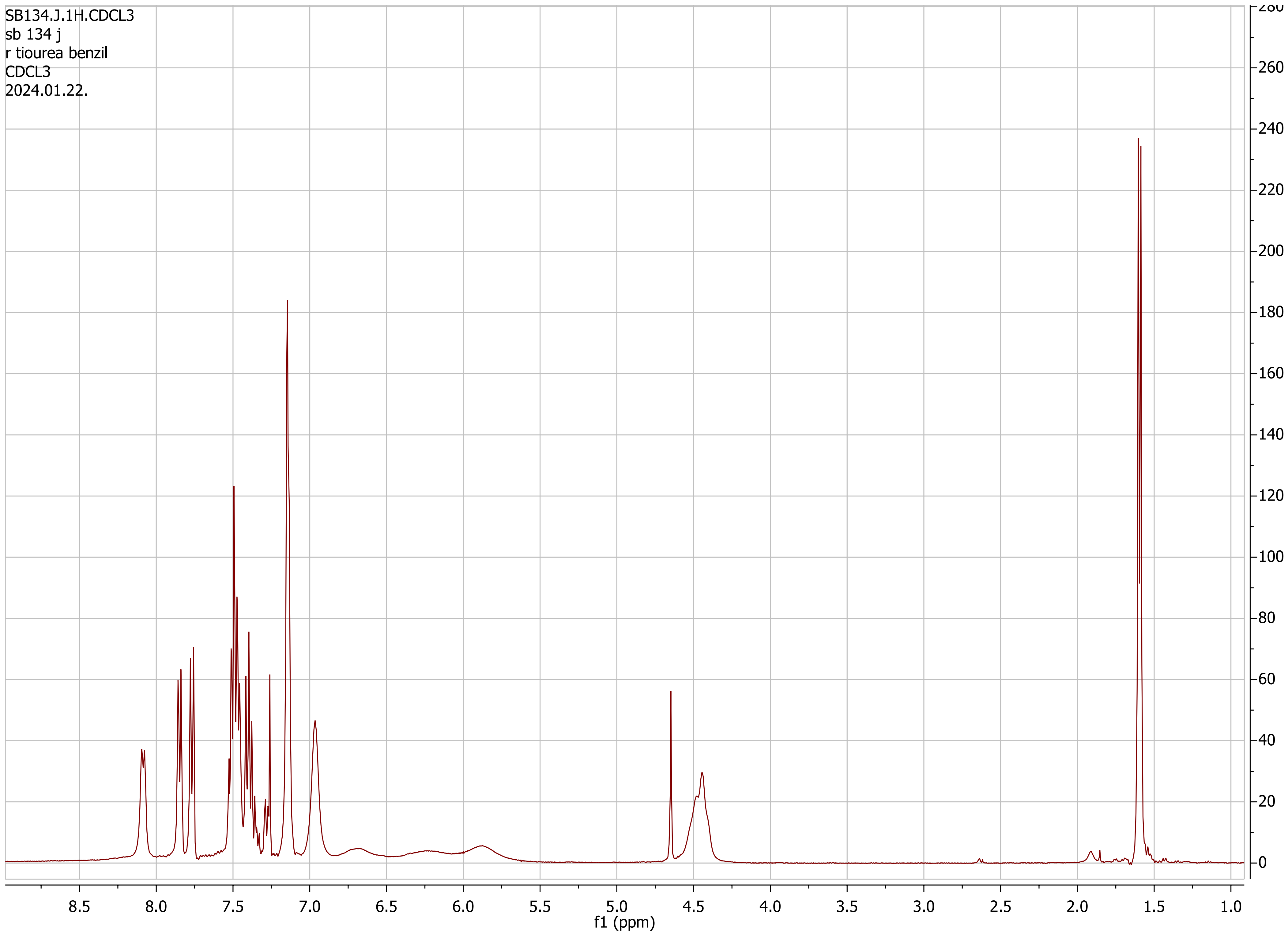

SB134.J.1H.CDCL3
sb 134 j
r tiourea benzil
CDCL3
2024.01.22.
f1 (ppm)

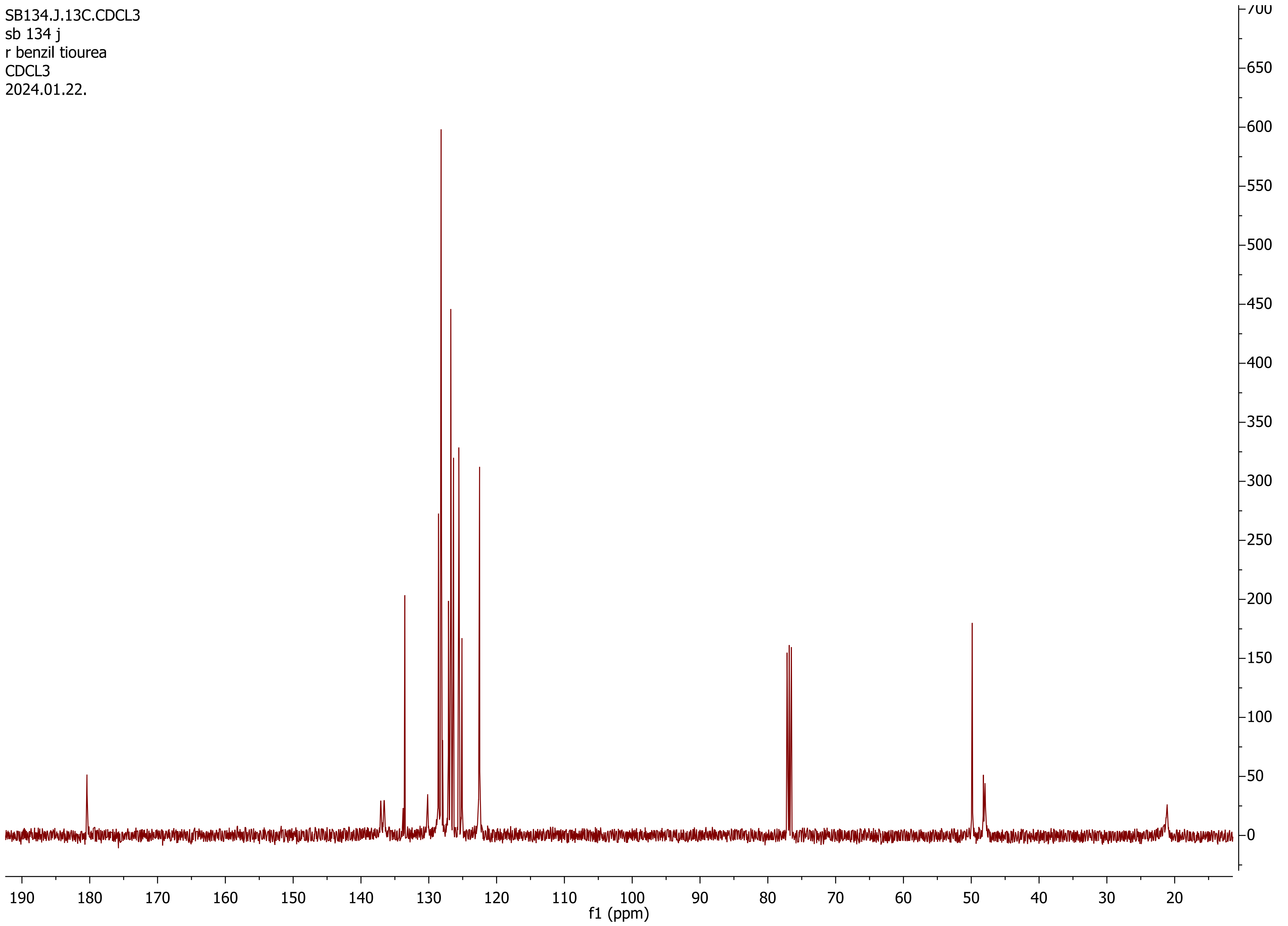
SB134.J.13C.CDCL3
sb 134 j
r benzil tiourea
CDCL3
2024.01.22.
f1 (ppm)

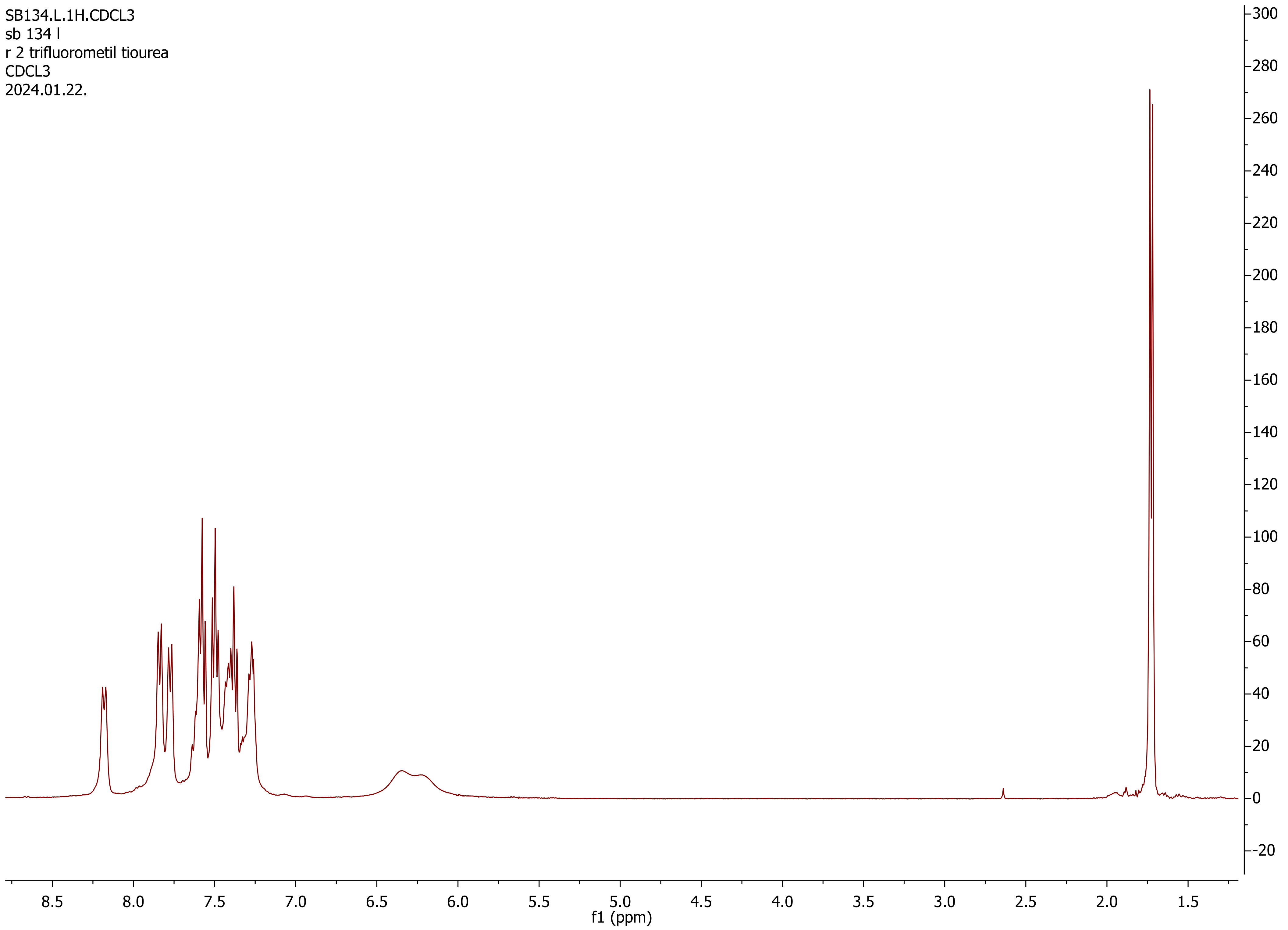
SB134.L.1H.CDCL3
sb 134 l
r 2 trifluorometil tiourea
CDCL3
2024.01.22.
8.5
8.0
7.5
7.0
6.5
6.0
5.5
5.0
4.5
4.0
3.5
3.0
2.5
2.0
1.5
f1 (ppm)
300
280
260
240
220
200
180
160
140
120
100
80
60
40
20
0
-20

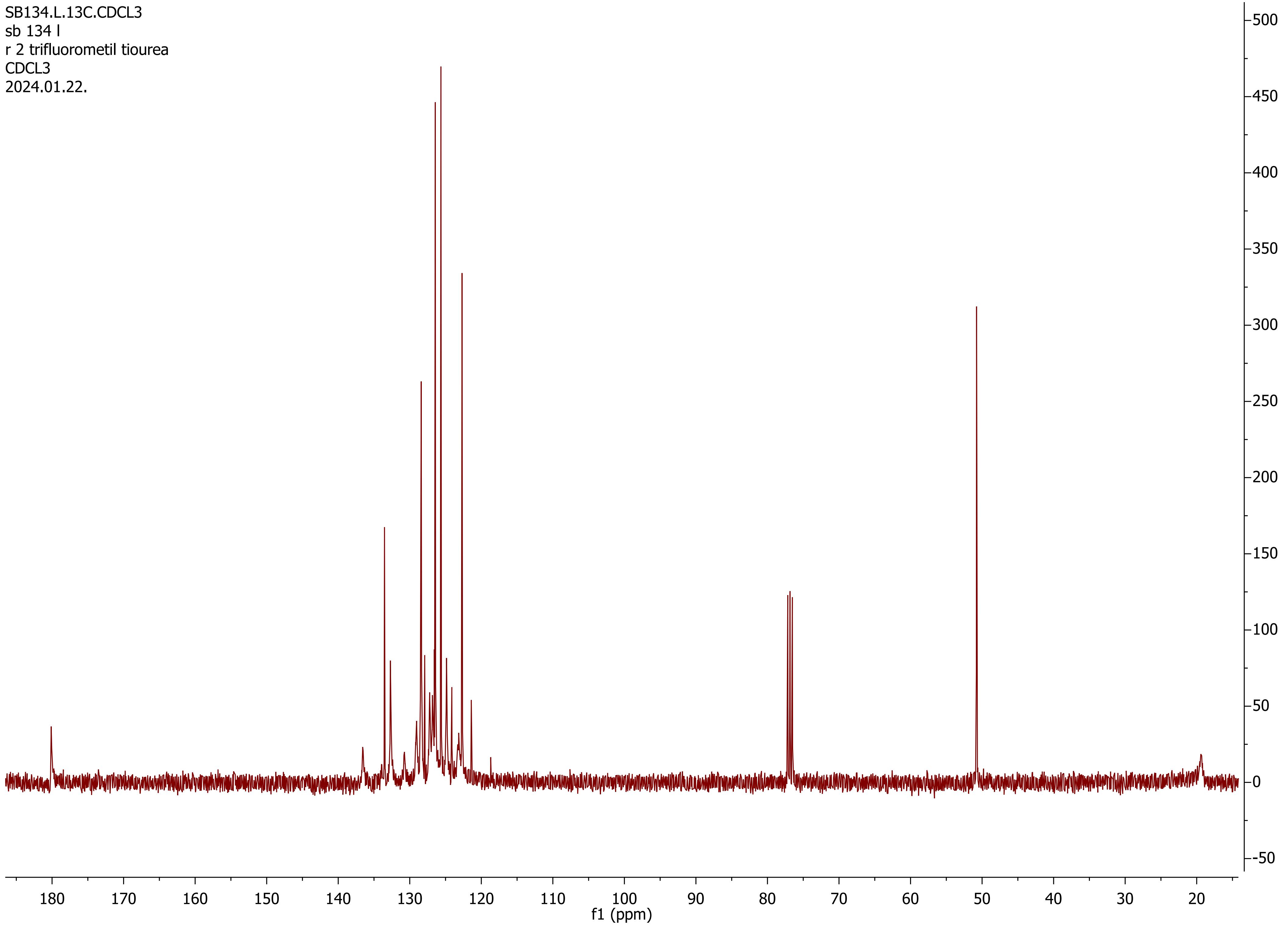

SB134.L.13C.CDCL3
sb 134 l
r 2 trifluorometil tiourea
CDCL3
2024.01.22.
f1 (ppm)

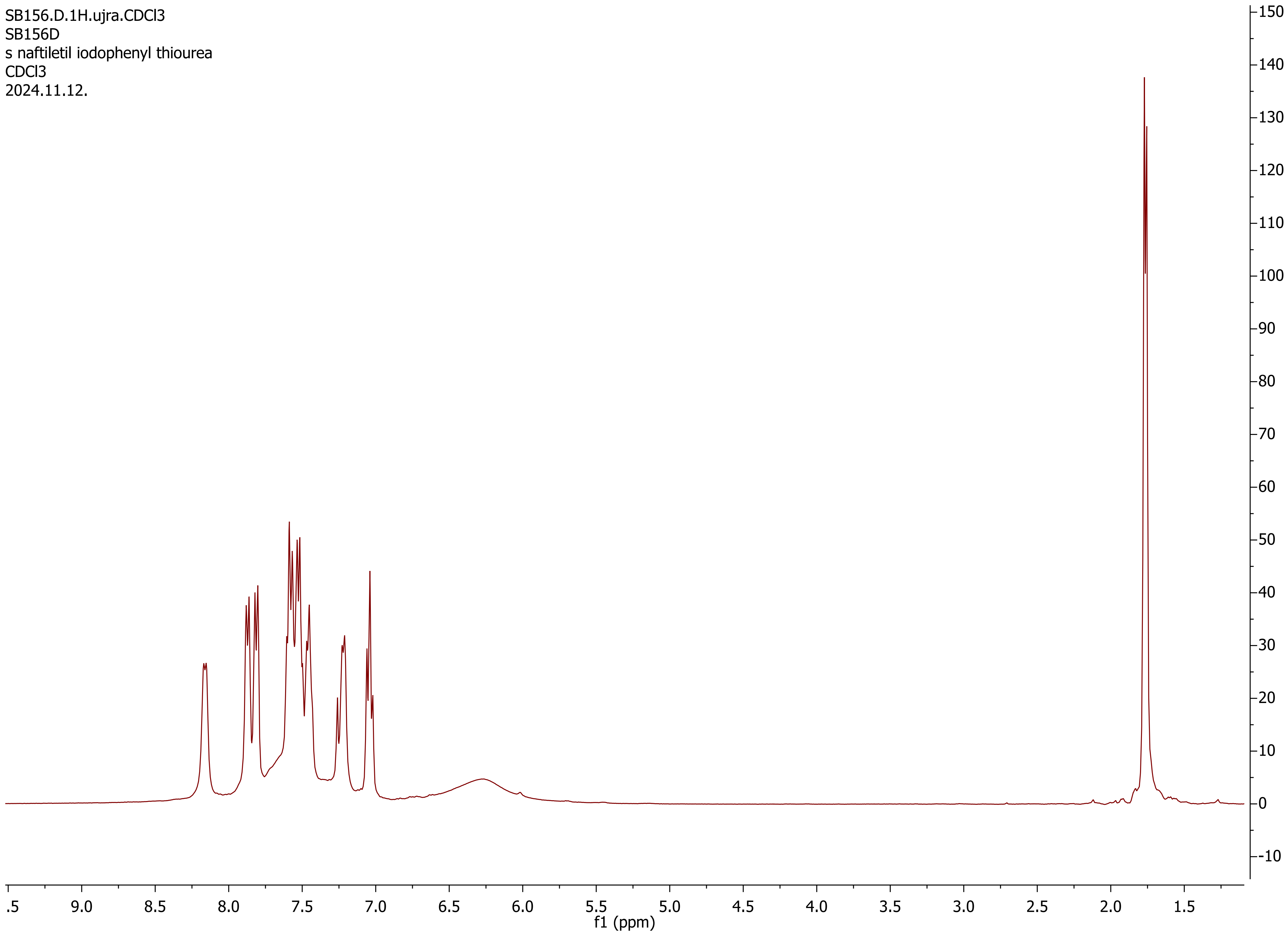
SB156.D.1H.ujra.CDCl3
SB156D
s naftiletil iodophenyl thiourea
CDCl3
2024.11.12.
f1 (ppm)

SB156.D.13C.ujra.CDCl3
SB156D
s naftiletil iodophenyl thiourea
CDCl3
2024.11.12.

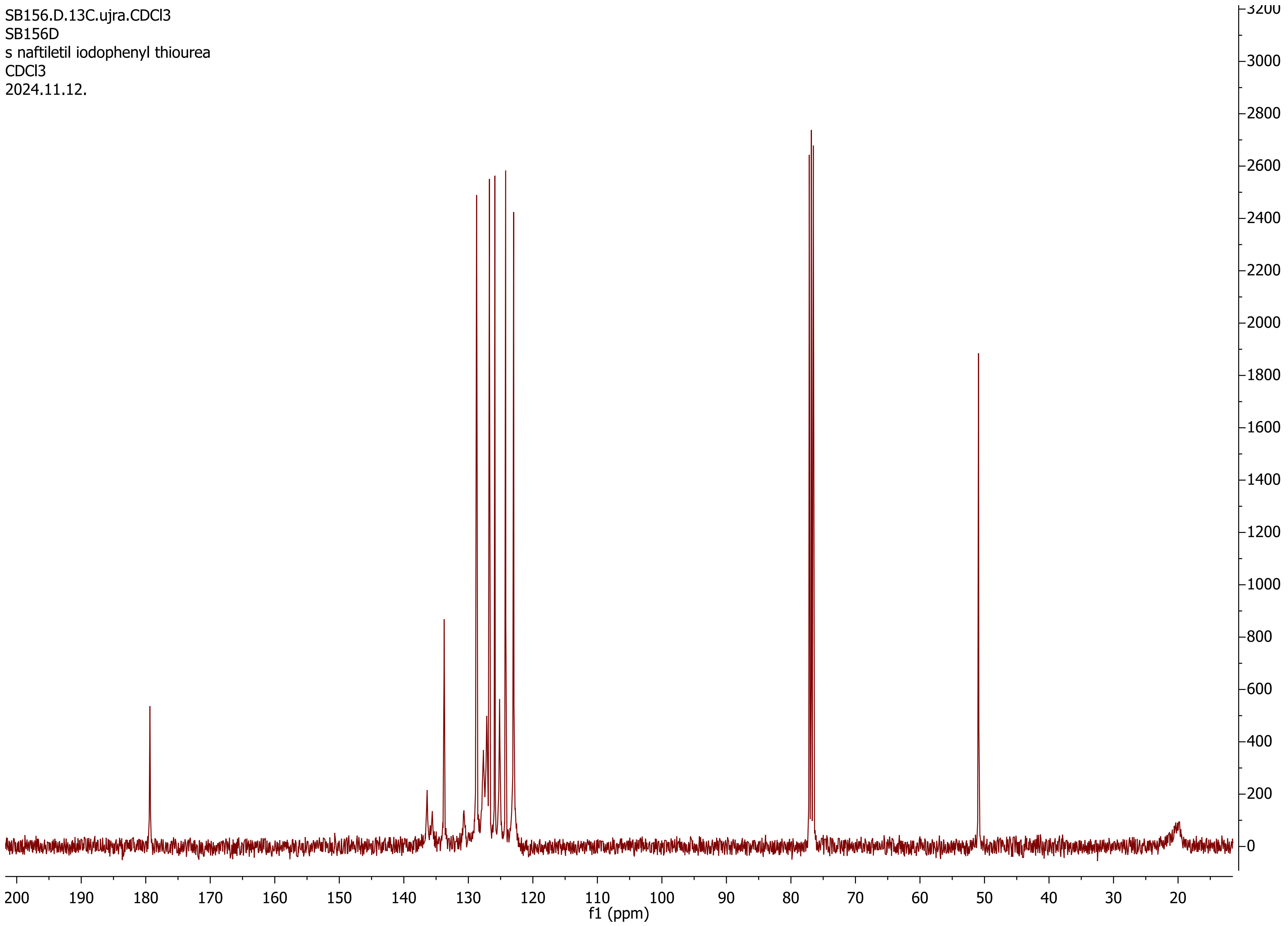

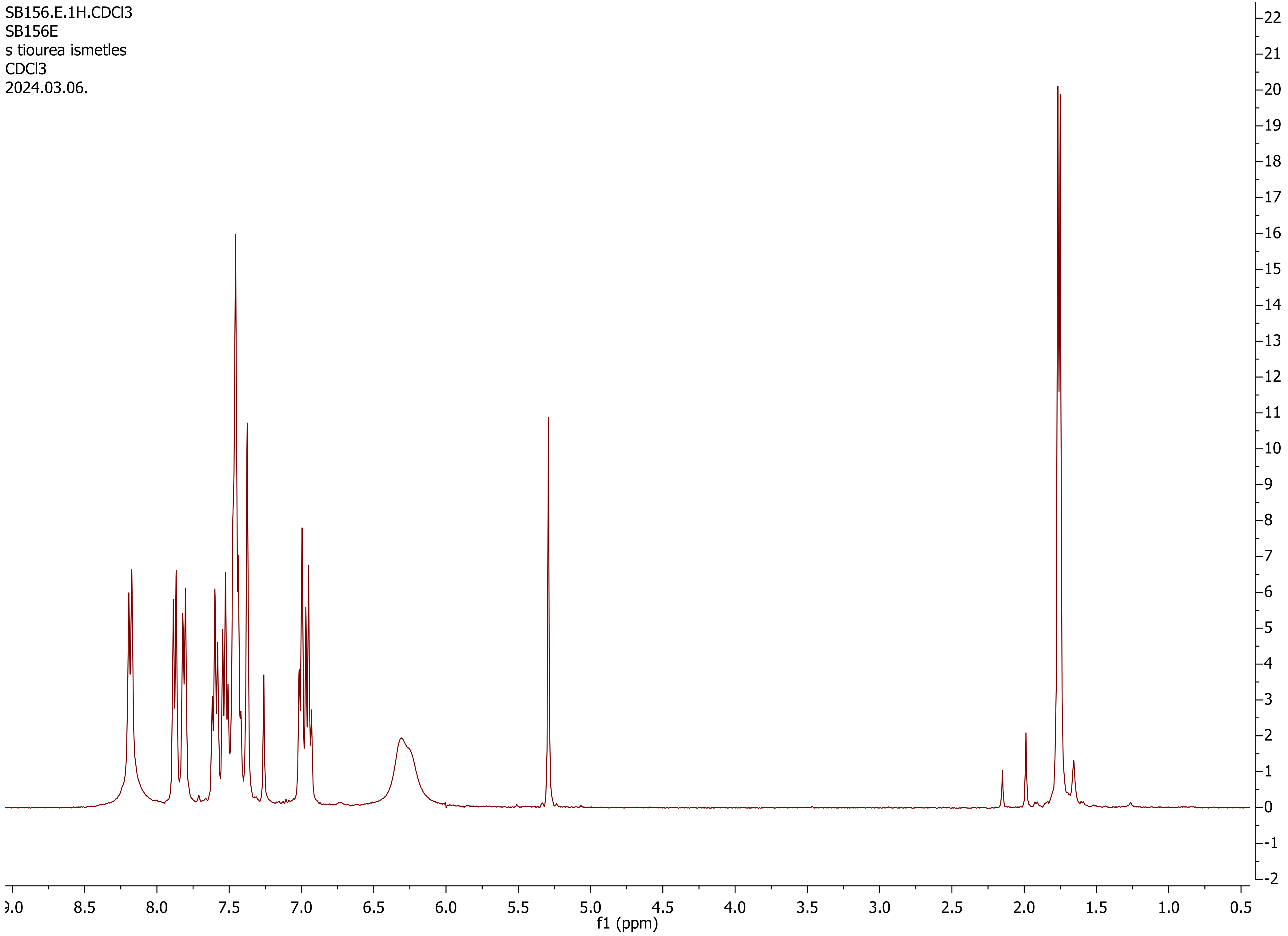

SB156.E.1H.CDCl3
SB156E
s tiourea ismetles
CDCl3
2024.03.06.
f1 (ppm)

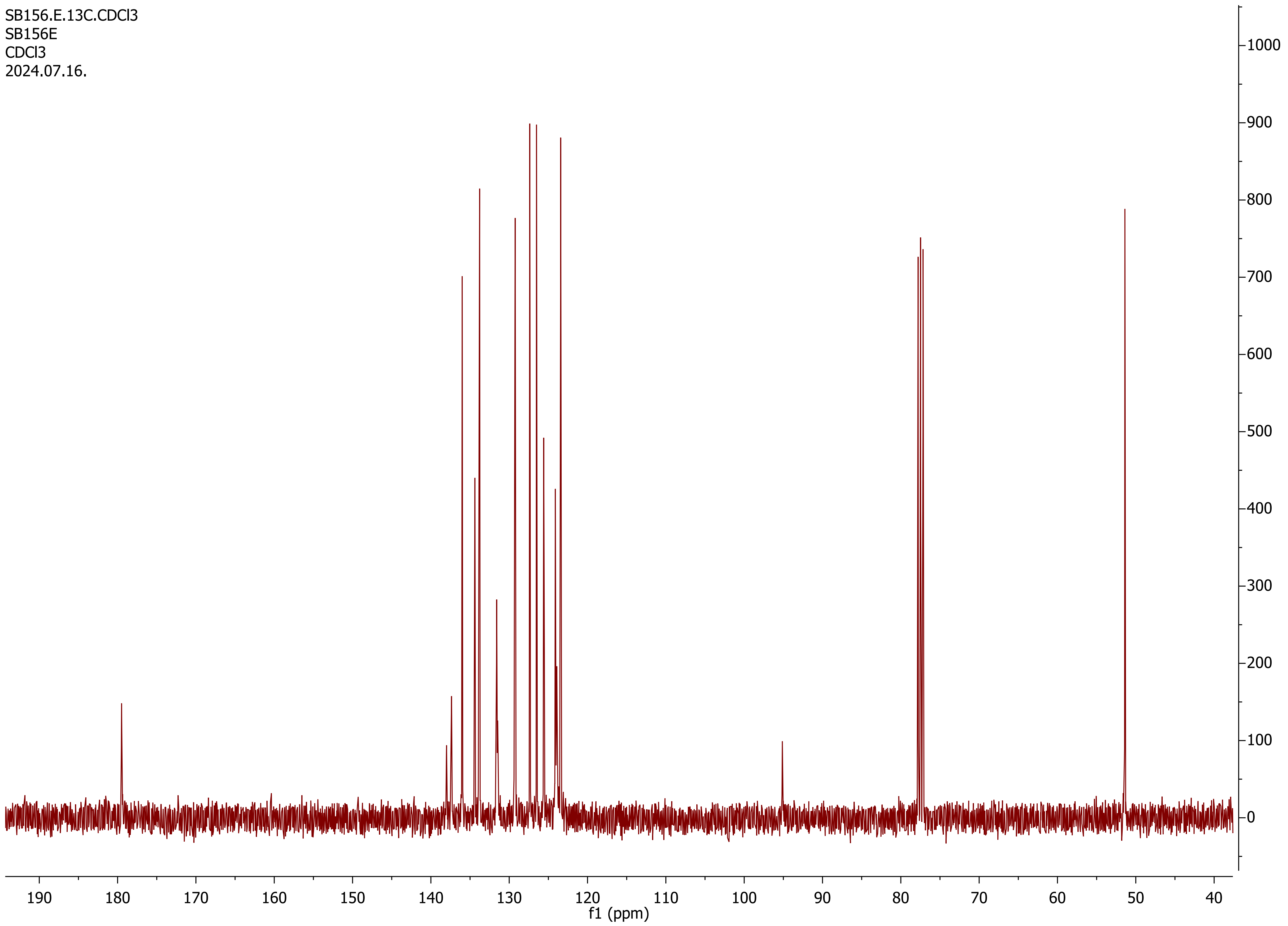

SB156.E.13C.CDCl3
SB156E
CDCl3
2024.07.16.
f1 (ppm)

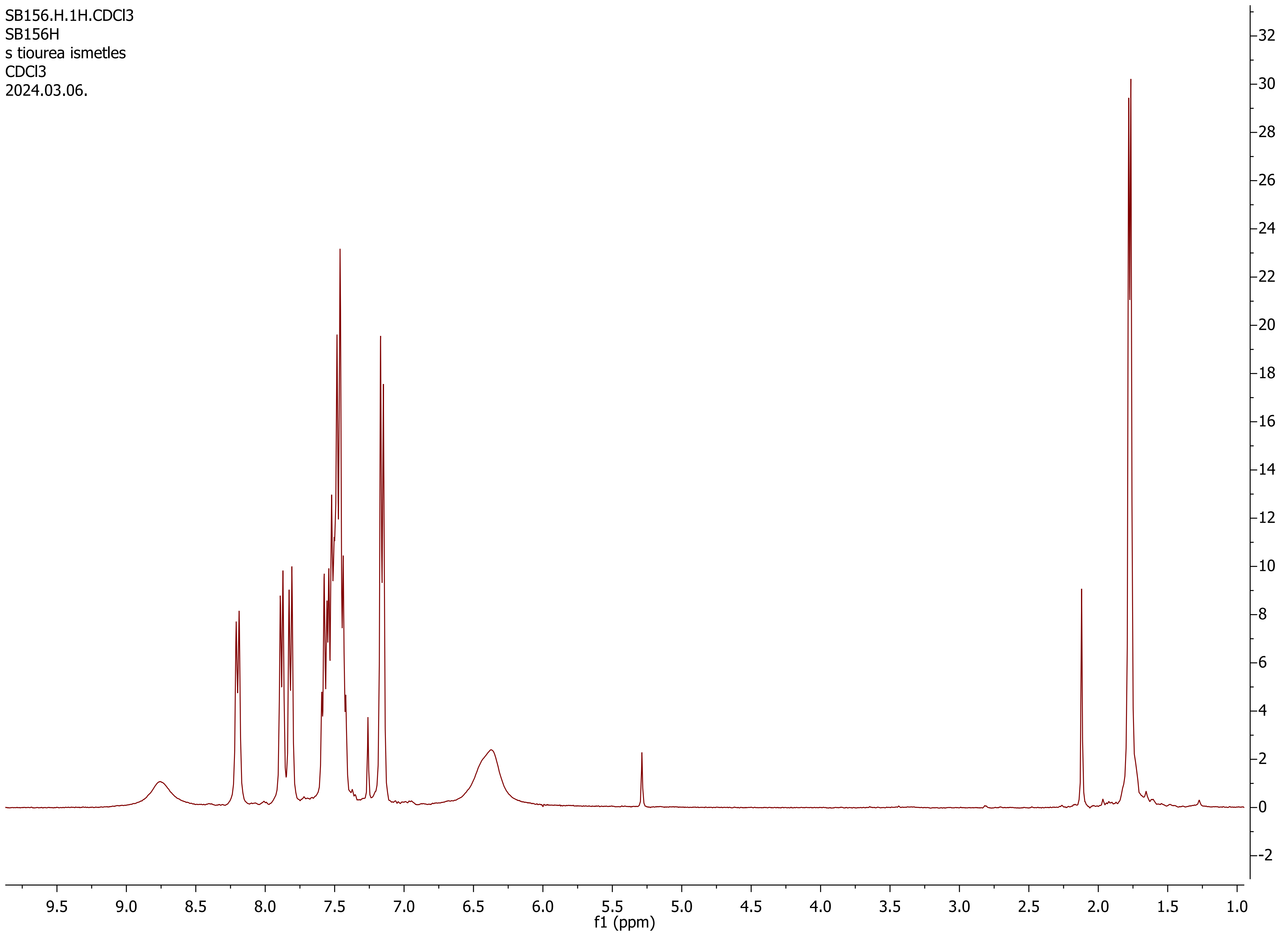
SB156.H.1H.CDCl3
SB156H
s tiourea ismetles
CDCl3
2024.03.06.
f1 (ppm)
9.5
9.0
8.5
8.0
7.5
7.0
6.5
6.0
5.5
5.0
4.5
4.0
3.5
3.0
2.5
2.0
1.5
1.0
32
30
28
26
24
22
20
18
16
14
12
10
8
6
4
2
0
-2

SB156.H.13C.CDCl3
SB156H
CDCl3
2024.07.18.

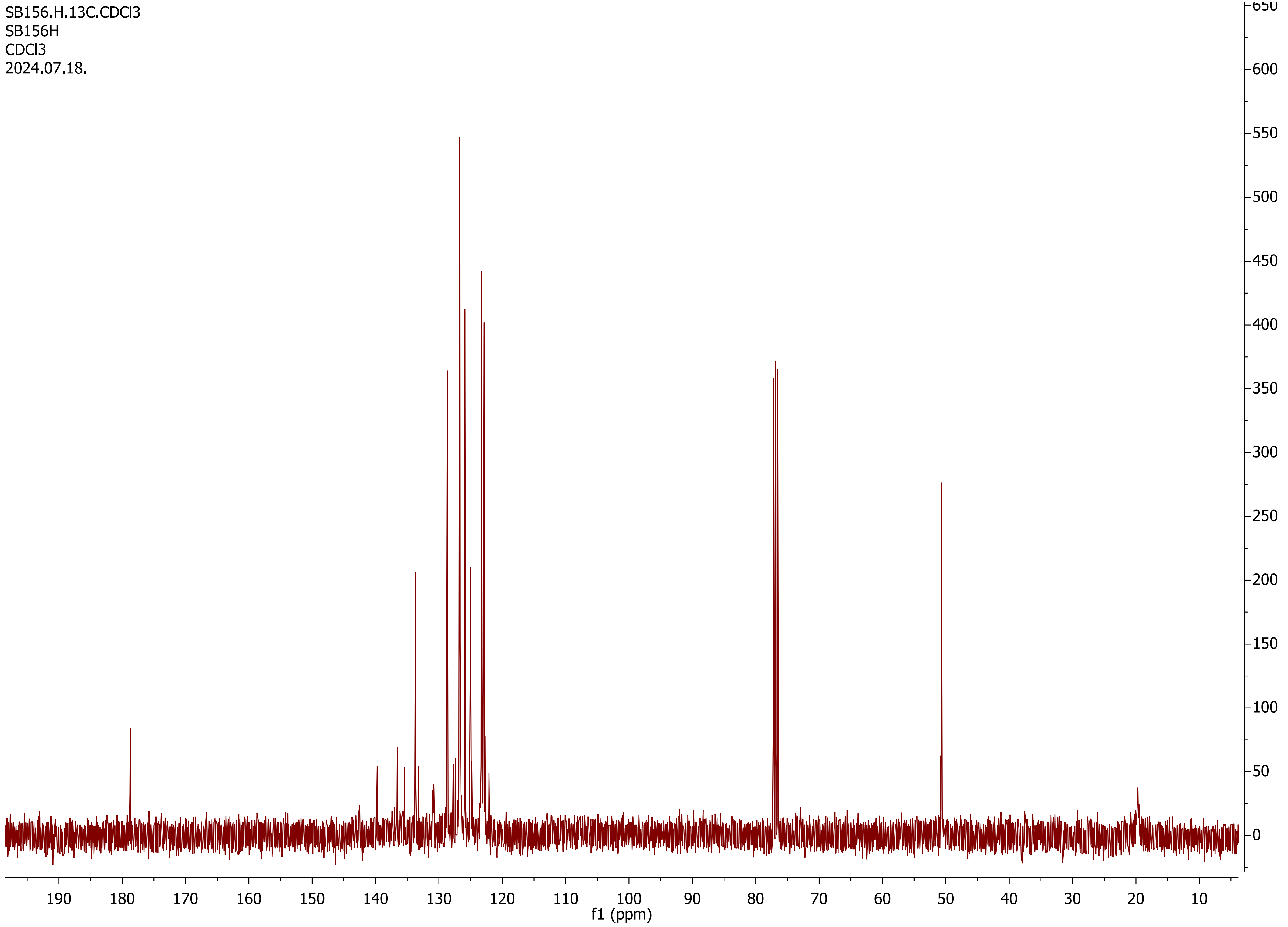

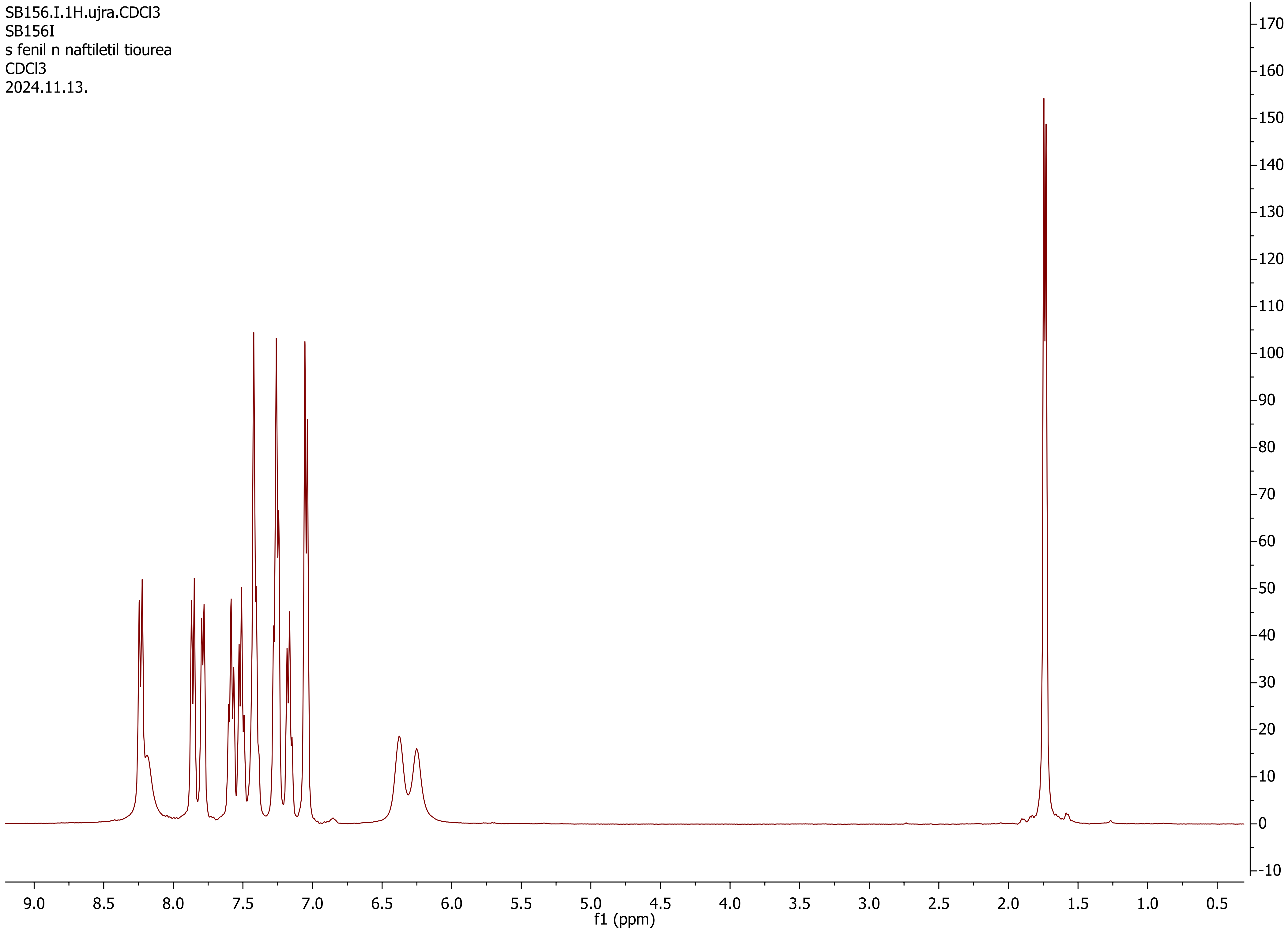
SB156.I.1H.ujra.CDCl3
SB156I
s fenil n naftiletil tiourea
CDCl3
2024.11.13.
f1 (ppm)

SB156.I.13C.ujra.CDCl3
SB156I
s fenil n naftiletil tiourea
CDCl3
2024.11.13.

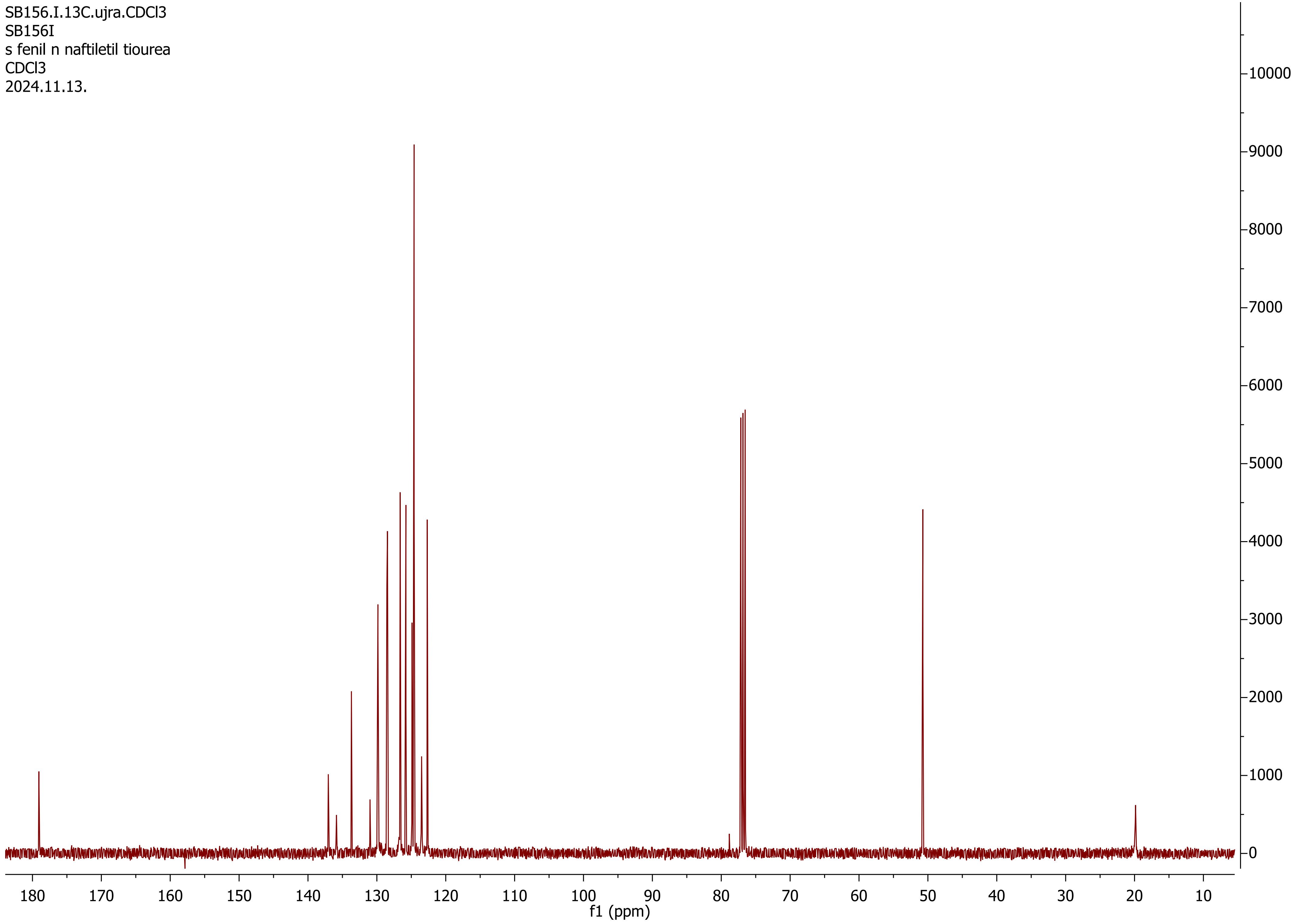

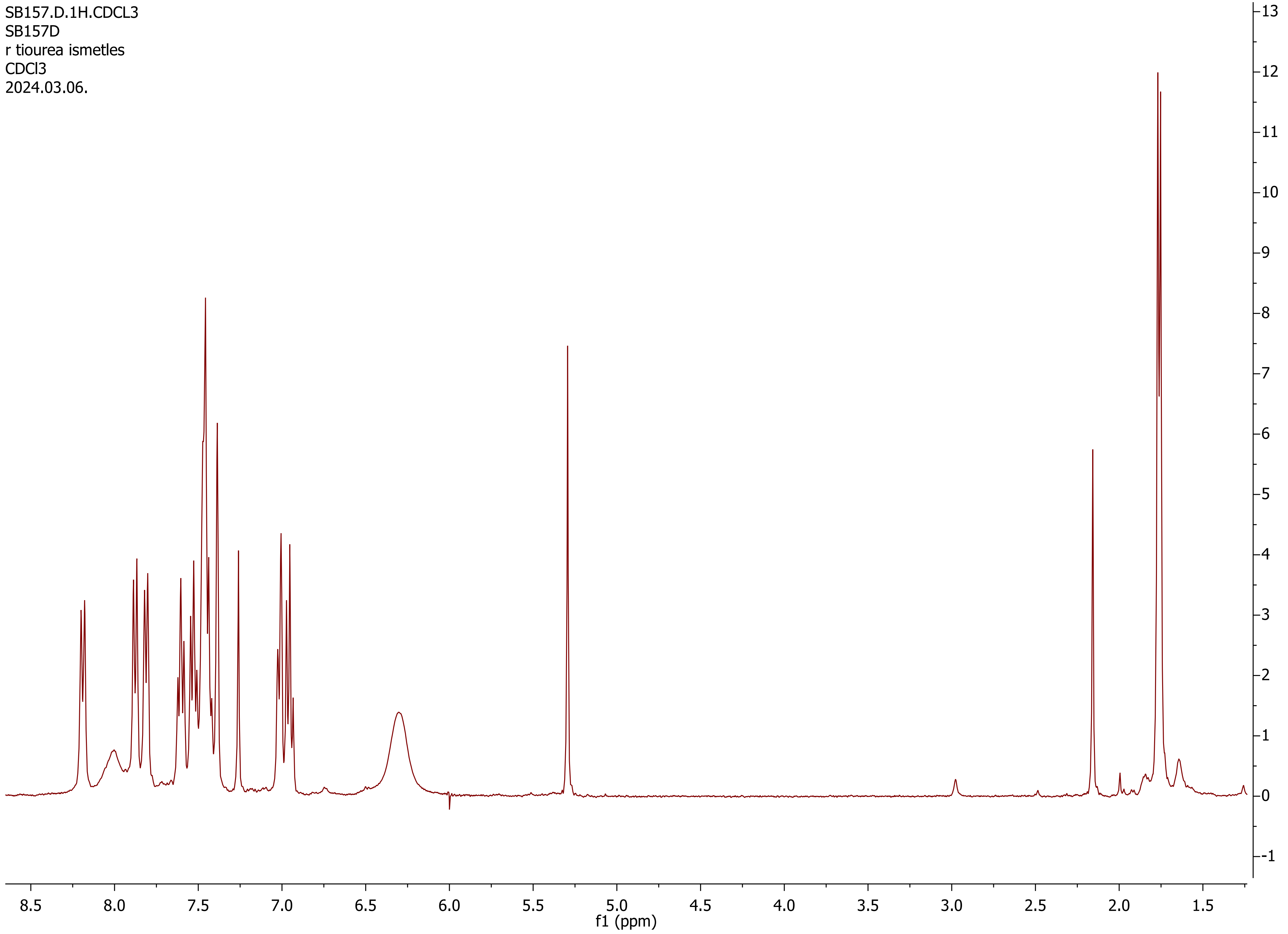
SB157.D.1H.CDCL3
SB157D
r tiourea ismetles
CDCl3
2024.03.06.
f1 (ppm)

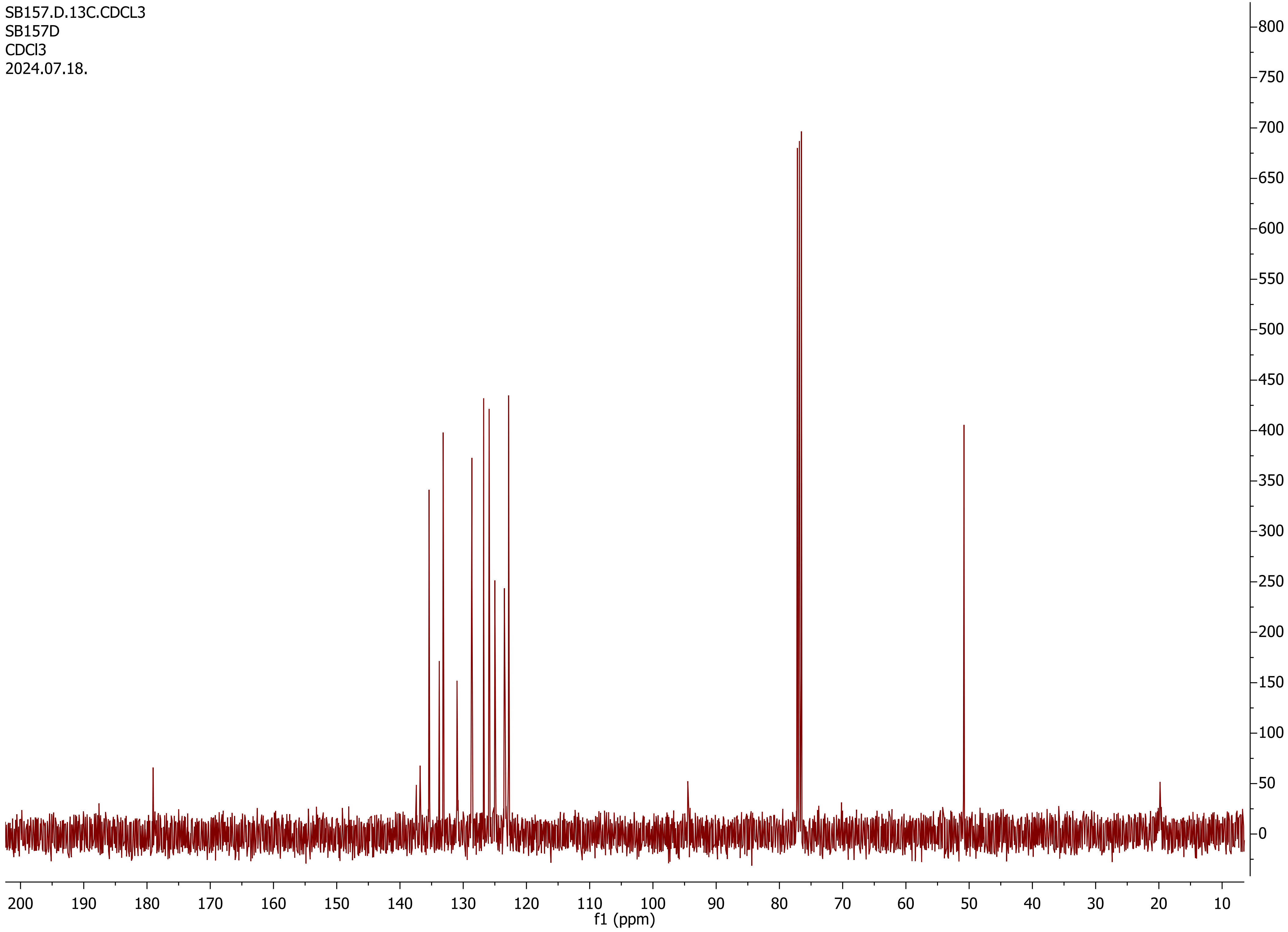
SB157.D.13C.CDCL3
SB157D
CDCl3
2024.07.18.
f1 (ppm)

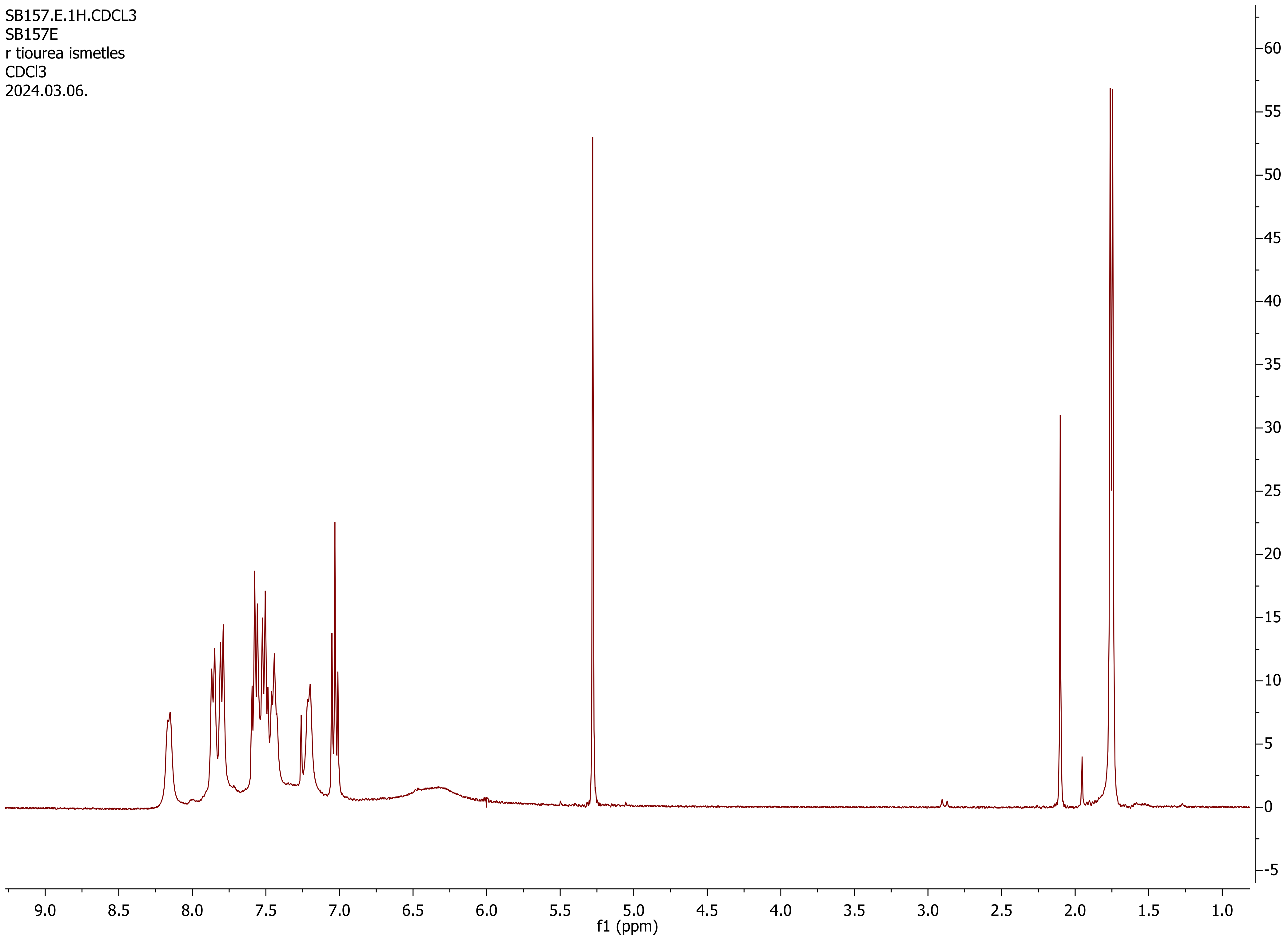
SB157.E.1H.CDCL3
SB157E
r tiourea ismetles
CDCl3
2024.03.06.
f1 (ppm)
9.0
8.5
8.0
7.5
7.0
6.5
6.0
5.5
5.0
4.5
4.0
3.5
3.0
2.5
2.0
1.5
1.0
60
55
50
45
40
35
30
25
20
15
10
5
0
-5

SB157.E.13C.CDCL3
SB157E
CDCl3
2024.07.18.

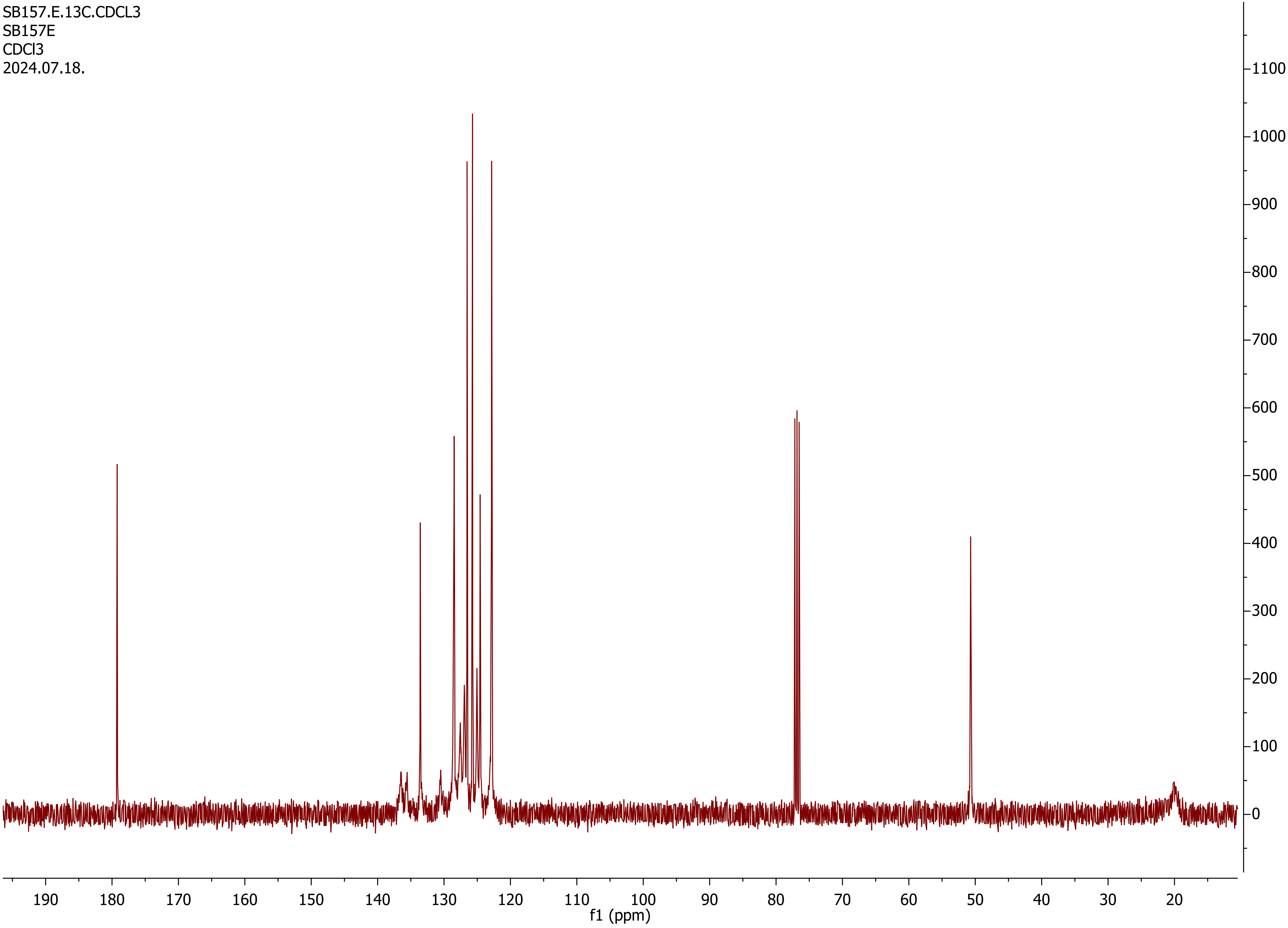

SB157.H.1H.ujra.CDCL3
SB157H
r trifluorometil n naftiletil tiourea
CDCl3
2024.11.13.

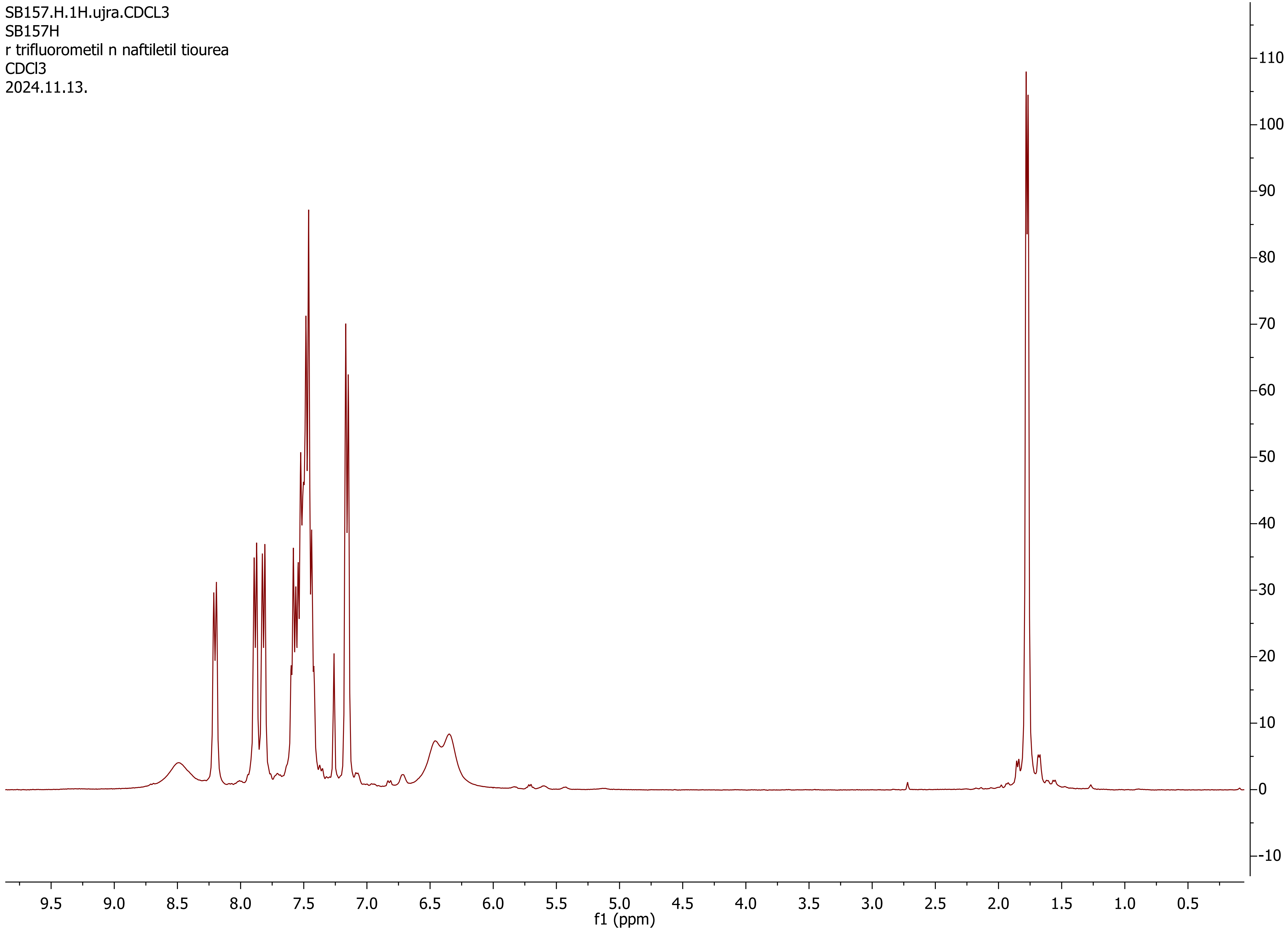

SB157.H.13C.ujra.CDCL3
SB157H
r trifluorometil n naftiletil tiourea
CDCl3
2024.11.13.

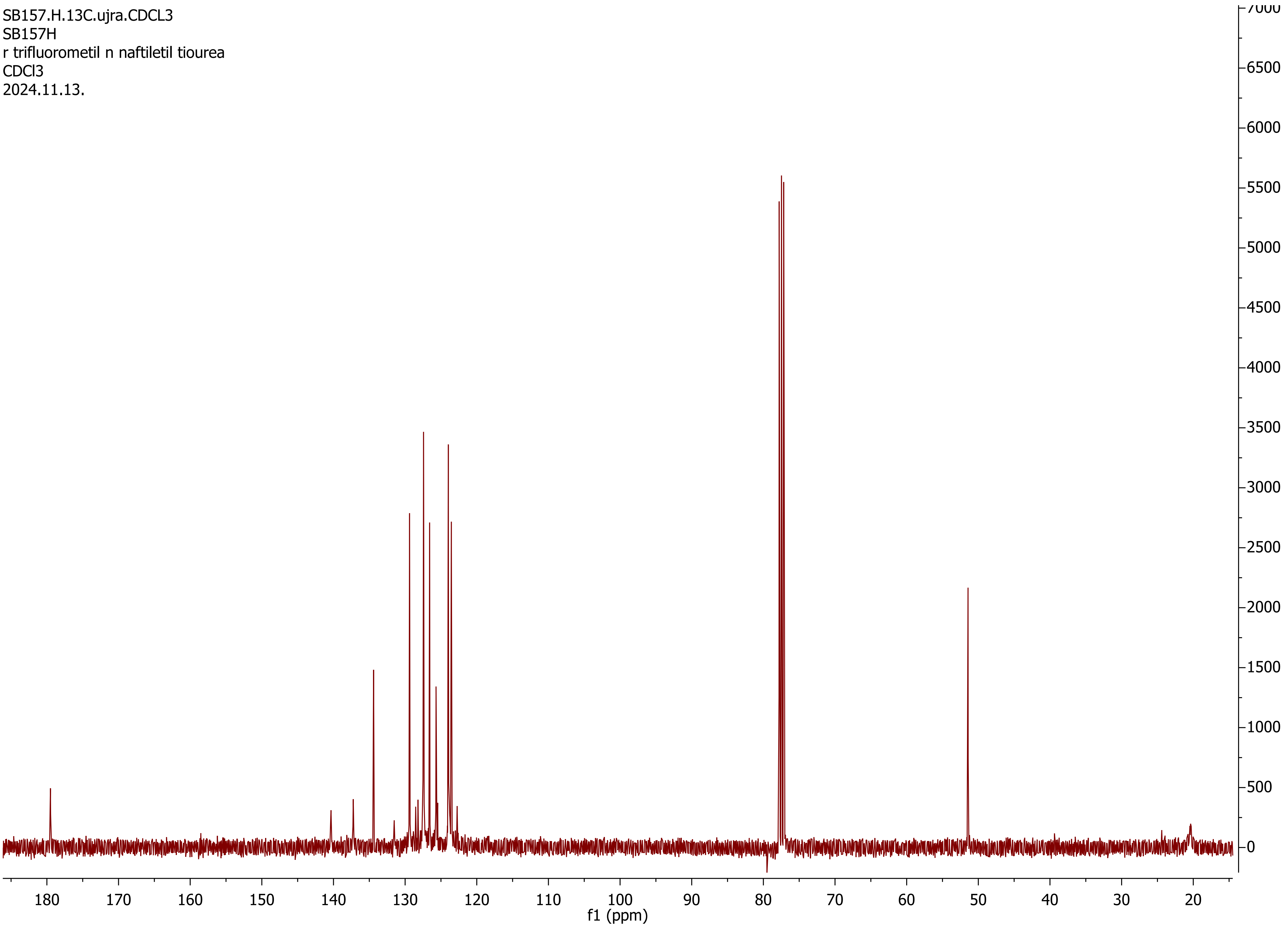

Supplement: Supplementary file 1 [file pharmaceutics-18-01129-s001.zip › tioureanmr_merged.pdf]
