# Supplementary material for: Enantiomer-Resolved Biological Profiling and Chiral Developability Assessment of Novel Naphthylethyl Thioureas
Source: Pharmaceutics. 2026 Sep 8;18(9):1129. doi: 10.3390/pharmaceutics18091129 (PMC13610919; doi:10.3390/pharmaceutics18091129)
Supplement: Supplementary file 1 [file pharmaceutics-18-01129-s001.zip › tiourea_supporting_FINAL.pdf]

SUPPORTING INFORMATION

Enantiomer-Resolved Biological Profiling and Chiral Developability Assessment of Novel Naphthylethyl Thioureas

Gergely Molnár[1,2], Balázs Simon[1,2], Arash Mirzahosseini[1,2], Máté Dobó[1,2], Gergely Dombi[1,2], Béla Fiser[3,4,5], Ali Mhammad[1,2], Vivien Bárdos[1,2], Rita Szolláth[1,2], Zoltán-István Szabó[6,7], András Marton[8], Kamilla Varga[2,9], Tamás Tábi[2,9], Imre Boldizsár[2,10,11], Andrea Horváth[12], Orsolya Dobay[12], Szilvia Bősze[13,14], György Tibor Balogh[1,2], Gergő Tóth[1,2*]

[1] Department of Pharmaceutical Chemistry, Semmelweis University, Hőgyes E. street 9, 1092, Budapest, Hungary, molnar.gergely@stud.semmelweis.hu (G.M.), simon.balazs@semmelweis.hu (B.S.), mirzahosseini.arash@semmelweis.hu (A.M.), dobo.mate@stud.semmelweis.hu (M.D.), dombi.gergely@semmelweis.hu (G.D.), ali.mhammad@phd.semmelweis.hu (A.M.), bardos.vivien@semmelweis.hu (V.B.), szollath.rita.mariann@semmelweis.hu (R. Sz.), balogh.gyorgy.tibor@semmelweis.hu (Gy.T.B.), toth.gergo@semmelweis.hu (G.T).

[2] Center for Pharmacology and Drug Research & Development, Semmelweis University, 1085, Budapest, Hungary

[3] Institute of Chemistry, University of Miskolc, H-3515 Miskolc, Hungary, bela.fiser@uni-miskolc.hu

[4] Ferenc Rakoczi II Transcarpathian Hungarian University, 90200 Beregszász, Ukraine

[5] Department of Physical Chemistry, Faculty of Chemistry, University of Lodz, 90-149 Lodz, Poland

[6] George Emil Palade University of Medicine, Pharmacy, Science, and Technology of Targu Mures, Târgu Mureș, Romania, zoltan.szabo@umfst.ro (Z-I. Sz.)

[7] Sz-imfidum Ltd, RO-525401, Lunga, Romania

[8] Department of Chemical and Environmental Process Engineering, Faculty of Chemical Technology and Biotechnology, Budapest University of Technology and Economics, Műegyetem rkp. 3., H-1111 Budapest, Hungary, amarton@edu.bme.hu (A.M)

[9] Department of Pharmacodynamics, Semmelweis University, Budapest, Hungary, tabi.tamas@semmelweis.hu (T.T.), kamillavogelvarga@gmail.com (K.V.)

[10] Department of Pharmacognosy, Semmelweis University, Üllői út 26, Budapest 1085, Hungary, boldizsar.imre@semmelweis.hu (I.B.)

[11] Department of Plant Anatomy, Institute of Biology, Eötvös Loránd University, Pázmány Péter sétány 1/C, Budapest 1117, Hungary

[12] Institute of Medical Microbiology, Semmelweis University, Budapest, Hungary, horvath.andrea1@semmelweis.hu (A.H.), dobay.orsolya@semmelweis.hu (O.D.)

[13] HUN-REN-ELTE Research Group of Peptide Chemistry, Hungarian Research Network (HUN-REN), Eötvös Loránd University, Pázmány Péter sétány 1/A, Budapest 1117, Hungary, szilvia.bosze@ttk.elte.hu (Sz.B.)

[14] Department of Genetics, Cell- and Immunobiology, Faculty of Medicine, Semmelweis University, Nagyvárad tér 4, H-1089 Budapest, Hungary

Supporting Information S1. Detailed spectroscopic characterization of newly synthesized thioureas

1-[(1S)-1-(naphthalen-1-yl)ethyl]-3-(propan-2-yl)thiourea (Compound 1S) yellow oil, yield: 83%, ^{1}H NMR (400 MHz, $CDCl_3$) δ 8.11 (d, J = 8.5 Hz, 1H), 7.91 – 7.83 (m, 1H), 7.80 (d, J = 8.2 Hz, 1H), 7.62 – 7.38 (m, 4H), 6.36 (s, 1H), 5.79 (s, 1H), 4.11 (s, 1H), 3.68 (q, J = 7.0 Hz, 1H), 1.68 (d, J = 6.7 Hz, 3H), 1.20 (t, J = 7.0 Hz, 1H), 1.04 (d, J = 6.5 Hz, 3H), 0.82 (d, J = 6.4 Hz, 3H).^{13}C NMR (101 MHz, $CDCl_3$) δ 179.79, 137.75, 134.21, 130.82, 129.30, 128.81, 127.05, 126.23, 125.77, 123.29, 123.09, 50.30, 46.55, 22.71, 22.49, 21.92. IR (ATR): 3280.32, 2966.95, 1528.31. $[\alpha]_D^{20}$=21.7 (c=0.3, MeOH). HRMS ESI pos. calcd. for $C16H21N2S^+$ $[M+H]^+$ 273.1425 found 273.1420, HRMS ESI neg. calcd. for $C16H19N2S^-$ $[M-H]^-$ 271.1269 found 271.1272.

1-[(1R)-1-(naphthalen-1-yl)ethyl]-3-(propan-2-yl)thiourea (Compound 1R) yellow oil, yield: 82%: ^{1}H NMR (400 MHz, $CDCl_3$) δ 8.11 (d, J = 8.4 Hz, 1H), 7.89 – 7.82 (m, 1H), 7.78 (d, J = 8.2 Hz, 1H), 7.58 – 7.36 (m, 4H), 6.46 (s, 1H), 5.81 (s, 1H), 4.11 (s, 1H), 1.65 (d, J = 6.7 Hz, 3H), 1.33 (d, J = 6.4 Hz, 1H), 1.02 (d, J = 6.4 Hz, 3H), 0.82 (d, J = 5.9 Hz, 3H). ^{13}C NMR (101 MHz, $CDCl_3$) δ 179.56, 137.35, 133.96, 130.52, 129.05, 128.59, 126.81, 125.97, 125.50, 123.03, 122.76, 50.06, 46.29, 22.44, 22.19, 21.68. IR (ATR): 3245.61, 2969.84, 1534.02. $[\alpha]_D^{20}$=-21.7 (c=0.3, MeOH). HRMS ESI pos. calcd. for $C16H21N2S^+$ $[M+H]^+$ 273.1425 found 273.1420, HRMS ESI neg. calcd. for $C16H19N2S^-$ $[M-H]^-$ 271.1269 found 271.1272.

1-[(1S)-1-(naphthalen-1-yl)ethyl]-3-propylthiourea (Compound 2S) pale-yellow oil, yield: 78.5%: ^{1}H NMR (400 MHz, $CDCl_3$) δ 8.11 (d, J = 8.4 Hz, 1H), 7.89 (dd, J = 8.1, 1.5 Hz, 1H), 7.81 (d, J = 8.2 Hz, 1H), 7.63 – 7.42 (m, 4H), 5.78 (s, 1H), 3.46 (t, J = 6.6 Hz, 1H), 3.27 – 3.21 (m, 2H), 1.69 (d, J = 6.6 Hz, 3H), 1.58 – 1.16 (m, 2H), 1.12 – 0.81 (m, 1H), 0.66 (t, J = 7.4 Hz, 3H). ^{13}C NMR (101 MHz, $CDCl_3$) δ 180.83, 137.15, 134.12, 130.58, 129.19, 128.80, 126.95, 126.12, 125.66, 123.09, 122.80, 50.33, 46.43, 22.12, 11.08. IR (ATR): 3253.32, 2965.02, 1536.99. $[\alpha]_D^{20}$=35.3 (c=0.25, MeOH). HRMS ESI pos. calcd. for $C16H21N2S^+$ $[M+H]^+$ 273.1425 found 273.1426, HRMS ESI neg. calcd. for $C16H19N2S^-$ $[M-H]^-$ 271.1269 found 271.1272.

1-[(1R)-1-(naphthalen-1-yl)ethyl]-3-propylthiourea (Compound 2R) pale-yellow oil, yield: 79%: ^{1}H NMR (400 MHz, $CDCl_3$) δ 8.11 (d, J = 8.5 Hz, 1H), 7.92 – 7.80 (m, 1H), 7.77 (d, J = 8.2 Hz, 1H), 7.58 – 7.44 (m, 3H), 7.41 (t, J = 7.7 Hz, 1H), 5.84 (s, 1H), 3.42 (t, J = 6.6 Hz, 1H), 3.20 (s, 2H), 1.63 (d, J = 6.7 Hz, 3H), 1.31 (q, J = 7.4 Hz, 2H), 0.98 (t, J = 7.4 Hz, 1H), 0.64 (t, J = 7.2 Hz, 3H). ^{13}C NMR (101 MHz, $CDCl_3$) δ 181.14, 137.50, 134.43, 130.90, 129.50, 129.10, 127.26, 126.43, 125.97, 123.40, 123.13, 50.64, 46.74, 22.44, 11.40. IR (ATR): 3245.36, 2962.13, 1536.62. $[\alpha]_D^{20}$=-35.3 (c=0.25, MeOH). HRMS ESI pos. calcd. for $C16H21N2S^+$ $[M+H]^+$ 273.1425 found 273.1426, HRMS ESI neg. calcd. for $C16H19N2S^-$ $[M-H]^-$ 271.1269 found 271.1272,

3-[3-(methylsulfanyl)propyl]-1-[(1S)-1-(naphthalen-1-yl)ethyl]thiourea (Compound 3S) yellowish-green oil, yield: 83%: ^{1}H NMR (400 MHz, $CDCl_3$) δ 8.08 (d, J = 8.4 Hz, 1H), 7.88 – 7.79 (m, 1H), 7.75 (d, J = 8.2 Hz, 1H), 7.50 (ddd, J = 15.3, 6.9, 1.3 Hz, 2H), 7.47 – 7.36 (m, 1H), 6.32 – 5.44 (m, 1H), 3.60 (t, J = 6.4 Hz, 1H), 3.39 (d, J = 38.9 Hz, 2H), 2.55 (t, J = 7.0 Hz, 1H), 2.20 (s, 2H), 2.05 (s, 1H), 1.90 (p, J = 6.8 Hz, 1H), 1.69 (s, 3H), 1.61 (t, J = 11.0 Hz, 5H). ^{13}C NMR (101 MHz, $CDCl_3$) δ 180.98, 137.52, 134.32, 130.85, 129.36, 128.90, 127.07, 126.29, 125.91, 123.12, 50.54, 43.96, 31.74, 31.19, 29.41, 27.79, 15.34. IR (ATR): 3255.25 2968.87 1536.06, $[\alpha]_D^{20}$=8.3 (c=0.3, MeOH). HRMS ESI pos. calcd. for C17H23N2S2+ $[M^+H]^+$ 319.1303 found 319.1301, HRMS ESI neg. calcd. for C17H21N2S2$^-$ $[M-H]^-$ 317.1146 found 317.115,

3-[3-(methylsulfanyl)propyl]-1-[(1R)-1-(naphthalen-1-yl)ethyl]thiourea (Compound 3R) greenish oil, yield: 74%: ^{1}H NMR (400 MHz, $CDCl_3$) δ 8.07 (d, J = 8.4 Hz, 1H), 7.84 (dd, J = 8.0, 1.5 Hz, 1H), 7.76 (d, J = 8.2 Hz, 1H), 7.49 (dddd, J = 21.7, 8.0, 6.9, 1.3 Hz, 3H), 7.43 – 7.36 (m, 1H), 6.72 (s, 1H), 3.61 (t, J = 6.4 Hz, 1H), 3.36 (d, J = 12.5 Hz, 1H), 2.55 (t, J = 6.9 Hz, 1H), 2.18 (d, J = 23.8 Hz, 2H), 2.06 (s, 2H), 1.91 (p, J = 6.7 Hz, 1H), 1.76 – 1.47 (m, 7H). ^{13}C NMR (101 MHz, $CDCl_3$) δ 180.86, 137.55, 134.25, 130.82, 129.29, 128.81, 127.01, 126.24, 125.84, 123.07, 50.47, 43.91, 31.68, 31.13, 29.35, 27.78, 15.30. IR (ATR): 3246.57, 2968.87, 1536.02. $[\alpha]_D^{20}$=-8.3 (c=0.3, MeOH). HRMS ESI pos. calcd. for C17H23N2S2$^+$ $[M+H]^+$ 319.1303 found 319.1301, HRMS ESI neg. calcd. for C17H21N2S2$^-$ $[M-H]^-$ 317.1146 found 317.115.

1-(3-iodophenyl)-3-[(1S)-1-(naphthalen-1-yl)ethyl]thiourea (Compound 4S) light brown oil, yield: 83%: ^{1}H NMR (400 MHz, $CDCl_3$) δ 8.15 (d, J = 8.4 Hz, 1H), 7.88 (d, J = 8.1 Hz, 1H), 7.82 (d, J = 8.2 Hz, 1H), 7.68 – 7.39 (m, 4H), 7.31 – 7.17 (m, 1H), 7.05 (t, J = 8.0 Hz, 1H), 6.27 (s, 2H), 5.29 (s, 1H), 2.15 (d, J = 1.2 Hz, 1H), 1.77 (d, J = 6.6 Hz, 3H). ^{13}C NMR (101 MHz, $CDCl_3$) δ 179.34, 136.39, 135.62, 133.75, 130.66, 128.72, 127.67, 127.13, 126.73, 125.89, 125.16, 124.22, 122.97, 50.93, 19.84. IR (ATR): 3191.61 2972.73 1505.17. $[\alpha]_D^{20}$=38.9 (c=0.4, MeOH). HRMS ESI pos. calcd. for C19H18IN2S$^+$ $[M+H]^+$ 433.0235 found 433.0242, HRMS ESI neg. calcd. for C19H16IN2S$^-$ $[M-H]^-$ 431.0079, found 431.0095,

1-(3-iodophenyl)-3-[(1R)-1-(naphthalen-1-yl)ethyl]thiourea (Compound 4R) light brown oil, yield: 82%: ^{1}H NMR (400 MHz, $CDCl_3$) δ 8.19 (d, J = 8.5 Hz, 1H), 7.88 (d, J = 8.1 Hz, 1H), 7.81 (d, J = 7.8 Hz, 1H), 7.67 – 7.56 (m, 1H), 7.53 (t, J = 7.5 Hz, 1H), 7.46 (t, J = 7.0 Hz, 2H), 7.39 (t, J = 1.9 Hz, 1H), 7.08 – 6.88 (m, 2H), 6.30 (s, 1H), 5.29 (s, 1H), 2.16 (s, 1H), 1.76 (d, J = 6.5 Hz, 3H). ^{13}C NMR (101 MHz, $CDCl_3$) δ 179.04, 137.43, 136.81, 135.40, 133.78, 133.17, 130.98, 128.71, 128.62, 126.76, 125.90, 125.00, 123.49, 123.33, 122.83, 94.48, 50.81, 19.84. IR (ATR): 3194.54 3001.66 1573.63. $[\alpha]_D^{20}$=-38.9 (c=0.4, MeOH). HRMS ESI pos. calcd. for C19H18IN2S$^+$ $[M+H]^+$ 433.0235 found 433.0242, HRMS ESI neg. calcd. for C19H16IN2S$^-$ $[M-H]^-$ 431.0079, found 431.0095,

1-(2,3-dichlorophenyl)-3-[(1S)-1-(naphthalen-1-yl)ethyl]thiourea (Compound 5S) colorless oil, yield: 82%: ^{1}H NMR (400 MHz, $CDCl_3$) δ 8.18 (d, J = 8.6 Hz, 1H), 7.88 (d, J = 8.1 Hz, 1H),

7.81 (d, J = 7.6 Hz, 1H), 7.68 – 7.40 (m, 4H), 7.38 (s, 1H), 7.12 (d, J = 8.1 Hz, 2H), 6.30 (s, 1H), 5.29 (s, 1H), 1.76 (d, J = 6.5 Hz, 3H). ^{13}C NMR (101 MHz, $CDCl_3$) δ 178.86, 137.37, 136.72, 135.36, 133.74, 133.14, 130.96, 128.68, 128.60, 126.74, 125.87, 124.96, 123.48, 123.28, 122.80, 50.76, 19.76. IR (ATR): 3208.00 2972.73 1525.43. $[\alpha]_D^{20}$=38.6 (c=0.4, MeOH). HRMS ESI pos. calcd. for $C19H17Cl2N2S^+$ $[M+H]^+$ 375.0489 found 375.0484, HRMS ESI neg. calcd. for $C19H15Cl2N2S^-$ $[M-H]^-$ 373.0333, found 373.0335,

1-(2,3-dichlorophenyl)-3-[(1R)-1-(naphthalen-1-yl)ethyl]thiourea (Compound 5R) colorless oil, yield: 81%: ^{1}H NMR (400 MHz, $CDCl_3$) δ 8.18 (d, J = 8.6 Hz, 1H), 7.88 (d, J = 8.1 Hz, 1H), 7.81 (d, J = 7.6 Hz, 1H), 7.68 – 7.40 (m, 4H), 7.38 (s, 1H), 7.12 (d, J = 8.1 Hz, 2H), 6.30 (s, 1H), 5.29 (s, 1H), 1.76 (d, J = 6.5 Hz, 3H). ^{13}C NMR (101 MHz, $CDCl_3$) δ 178.86, 137.37, 136.72, 135.36, 133.74, 133.14, 130.96, 128.68, 128.60, 126.74, 125.87, 124.96, 123.48, 123.28, 122.80, 50.76, 19.76. IR (ATR): 3207.04 3034.44 1534.10, HRMS ESI pos. calcd. for $C19H17Cl2N2S^+$ $[M+H]^+$ 375.0489 found 375.0484. $[\alpha]_D^{20}$=-38.6 (c=0.4, MeOH), HRMS ESI neg. calcd. for $C19H15Cl2N2S^-$ $[M-H]^-$ 373.0333, found 373.0335,

3-(2-methylpropyl)-1-[(1S)-1-(naphthalen-1-yl)ethyl]thiourea (Compound 6S) pale brown oil, yield: 81.5%: ^{1}H NMR (400 MHz, CDCl3) δ 8.10 (d, J = 8.4 Hz, 1H), 7.83 (dd, J = 8.0, 1.5 Hz, 1H), 7.76 (d, J = 8.2 Hz, 1H), 7.60 – 7.35 (m, 4H), 5.89 (br s, 1H), 3.26 (d, J = 6.2 Hz, 1H), 3.09 (m, 2H), 1.61 (d, J = 6.7 Hz, 3H), 0.95 (d, J = 6.7 Hz, 1H), 0.60 (s, 6H). ^{13}C NMR (101 MHz, CDCl3) δ 181.03, 137.61, 134.26, 130.73, 129.34, 128.86, 127.09, 126.27, 125.87, 123.31, 122.95, 52.43, 50.44, 28.11, 22.17, 20.07, 19.98. IR (ATR): 3254.29 2957.30 1537.95, $[\alpha]_D^{20}$=19.8 (c=0.3, MeOH), HRMS ESI pos. calcd. for $C17H23N2S^+$ $[M+H]^+$ 287.1582 found 287.1573 HRMS ESI neg. calcd. for $C17H21N2S^-$ $[M-H]^-$ 285.1425, found 285.1424,

3-(2-methylpropyl)-1-[(1R)-1-(naphthalen-1-yl)ethyl]thiourea (Compound 6R) pale brown oil, yield: 84%: ^{1}H NMR (400 MHz, $CDCl_3$) δ 8.10 (d, J = 8.4 Hz, 1H), 7.89 – 7.80 (m, 1H), 7.77 (d, J = 8.2 Hz, 1H), 7.60 – 7.34 (m, 4H), 5.76 (s, 1H), 3.28 (d, J = 6.2 Hz, 1H), 3.10 (m, 2H), 1.63 (d, J = 6.7 Hz, 3H), 0.96 (d, J = 6.7 Hz, 2H), 0.60 (s, 6H). ^{13}C NMR (101 MHz, $CDCl_3$) δ 181.11, 137.56, 134.32, 130.75, 129.41, 128.95, 127.16, 126.33, 125.92, 123.37, 122.93, 50.50, 29.89, 28.15, 22.24, 20.03. IR (ATR): 3254.29 2866.61 1537.95. $[\alpha]_D^{20}$=-19.8 (c=0.3, MeOH), HRMS ESI pos. calcd. for $C17H23N2S^+$ $[M+H]^+$ 287.1582 found 287.1573 HRMS ESI neg. calcd. for $C17H21N2S^-$ $[M-H]^-$ 285.1425, found 285.1424,

3-[(1S)-1-(naphthalen-1-yl)ethyl]-1-[4-(trifluoromethyl)phenyl]thiourea (Compound 7S) yellow oil, yield: 84%: ^{1}H NMR (400 MHz, $CDCl_3$) δ 8.75 (s, 1H), 8.20 (d, J = 8.4 Hz, 1H), 7.88 (d, J = 8.0 Hz, 1H), 7.82 (d, J = 8.1 Hz, 1H), 7.51 (ddd, J = 33.9, 15.7, 8.2 Hz, 6H), 7.16 (d, J = 8.1 Hz, 2H), 6.38 (s, 1H), 2.12 (s, 1H), 1.77 (d, J = 6.2 Hz, 3H). ^{13}C NMR (101 MHz, $CDCl_3$) δ 178.74, 139.76, 136.61, 135.45, 133.72, 133.19, 128.72, 128.68, 127.75, 127.42, 126.73, 125.89, 125.01, 124.80, 123.28, 122.87, 122.76, 122.10, 50.69, 19.70. IR (ATR): 3295.75 3100.87 1507.10, $[\alpha]_D^{20}$= 28.3 (c=0.3, MeOH), HRMS ESI pos. calcd. for $C20H18F3N2S^+$ $[M+H]^+$

375.1143 found 375.1137 HRMS ESI neg. calcd. for $C20H16F3N2S^-$ $[M-H]^-$ 373.0986, found 373.0992.

3-[(1R)-1-(naphthalen-1-yl)ethyl]-1-[4-(trifluoromethyl)phenyl]thiourea (Compound 7R) pale yellow oil, yield: 85%: ^{1}H NMR (400 MHz, $CDCl_3$) δ 9.04 (s, 1H), 8.20 (d, J = 8.3 Hz, 1H), 7.94 – 7.83 (m, 1H), 7.80 (d, J = 8.2 Hz, 1H), 7.63 – 7.45 (m, 4H), 7.43 (d, J = 5.2 Hz, 2H), 7.20 (d, J = 8.3 Hz, 2H), 6.38 (s, 1H), 2.07 (s, 1H), 1.75 (d, J = 6.7 Hz, 3H). ^{13}C NMR (101 MHz, $CDCl_3$) δ 178.87, 139.71, 136.60, 133.77, 130.89, 128.77, 128.74, 127.89, 127.56, 126.79, 125.94, 125.05, 124.81, 123.34, 122.92, 122.11, 50.80, 19.77. IR (ATR): 3231.15 3084.91 1516.74, $[\alpha]_D^{20}$= -28.3 (c=0.3, MeOH), HRMS ESI pos. calcd. for $C20H18F3N2S^+$ $[M+H]^+$ 375.1143 found 375.1137 HRMS ESI neg. calcd. for $C20H16F3N2S^-$ $[M-H]^-$ 373.0986, found 373.0992,

3-[(1S)-1-(naphthalen-1-yl)ethyl]-1-phenylthiourea (Compound 8S) pale yellow oil, yield: 83%: ^{1}H NMR (400 MHz, $CDCl_3$) δ 8.84 (s, 1H), 8.25 (d, J = 8.5 Hz, 1H), 7.85 (dd, J = 8.1, 1.5 Hz, 1H), 7.78 (d, J = 7.5 Hz, 1H), 7.56 (ddd, J = 8.5, 6.8, 1.6 Hz, 1H), 7.50 (ddd, J = 8.1, 6.8, 1.2 Hz, 1H), 7.41 (p, J = 7.0 Hz, 2H), 7.24 (t, J = 7.7 Hz, 2H), 7.20 – 7.11 (m, 1H), 7.12 – 7.01 (m, 2H), 6.39 (s, 1H), 1.73 (d, J = 6.5 Hz, 3H). ^{13}C NMR (101 MHz, $CDCl_3$) δ 179.07, 137.05, 135.87, 133.69, 130.99, 129.85, 128.55, 128.46, 126.75, 126.62, 125.78, 124.90, 124.59, 123.51, 122.69, 50.73, 19.85. IR (ATR): 3233.07 2971.77 1508.06, $[\alpha]_D^{20}$= 78.6 (c=0.3, MeOH), HRMS ESI pos. calcd. for $C19H19N2S^+$ $[M+H]^+$ 307.1269 found 307.1261 HRMS ESI neg. calcd. for $C19H17N2S^-$ $[M-H]^-$ 305.1112, found 305.1111,

3-[(1R)-1-(naphthalen-1-yl)ethyl]-1-phenylthiourea (Compound 8R) pale yellow oil, yield: 75%: ^{1}H NMR (400 MHz, $CDCl_3$) δ 8.57 (s, 1H), 8.25 (d, J = 8.5 Hz, 1H), 7.86 (dd, J = 8.1, 1.5 Hz, 1H), 7.79 (dd, J = 7.5, 1.8 Hz, 1H), 7.54 (dddd, J = 27.7, 8.1, 6.8, 1.3 Hz, 2H), 7.47 – 7.34 (m, 2H), 7.31 – 7.21 (m, 2H), 7.20 – 7.11 (m, 1H), 7.12 – 6.97 (m, 2H), 6.39 (s, 1H), 1.74 (d, J = 6.6 Hz, 3H). ^{13}C NMR (101 MHz, $CDCl_3$) δ 179.46, 137.64, 136.55, 134.24, 131.54, 130.36, 129.89, 129.12, 129.01, 127.67, 127.17, 126.34, 126.07, 125.49, 125.10, 124.07, 123.27, 51.20, 20.45. IR (ATR): 3234.04 2971.77 1509.03, $[\alpha]_D^{20}$= -78.6 (c=0.3, MeOH), HRMS ESI pos. calcd. for $C19H19N2S^+$ $[M+H]^+$ 307.1269 found 307.1261 HRMS ESI neg. calcd. for $C19H17N2S^-$ $[M-H]^-$ 305.1112, found 305.1111,

1-benzyl-3-[(1S)-1-(naphthalen-1-yl)ethyl]thiourea (Compound 9S) pale yellow oil, yield: 75%: ^{1}H NMR (400 MHz, $CDCl_3$) δ 8.08 (d, J = 8.1 Hz, 1H), 7.85 (dd, J = 7.4, 2.0 Hz, 1H), 7.78 (d, J = 8.1 Hz, 1H), 7.60 – 7.44 (m, 2H), 7.45 – 7.27 (m, 3H), 7.14 (dd, J = 5.1, 2.1 Hz, 3H), 6.96 (s, 2H), 6.16 – 5.57 (m, 1H), 4.67 (s, 1H), 4.46 (t, J = 14.3 Hz, 2H), 1.59 (d, J = 6.7 Hz, 3H). ^{13}C NMR (101 MHz, $CDCl_3$) δ 181.00, 137.62, 137.17, 134.49, 134.24, 130.81, 129.30, 129.26, 128.90, 128.87, 128.67, 127.82, 127.44, 127.08, 126.28, 125.81, 123.22, 50.60, 48.73, 21.92. IR (ATR): 3243.68 3056.65 1531.2, $[\alpha]_D^{20}$= 39.1 (c=0.2, MeOH), HRMS ESI pos. calcd. for $C20H21N2S^+$ $[M+H]^+$ 321.1425 found 321.1411 HRMS ESI neg. calcd. for $C20H19N2S^-$ $[M-H]^-$ 319.1269, found 319.1277,

1-benzyl-3-[(1R)-1-(naphthalen-1-yl)ethyl]thiourea (Compound 9R) pale yellow oil, yield: 76%: ^{1}H NMR (400 MHz, $CDCl_3$) δ 8.08 (d, J = 8.0 Hz, 1H), 7.85 (dd, J = 7.5, 2.0 Hz, 1H), 7.77 (d, J = 8.1 Hz, 1H), 7.56 – 7.43 (m, 3H), 7.44 – 7.32 (m, 1H), 7.14 (dd, J = 5.2, 2.0 Hz, 3H), 6.97 (s, 2H), 5.88 (s, 1H), 4.65 (s, 1H), 4.46 (d, J = 18.1 Hz, 2H), 1.59 (d, J = 6.7 Hz, 3H). ^{13}C NMR (101 MHz, $CDCl_3$) δ 181.05, 137.71, 137.22, 134.17, 130.81, 129.20, 128.82, 128.76, 128.60, 127.73, 127.40, 127.07, 126.99, 126.20, 125.74, 123.15, 50.49, 48.85, 48.62, 21.77. IR (ATR): 3243.68 3055.65 1531.20, $[\alpha]_D^{20}$= -39.1 (c=0.2, MeOH), HRMS ESI pos. calcd. for $C20H21N2S^+$ $[M+H]^+$ 321.1425 found 321.1411 HRMS ESI neg. calcd. for $C20H19N2S^-$ $[M-H]^-$ 319.1269, found 319.1277,

3-[(1S)-1-(naphthalen-1-yl)ethyl]-1-[2-(trifluoromethyl)phenyl]thiourea (Compound 10S) yellow oil, yield: 84%: ^{1}H NMR (400 MHz, $CDCl_3$) δ 8.17 (d, J = 8.5 Hz, 1H), 7.85 (d, J = 8.2 Hz, 1H), 7.78 (d, J = 8.2 Hz, 1H), 7.67 – 7.45 (m, 3H), 7.45 – 7.18 (m, 3H), 6.24 (br s, 1H), 1.73 (d, J = 6.7 Hz, 3H). ^{13}C NMR (101 MHz, $CDCl_3$) δ 180.04, 136.52, 133.58, 132.73, 130.76, 128.97, 128.49, 127.97, 127.27, 126.85, 126.58, 126.51, 125.71, 124.89, 124.13, 123.20, 122.73, 121.41, 50.85, 19.48. IR (ATR): 3209.93 3044.09 1539.88, $[\alpha]_D^{20}$= 16.0 (c=0.3, MeOH), HRMS ESI pos. calcd. for $C20H18F3N2S^+$ $[M+H]^+$ 375.1143 found 375.1145 HRMS ESI neg. calcd. for $C20H16F3N2S^-$ $[M-H]^-$ 373.0986, found 373.0990,

3-[(1R)-1-(naphthalen-1-yl)ethyl]-1-[2-(trifluoromethyl)phenyl]thiourea (Compound 10R) yellow oil, yield: 83%: ^{1}H NMR (400 MHz, $CDCl_3$) δ 8.18 (d, J = 8.5 Hz, 1H), 7.81 (m, 2H), 7.67 – 7.13 (m, 6H), 6.29 (br s, 1H), 1.73 (d, J = 6.6 Hz, 3H). ^{13}C NMR (101 MHz, $CDCl_3$) δ 180.11, 136.56, 133.53, 132.71, 130.75, 129.07, 128.40, 127.91, 127.22, 126.84, 126.57, 126.43, 125.64, 124.86, 124.12, 123.18, 122.69, 121.40, 50.77, 19.45. IR (ATR): 3209.93 3044.89 1538.92, $[\alpha]_D^{20}$= -16.0 (c=0.3, MeOH), HRMS ESI pos. calcd. for $C20H18F3N2S^+$ $[M+H]^+$ 375.1143 found 375.1145 HRMS ESI neg. calcd. for $C20H16F3N2S^-$ $[M-H]^-$ 373.0986, found 373.0990.

Supplementary Table S1. Enantioseparation results obtained on amylose-type stationary phases using various eluents.

Analyte	Eluent	Lux Amylose-1				Lux Amylose-2			
		t_1	t_2	Rs	EEO	t_1	t_2	Rs	EEO
**1**	MeOH	3.783	4.052	1.48	R-S	4.009	4.009	0.00	n.s.
	ACN	4.532	4.532	0.00	n.s.	4.423	4.423	0.00	n.s.
	2-PrOH	3.594	3.594	0.00	n.s.	4.097	4.097	0.00	n.s.
**2**	MeOH	4.003	4.520	2.51	R-S	4.077	4.077	0.00	n.s.
	ACN	5.380	5.380	0.00	n.s.	4.639	5.132	1.78	S-R
	2-PrOH	3.705	3.705	0.00	n.s.	4.185	4.185	0.00	n.s.
**3**	MeOH	4.789	6.333	4.29	R-S	4.333	4.333	0.00	n.s.
	ACN	5.868	5.868	0.00	n.s.	5.482	6.425	1.92	R-S
	2-PrOH	4.091	4.091	0.00	n.s.	4.703	4.703	0.00	n.s.
**4**	MeOH	5.411	5.684	0.74	R-S	4.434	4.813	1.25	S-R
	ACN	5.338	5.512	0.39	R-S	9.951	18.123	3.33	S-R
	2-PrOH	4.808	4.808	0.00	n.s.	5.127	5.127	0.00	n.s.
**5**	MeOH	4.885	7.627	4.55	R-S	4.070	4.314	1.36	R-S
	ACN	4.851	6.842	3.40	R-S	4.343	6.840	1.45	R-S
	2-PrOH	5.855	6.513	1.20	S-R	4.601	4.601	0.00	n.s.
**6**	MeOH	3.963	4.296	1.57	R-S	4.041	4.041	0.00	n.s.
	ACN	5.217	5.217	0.00	n.s.	4.635	4.635	0.00	n.s.
	2-PrOH	3.728	3.728	0.00	n.s.	4.115	4.115	0.00	n.s.
**7**	MeOH	4.409	6.544	3.34	R-S	3.946	3.946	0.00	n.s.
	ACN	4.437	5.193	1.41	R-S	8.234	8.234	0.00	n.s.
	2-PrOH	4.281	4.281	0.00	n.s.	4.373	4.373	0.00	n.s.
**8**	MeOH	4.963	7.136	4.45	R-S	4.381	4.381	0.00	n.s.
	ACN	4.793	5.744	2.27	R-S	6.231	6.688	0.96	R-S
	2-PrOH	4.870	6.181	3.42	R-S	5.036	5.036	0.00	n.s.
**9**	MeOH	5.618	6.201	1.21	R-S	4.255	4.255	0.00	n.s.
	ACN	3.762	3.762	0.00	n.s.	5.533	6.415	1.53	S-R
	2-PrOH	4.270	4.270	0.00	n.s.	4.620	4.620	0.00	n.s.
**10**	MeOH	3.908	3.908	0.00	n.s.	3.978	3.978	0.00	n.s.
	ACN	3.760	3.760	0.00	n.s.	4.364	4.364	0.00	n.s.
	2-PrOH	3.715	3.997	1.10	S-R	4.256	4.256	0.00	n.s.

Notes: t1 and t2 are retention times (min) of the first- and second-eluting enantiomer, respectively; Rs is chromatographic resolution; EEO: enantiomer elution order (R-S means R is the first eluted enantiomer, S-R means S is the first eluted enantiomer); n.s., not separated (Rs = 0).

Supplementary Table S2. Enantioseparation results obtained on cellulose-based stationary phases using various eluents.

Analyte	Eluent	Lux Cellulose-1				Lux Cellulose-2				Lux Cellulose-3				Lux Cellulose-4			
		t1	t2	Rs	EEO	t1	t2	Rs	EEO	t1	t2	Rs	EEO	t1	t2	Rs	EEO
**1**	MeOH	4.533	4.533	0.00	n.s.	4.032	4.032	0.00	n.s.	4.752	4.752	0.00	n.s.	4.148	4.148	0.00	n.s.
	ACN	4.460	4.460	0.00	n.s.	4.538	4.733	0.71	S-R	4.219	4.219	0.00	n.s.	4.698	4.698	0.00	n.s.
	2-PrOH	4.386	4.386	0.00	n.s.	4.069	4.069	0.00	n.s.	4.325	4.325	0.00	n.s.	4.217	4.467	0.91	S-R
**2**	MeOH	4.795	4.927	0.68	R-S	4.175	4.306	0.81	S-R	4.734	4.734	0.00	n.s.	4.238	4.350	0.74	S-R
	ACN	4.896	4.896	0.00	n.s.	4.737	5.015	1.18	S-R	4.274	4.274	0.00	n.s.	4.880	5.235	0.76	S-R
	2-PrOH	4.667	4.991	1.06	R-S	4.258	4.258	0.00	n.s.	4.408	4.408	0.00	n.s.	4.355	4.695	1.19	S-R
**3**	MeOH	5.842	5.842	0.00	n.s.	4.699	4.918	1.19	S-R	5.486	5.898	1.47	R-S	4.674	4.856	1.22	S-R
	ACN	4.990	4.990	0.00	n.s.	5.129	5.411	1.17	S-R	4.378	4.378	0.00	n.s.	5.158	5.511	0.82	S-R
	2-PrOH	5.657	6.139	1.25	R-S	5.063	5.063	0.00	n.s.	5.159	5.159	0.00	n.s.	5.127	5.665	1.36	S-R
**4**	MeOH	6.134	6.397	0.83	R-S	4.522	4.934	2.32	S-R	6.434	19.159	14.68	S-R	4.487	4.841	2.14	S-R
	ACN	6.350	8.211	3.20	S-R	6.153	9.226	5.39	S-R	4.754	6.041	4.11	S-R	5.953	8.846	4.27	S-R
	2-PrOH	6.203	6.502	0.68	R-S	4.627	5.627	1.79	S-R	5.303	6.283	1.78	S-R	4.615	6.057	3.03	S-R
**5**	MeOH	5.997	6.885	3.02	R-S	4.692	4.692	0.00	n.s.	6.920	7.173	0.68	R-S	4.634	4.634	0.00	n.s.
	ACN	6.161	8.512	3.86	R-S	6.389	8.423	3.96	R-S	4.761	5.871	3.51	R-S	6.172	7.811	2.82	R-S
	2-PrOH	5.634	7.223	3.09	R-S	4.880	5.381	0.87	R-S	5.183	6.127	1.88	R-S	5.254	5.254	0.00	n.s.
**6**	MeOH	4.873	4.873	0.00	n.s.	4.121	4.121	0.00	n.s.	4.561	4.561	0.00	n.s.	4.203	4.303	0.74	S-R
	ACN	4.655	4.655	0.00	n.s.	4.636	4.945	1.30	S-R	4.112	4.112	0.00	n.s.	4.771	5.167	0.86	S-R
	2-PrOH	4.734	7.981	0.83	R-S	4.135	4.135	0.00	n.s.	4.283	4.283	0.00	n.s.	4.234	4.543	1.08	S-R
**7**	MeOH	6.043	6.043	0.00	n.s.	3.955	3.955	0.00	n.s.	5.006	5.384	1.44	S-R	3.988	4.186	1.41	S-R
	ACN	8.197	8.197	0.00	n.s.	7.304	7.304	0.00	n.s.	5.640	5.640	0.00	n.s.	7.058	8.500	1.51	S-R
	2-PrOH	5.142	6.751	3.23	R-S	4.273	4.273	0.00	n.s.	4.978	4.978	0.00	n.s.	4.281	4.811	1.46	S-R
**8**	MeOH	5.532	5.959	1.68	R-S	4.620	4.620	0.00	n.s.	6.268	6.941	2.00	R-S	4.663	4.917	1.50	S-R
	ACN	5.854	5.854	0.00	n.s.	5.724	6.141	1.13	S-R	4.820	4.820	0.00	n.s.	5.704	6.456	1.70	S-R
	2-PrOH	5.623	6.588	2.22	R-S	5.611	5.611	0.00	n.s.	5.595	5.595	0.00	n.s.	5.667	6.960	2.43	S-R
**9**	MeOH	5.834	6.005	0.56	S-R	4.507	4.633	0.68	R-S	4.730	4.730	0.00	n.d.	3.783	3.783	0.00	n.s.
	ACN	3.963	3.963	0.00	n.s.	4.703	4.884	0.63	R-S	3.979	3.979	0.00	n.s.	4.014	4.014	0.00	n.s.
	2-PrOH	3.573	3.573	0.00	n.s.	4.673	4.673	0.00	n.s.	5.018	5.018	0.00	n.s.	3.726	3.726	0.00	n.s.
**10**	MeOH	4.715	4.877	0.78	R-S	3.961	3.961	0.00	n.s.	4.999	4.999	0.00	n.s.	4.061	4.061	0.00	n.s.
	ACN	4.557	4.557	0.00	n.s.	4.097	4.502	1.00	S-R	4.031	4.031	0.00	n.s.	4.113	4.551	0.83	S-R
	2-PrOH	4.864	5.601	2.03	R-S	4.169	4.169	0.00	n.s.	4.696	4.696	0.00	n.s.	4.244	4.244	0.00	n.s.

Notes: t1 and t2 are retention times (min) of the first- and second-eluting enantiomer, respectively; Rs is chromatographic resolution; EEO: enantiomer elution order (R-S means R is the first eluted enantiomer, S-R means S is the first eluted enantiomer); n.s., not separated (Rs = 0); n.d., not determined.

Supporting Information S2. Analysis-Time Distribution across Eluents and Stationary Phases

For the evaluation of throughput, the analysis time was defined as the retention time of the second-eluting enantiomer for each condition (t_{last}). Across the full dataset (180 conditions; 10 analytes × 3 eluents × 6 stationary phases), analysis time ranged from 3.57 to 19.16 min, with an overall mean of 5.33 min and a median of 4.83 min (IQR: 4.29 – 5.87 min), indicating that most separations were completed within ~6 min, while a limited number of conditions produced markedly longer runs. Overall, the results underscore one of the main advantages of the polar organic mode: fast enantioseparations and short analysis times.

A clear eluent-dependent trend was observed. On average, 2-propanol provided the shortest analysis times (mean 4.97 min; max 7.98 min; SD 0.99 min) and the narrowest distribution, suggesting more consistent run times under these conditions. Acetonitrile yielded the longest runs on average (mean 5.83 min; max 18.12 min; SD 2.16 min), while methanol was intermediate (mean 5.19 min) but contained the single longest run (19.16 min) (Supplementary Figure 1). The different eluent effects likely arise from the markedly different hydrogen-bonding properties of acetonitrile and different alcohols, which modulate analyte–selector interactions. Stationary-phase-specific statistics showed comparable mean run times overall (means ~5.07–5.81 min), with Lux Cellulose-2 being the fastest on average (mean 5.07 min) and Lux Cellulose-1 the slowest (mean 5.81 min). However, the maximum run times were dominated by distinct outliers, primarily observed on Lux Cellulose-3 (max 19.16 min) and Lux Amylose-2 (max 18.12 min). When combining eluent and stationary phase effects, the slowest average combination was acetonitrile on Lux Amylose-2 (mean 7.13 min), whereas several faster combinations clustered around ~4.2–4.7 min (e.g., methanol on Lux Amylose-2/Lux Cellulose-2/Lux Cellulose-4; 2-propanol on Lux Amylose-1/Lux Amylose-2/Lux Cellulose-2). At the analyte level, Compound **4,** containing iodophenyl substitution, exhibited the highest mean analysis time (7.56 min) and was responsible for the most extreme run times, underscoring that both compound-dependent retention and method conditions contribute to throughput in this screening set.

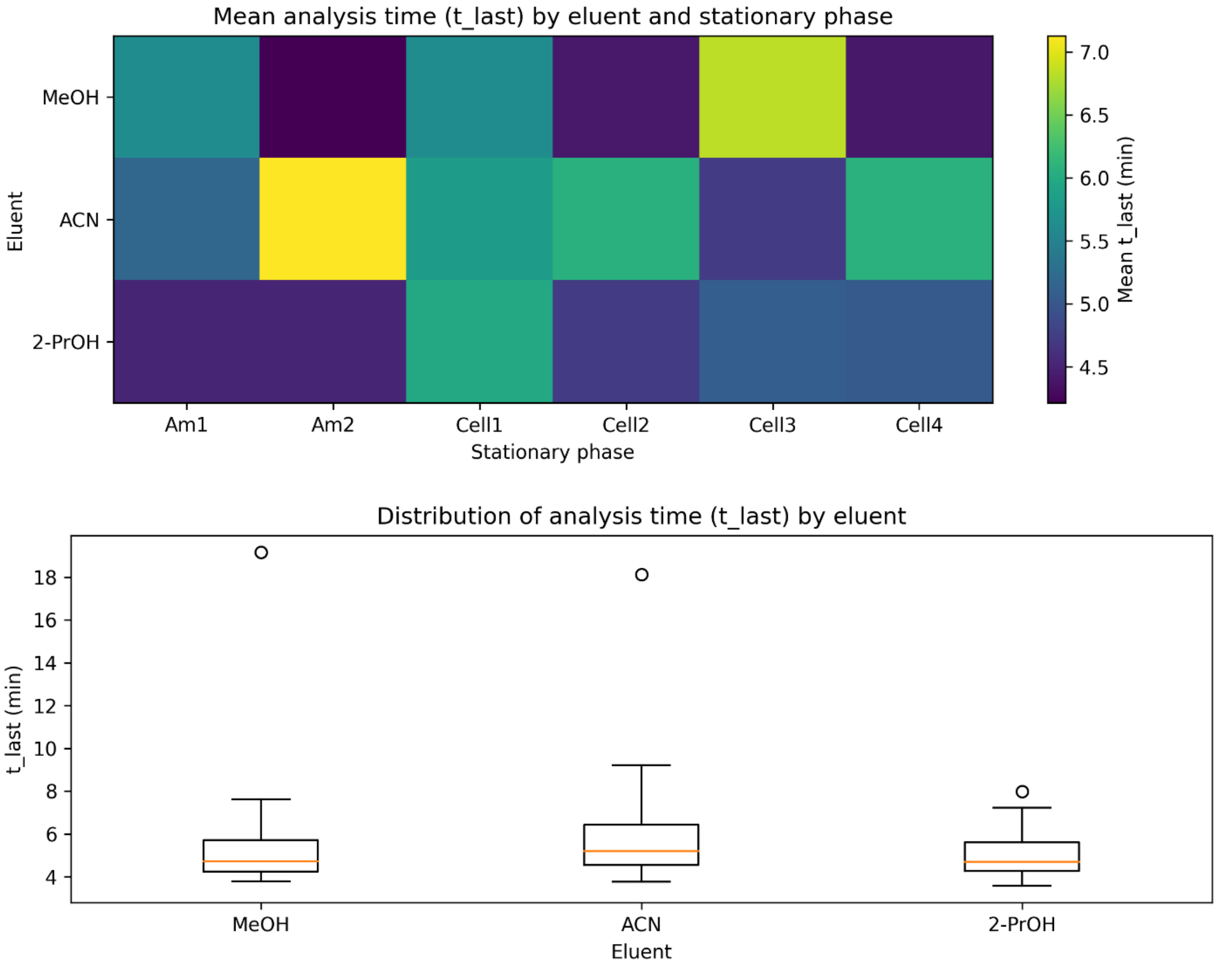

Supplementary Figure S1. *Analysis-time overview across eluents and stationary phases in polar organic mode.* **Top:** Heatmap of the mean analysis time, defined as the retention time of the
second-eluting enantiomer $t_{\text{last}} = \max(t_1,\ t_2)$, for each eluent–stationary phase combination (values averaged over all analytes). **Bottom:** Box-and-whisker plots of t_{last} grouped by eluent; the central line denotes the median, the box the interquartile range (25th–75th percentiles), whiskers the data range, and points represent outliers. Eluents: MeOH, ACN, and 2-PrOH; stationary phases: Am1, Am2, and Cell1–Cell4. Analysis times are given in minutes.

Supporting Information S3. Enantiomer Elution-Order Reversal

The reversal of the enantiomeric elution order is crucial, as changes in EEO indicate alterations in enantiorecognition capabilities. Therefore, studying EEO reversals can provide deeper insights into designing and understanding chiral separations. According to the literature, changes in any chromatographic parameters can lead to EEO reversal but they can be broadly categorized into three main types: chiral selector-based EEO reversal, mobile phase-dependent EEO reversal, and temperature-dependent EEO reversal. Temperature-dependent EEO reversal was not detected in our study. It is unsurprising that altering the chiral selector can invert the EEO; even changes to

the selector’s backbone or substituents can yield different EEOs. By contrast, mobile-phase–dependent EEO reversals are more intriguing and comparatively uncommon. Heatmap of elution-order bias across eluents and chiral stationary phases is summarized in Supplementary Figure 2. The data indicate that elution order is not randomly distributed among the investigated CSP–eluent combinations. Am1 and Cell1 tend to favor R–S elution under selected conditions, whereas Cell4 consistently favors the opposite S–R order. The observed differences emphasize the combined role of selector structure and eluent composition in determining enantiomer elution order.

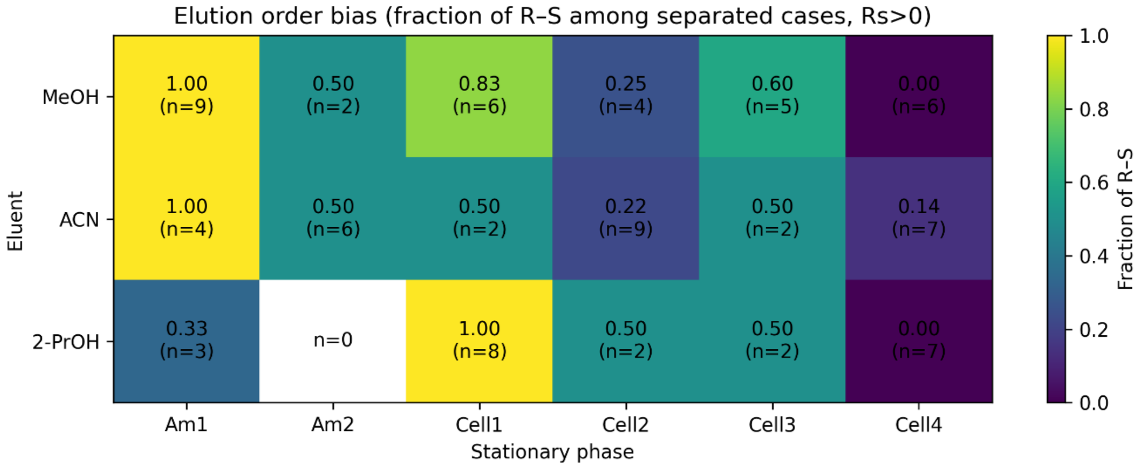

Supplementary Figure S2. Heatmap of elution-order bias across eluents and chiral stationary phases. The color scale represents the **fraction of R–S elution order** among **separated cases only** $(R_s > 0)$ for each eluent–CSP combination, where 1.00 indicates exclusively R–S and 0.00 indicates exclusively S–R. Values in each cell are reported as the R–S fraction, with the number of contributing observations shown in parentheses (n). Cells with $n = 0$ indicate that no separations were obtained under the corresponding conditions. Eluents: MeOH, ACN, and 2-PrOH; CSPs: Am1, Am2, and Cell1–Cell4.

Some examples of stationary-phase-dependent EEO reversals can be observed in Supplementary Figures 3 and 4.

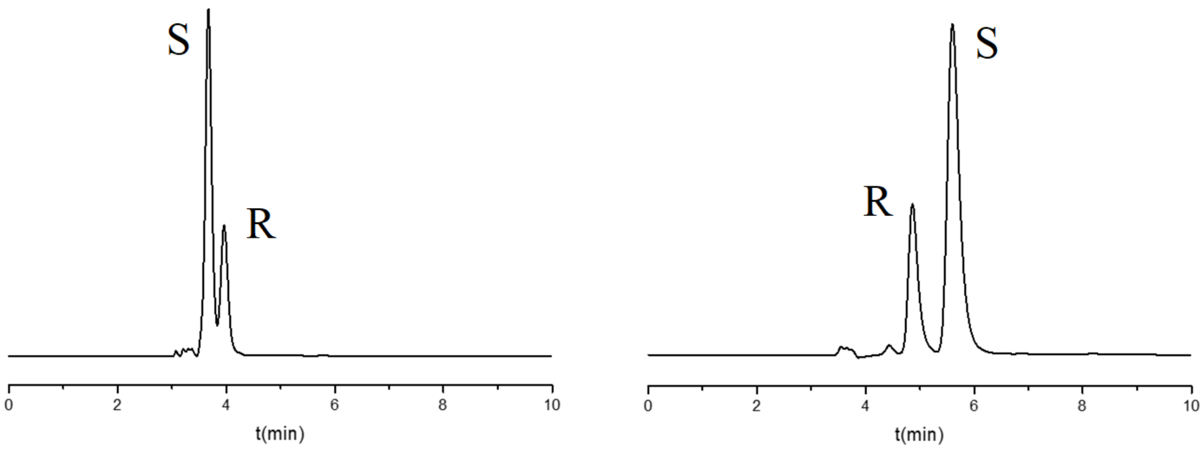

Supplementary Figure S3. Reversal of EEO caused by a different selector backbone in the case of compound **10** in a neat 2-propanol eluent, between a Lux Amylose-1 CSP (left) and a Lux Cellulose-1 CSP (right).

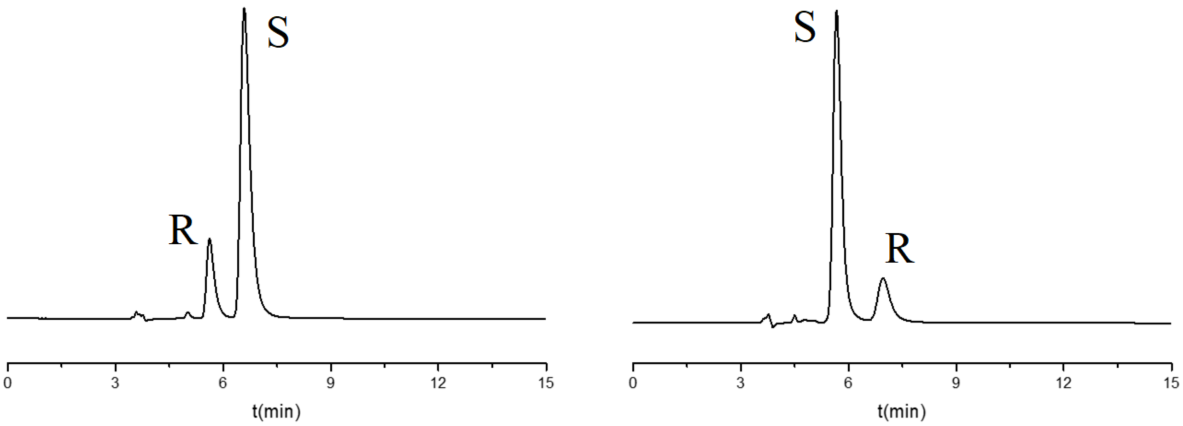

Supplementary Figure S4. Reversal of EEO caused by a different substituent in the case of compound **8** in a neat 2-propanol eluent, between a Lux Cellulose-1 CSP (left) and a Lux Cellulose-4 CSP (right).

In our study, mobile-phase–dependent EEO reversal was observed in two cases. For enantiomer pair **5** on the Amylose-1 CSP, the EEO was R → S with MeOH and ACN, but switched to S → R with 2-PrOH. Similarly, for enantiomer pair **4** on the Cellulose-1 CSP, the S-enantiomer eluted before the R-enantiomer in ACN, whereas in MeOH and 2-PrOH the order inverted (R → S). These EEO reversals can be seen in Supplementary Figures 5 and 6.

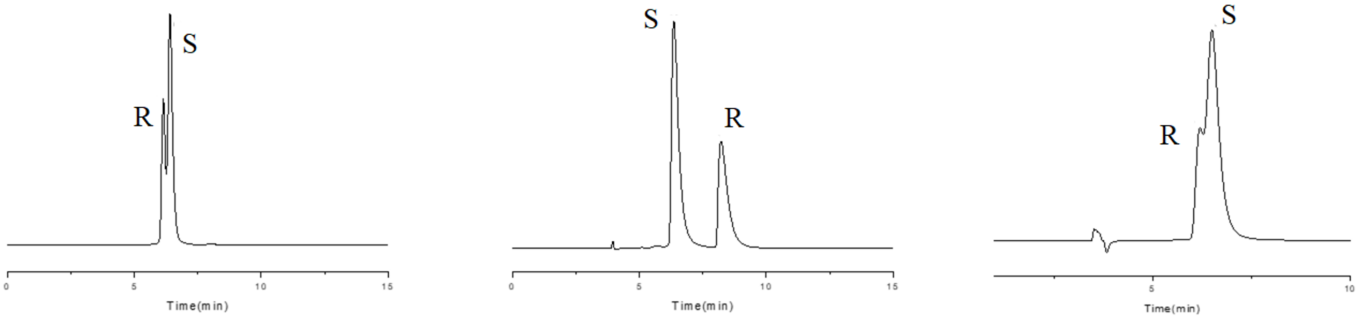

Supplementary Figure S5. Chromatograms of compound **4** in methanol (left), acetonitrile (middle) and 2-propanol (right) on the Lux Cellulose-1.

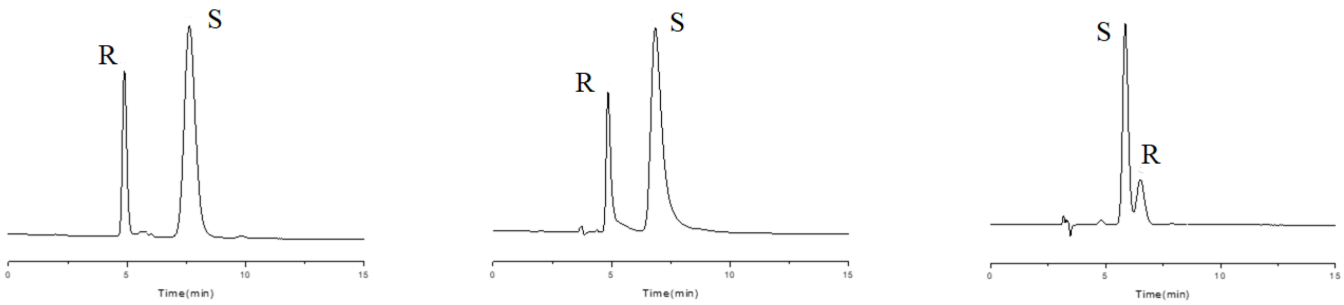

Supplementary Figure S6. Chromatograms of compound **5** in methanol (left), acetonitrile (middle) and 2-propanol (right) on the Lux Amylose-1.

Supplementary Table S3. Predicted physicochemical, ADME and toxicity-related parameters of the investigated thiourea derivatives.

Compound	pKa	logP	logS	logBB	CYP3A4	CYP2C9	CYP1A2	PPB (%)	HSA	Pe_Caco	Bioav.	LD50 mouse oral	LD50 rat oral
1	12.95	3.77	-5.31	0.40	0.88	0.16	0.62	93.12	4.61	73.84	45.55	2.63	2.53
2	13.01	3.92	-5.59	0.40	0.87	0.17	0.56	94.33	4.54	68.66	31.25	2.58	2.68
3	12.93	4.34	-5.94	0.41	0.89	0.37	0.60	96.41	4.59	51.41	39.90	2.61	2.59
4	11.40	5.36	-6.71	0.25	0.94	0.41	0.17	98.89	5.05	20.82	10.97	2.80	2.67
5	8.85	6.03	-7.62	-0.27	0.95	0.56	0.05	99.72	5.64	10.63	6.57	2.82	2.44
6	12.99	4.24	-5.72	0.54	0.91	0.18	0.58	94.64	4.64	55.97	32.96	2.56	2.73
7	10.85	5.00	-6.71	-0.15	0.94	0.28	0.27	99.47	5.51	29.57	8.60	2.84	2.43
8	12.14	4.59	-6.07	0.05	0.90	0.52	0.31	98.79	5.08	42.99	36.00	2.78	2.33
9	12.97	4.39	-6.11	0.30	0.93	0.41	0.25	97.44	5.38	49.48	17.97	2.91	2.48
10	10.01	5.33	-6.38	-0.11	0.96	0.34	0.33	99.51	5.36	21.61	32.85	2.64	2.31

Abbreviations: PPB, plasma protein binding; HSA, human serum albumin binding; Pe_Caco, predicted Caco-2 permeability; Bioav., predicted oral bioavailability; LD50 mouse oral and LD50 rat oral, predicted oral median lethal dose values for mouse and rat, respectively. Values are shown as exported from the prediction workflow and rounded to two decimal places.
